# Supplementary material for: Systems‐level transcriptional regulation of Caenorhabditis elegans metabolism
Source: Mol Syst Biol. 2023 Mar 21;19(5):e11443. doi: 10.15252/msb.202211443 (PMC10167481; doi:10.15252/msb.202211443)
Supplement: Supplementary file 1 — Appendix [file MSB-19-e11443-s004.pdf]

APPENDIX

Systems-level transcriptional regulation of *Caenorhabditis elegans* metabolism

Shivani Nanda[1], Marc-Antoine Jacques[1,3], Wen Wang[2], Chad L Myers[2], L. Safak Yilmaz[1], Albertha JM Walhout[1*]

1. Department of Systems Biology, University of Massachusetts Chan Medical School, Worcester, MA 01655, United States

2. Department of Computer Science and Engineering, University of Minnesota, Minneapolis, MN 55455, United States

3. Current address: Cancer Research UK Cambridge Institute, University of Cambridge, Cambridge CB2 0RE, United Kingdom; and EMBL's European Bioinformatics Institute (EBI), Cambridgeshire CB10 1SD United Kingdom

CONTENTS

Supplementary Figures

Appendix Figure S1……………………………………..…………………..……..…3
Appendix Figure S2……………………….…...……….…………………………………4
Appendix Figure S3…………………….……………………….………………………6
Appendix Figure S4…………………………..……………....…………………………7
Appendix Figure S5……………………...……………………..…….…………………………9

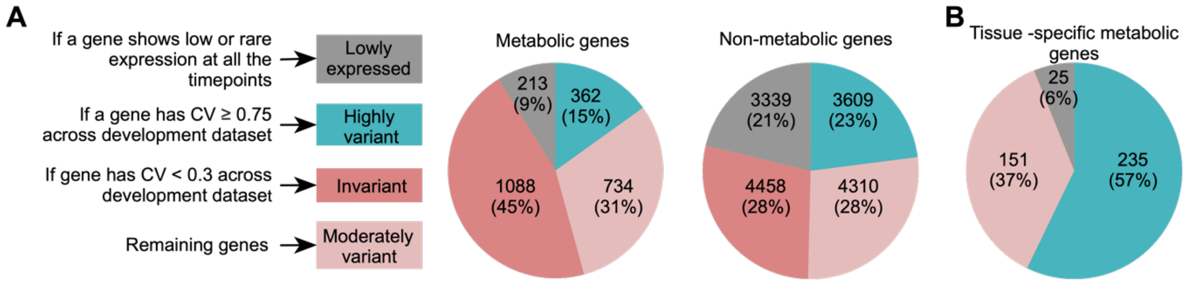

Appendix Figure S1. Transcriptional Regulation of Metabolism Across Development

(A) Diagram showing alternative criterion using CV for categorizing metabolic and non-metabolic genes into four categories across development: lowly expressed, invariant, moderately variant and highly variant (left). This analysis was done to make sure the result that metabolic genes are more highly variant across tissues than development is not driven by the use of different statistical approaches (*i.e.*, CV in tissue data set and VS in development). To eliminate this effect, we reevaluated the development dataset with the CV approach using the same threshold as with tissues, and found that, the percentage of highly variant genes during development was lowered from 31% to 15% with this method. Thus, using the same metric resulted in an even greater difference between metabolic genes exhibiting variation across tissues versus those exhibiting variation during development. Pie charts showing metabolic (center) and non-metabolic (right) gene expression variation in the development dataset using CV as criterion.

(B) Pie chart showing percentage of tissue-specific genes showing high variation in expression across development dataset.

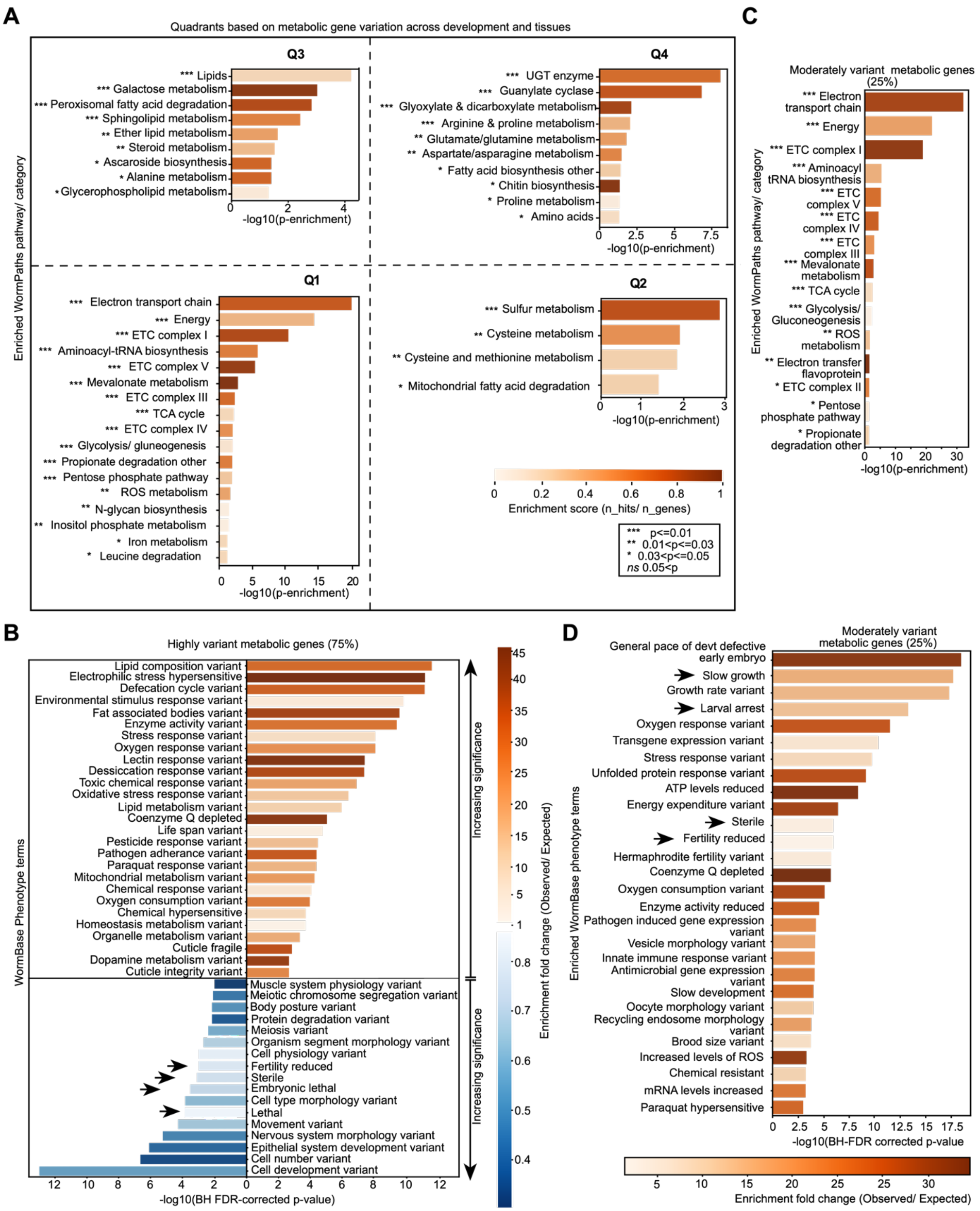

Appendix Figure S2. Pathway and Phenotypic Enrichment Analysis of Metabolic Genes Based on Expression Variation

(A) Bar graph showing enriched WormPaths pathways/categories for iCEL1314 genes in the four quadrants Q1, Q2, Q3 and Q4 in Figure 1H. The significance levels are indicated by asterisks or '*ns*'(not significant). Not significant (*ns)* pathways are not shown.

(B) Bar graph showing phenotypes enriched for highly variant metabolic genes (FDR-corrected p-value≤0.001).

(C) Bar graph showing WormPaths pathways/ categories enriched for moderately variant metabolic. The significance levels legend as indicated in (A).

(D) Bar graph showing phenotypes enriched for moderately variant metabolic genes (FDR-corrected p-value≤0.001)

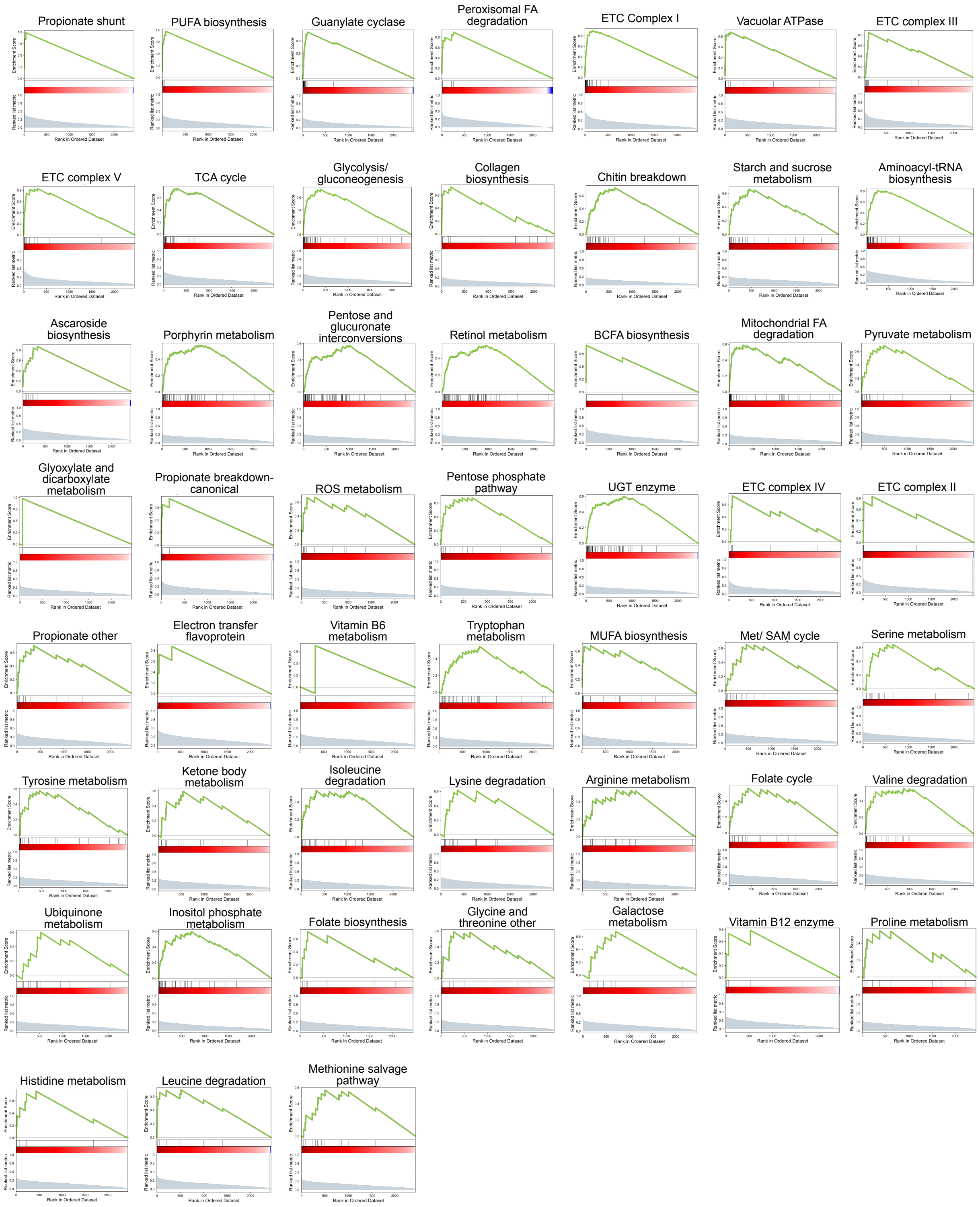

Appendix Figure S3. Mountain Plots Showing WormPaths Pathways Significantly Enriched For Coexpression in Order of Decreasing Significance

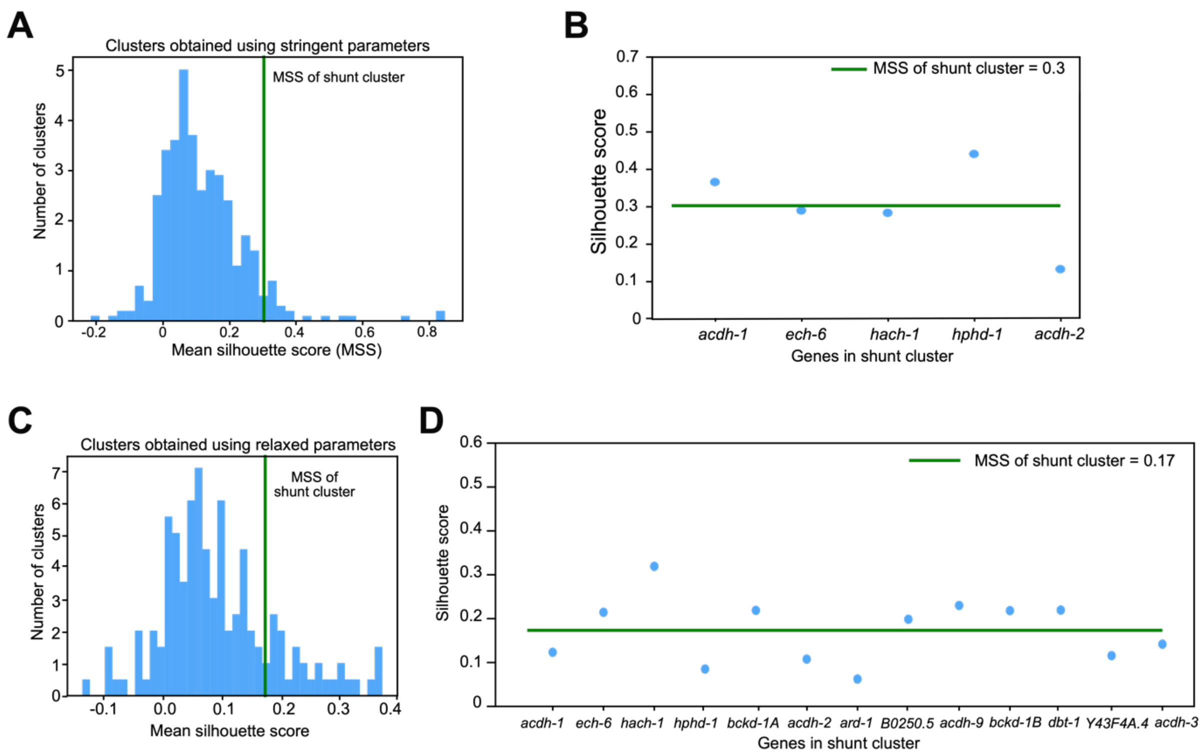

Appendix Figure S4. Mean Silhouette Score (MSS) to Evaluate Cluster Quality of Stringent and Relaxed Clusters

(A) Plot showing the distribution of the MSS of all clusters obtained using dynamic cut tree algorithm with stringent parameters (deepSplit=2, minClusterSize=3). The green vertical line shows the MSS of shunt cluster.

(B) Scatter plot with individual silhouette scores of genes in the propionate shunt cluster, with stringent parameters. MSS of this pathway is shown.

(C) Plot showing the distribution of the MSS of all clusters obtained using dynamic cut tree algorithm with relaxed parameters (deepSplit=3, minClusterSize=6). The green vertical line shows the MSS of shunt cluster.

(D) Scatter plot with individual silhouette scores of the cluster containing propionate shunt genes, with relaxed parameters. MSS of this pathway is shown along with the overall MSS threshold.

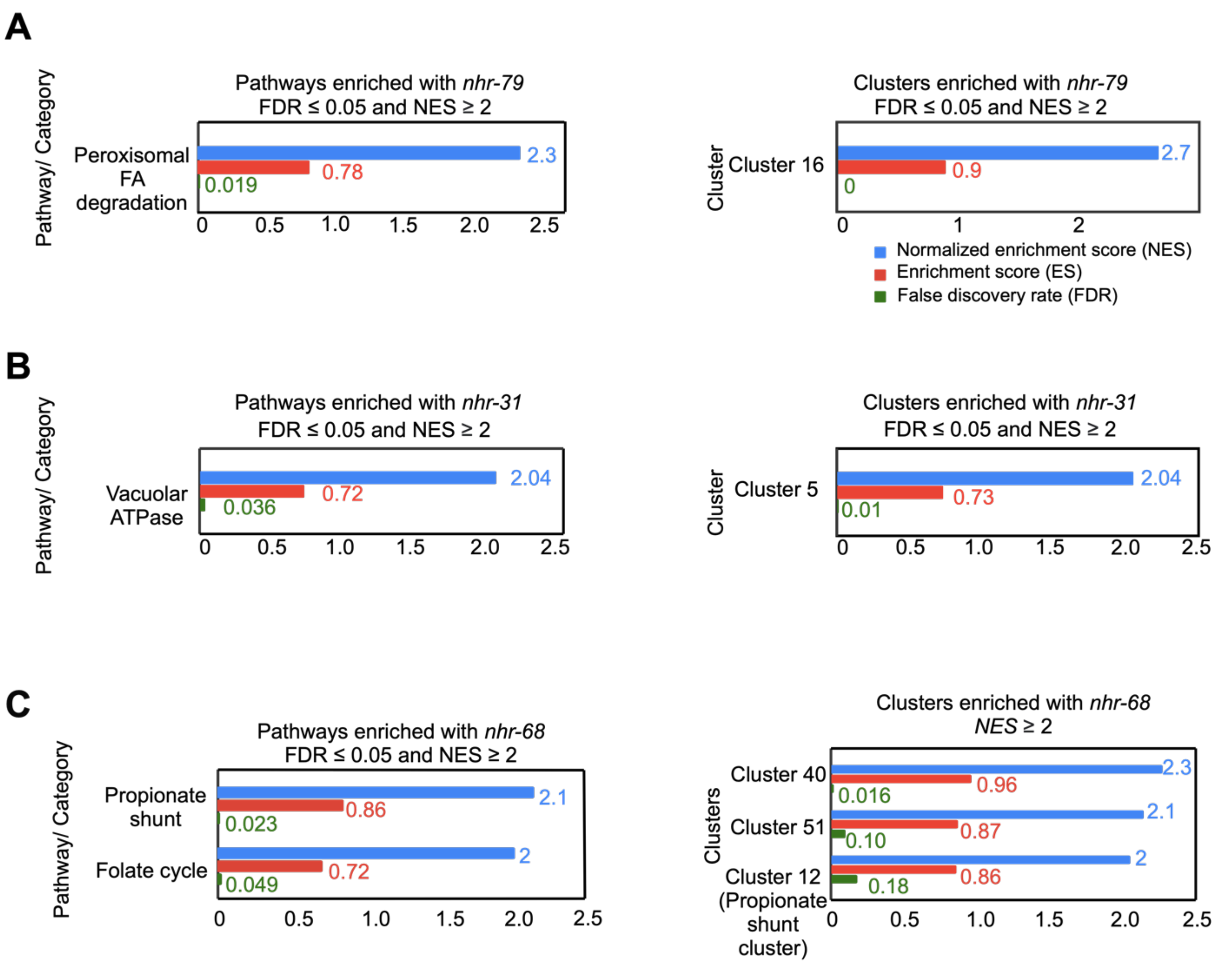

Appendix Figure S5. Enrichment of TFs to (Sub-)Pathways

(A) Plot showing enrichment to coexpression of *nhr-79* with peroxisomal fatty acid degradation and cluster 16 (NES≥2, FDR≤0.05).

(B) Plot showing enrichment to coexpression of *nhr-31* with vacuolar ATPases and cluster 5(NES≥2, FDR≤0.05).

(C) Plot showing enrichment to coexpression of *nhr-68* with propionate shunt (NES≥2, FDR≤0.05) and cluster 12(NES≥2).
