# Supplementary material for: Systems‐level transcriptional regulation of Caenorhabditis elegans metabolism
Source: Mol Syst Biol. 2023 Mar 21;19(5):e11443. doi: 10.15252/msb.202211443 (PMC10167481; doi:10.15252/msb.202211443)
Supplement: Supplementary file 2 — Expanded View Figures PDF [file MSB-19-e11443-s001.pdf]

Expanded View Figures

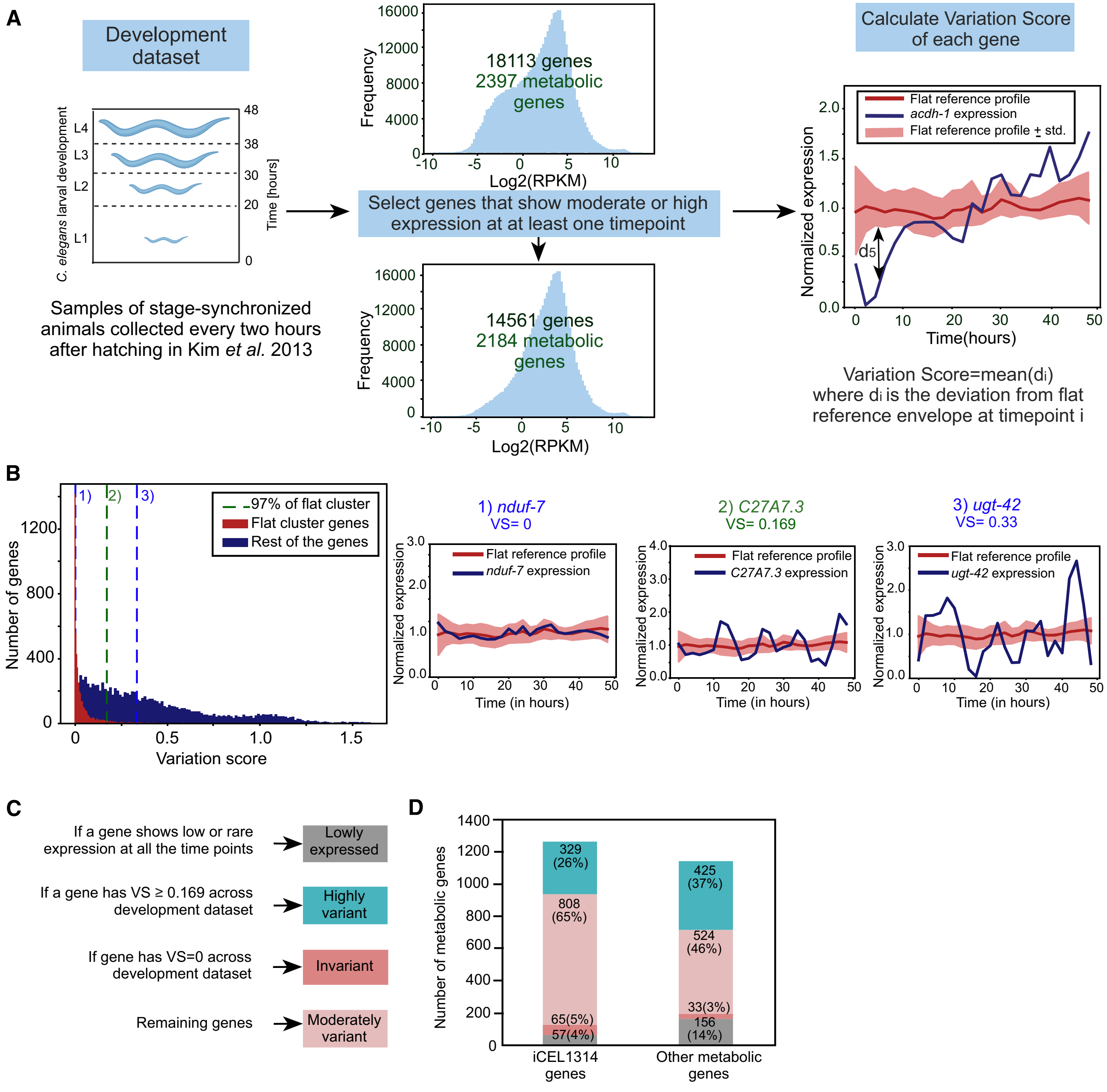

Figure EV1.

22 of 21 *Molecular Systems Biology* **19**: e11443 | 2023 © 2023 The Authors

Figure EV1. Transcriptional regulation of metabolism during development.

A Computational pipeline to identify highly variant metabolic genes during development. Genes that showed either moderate or high expression in at least one time point were selected, reducing the number of genes from 18,113 (2,397 metabolic) to 14,561 (2,184 metabolic). For each gene, VS was calculated using the deviation from a flat reference profile at each time point (see Materials and Methods). The red line indicates the mean value and light red shaded area the standard deviation of the flat reference profile. The profile of *acdh-1* is plotted in blue as an example of a developmentally regulated gene. d_5 denotes the deviation of *acdh-1* expression from the flat reference profile at the 5th data point.

B Distribution of VS of genes belonging to the flat cluster versus those belonging to other clusters. The vertical lines at 1 and 3 represent the iCEL1314 genes *nduf-7* and *ugt-42*, which have the lowest and highest VS of the flat set, respectively. The vertical line at 2 indicates the gene *C27A7.3* with selected threshold of VS at the 97% quantile of the flat cluster (VS = 0.169).

C Diagram showing criterion of categorizing metabolic and nonmetabolic genes into four categories across development: lowly expressed, invariant, moderately variant and highly variant.

D Bar graph shows the distinction of low expressed, invariant, moderately variant and highly variant genes during development in iCEL1314 and other metabolic genes. Color legend as indicated in (C).

Figure EV2. Transcriptional regulation of metabolic genes across different tissues.

A Computational pipeline to determine highly variant metabolic genes across tissues. Genes that show either moderate or high expression in at least one tissue are selected for analysis reducing the number of *C. elegans* genes from 19,675 (2,491 metabolic) to 13,305 (2,143 metabolic). The coefficient of variation (CV) of each gene was calculated by dividing the standard deviation of expression across tissues by the mean expression. Different thresholds of CV were titrated to select a stringent CV to categorize genes as variant and therefore potentially transcriptionally regulated. Examples for each threshold are provided in (B).

B Tissue expression and CV values of propionate shunt genes.

C Bar graphs showing expression profiles of example genes across tissues with CV 0.15, 0.3, 0.45, 0.6, 0.75, 0.9, 1.05 and 1.2. Numbering of examples is according to the corresponding threshold lines in (A).

D Diagram showing criterion of categorizing metabolic and nonmetabolic genes into four categories across tissues: lowly expressed, invariant, moderately variant and highly variant.

E Bar graph shows the distinction of low expressed, invariant, moderately variant and highly variant genes across tissues in iCEL1314 and other metabolic genes. Color legend as indicated in (D).

© 2023 The Authors

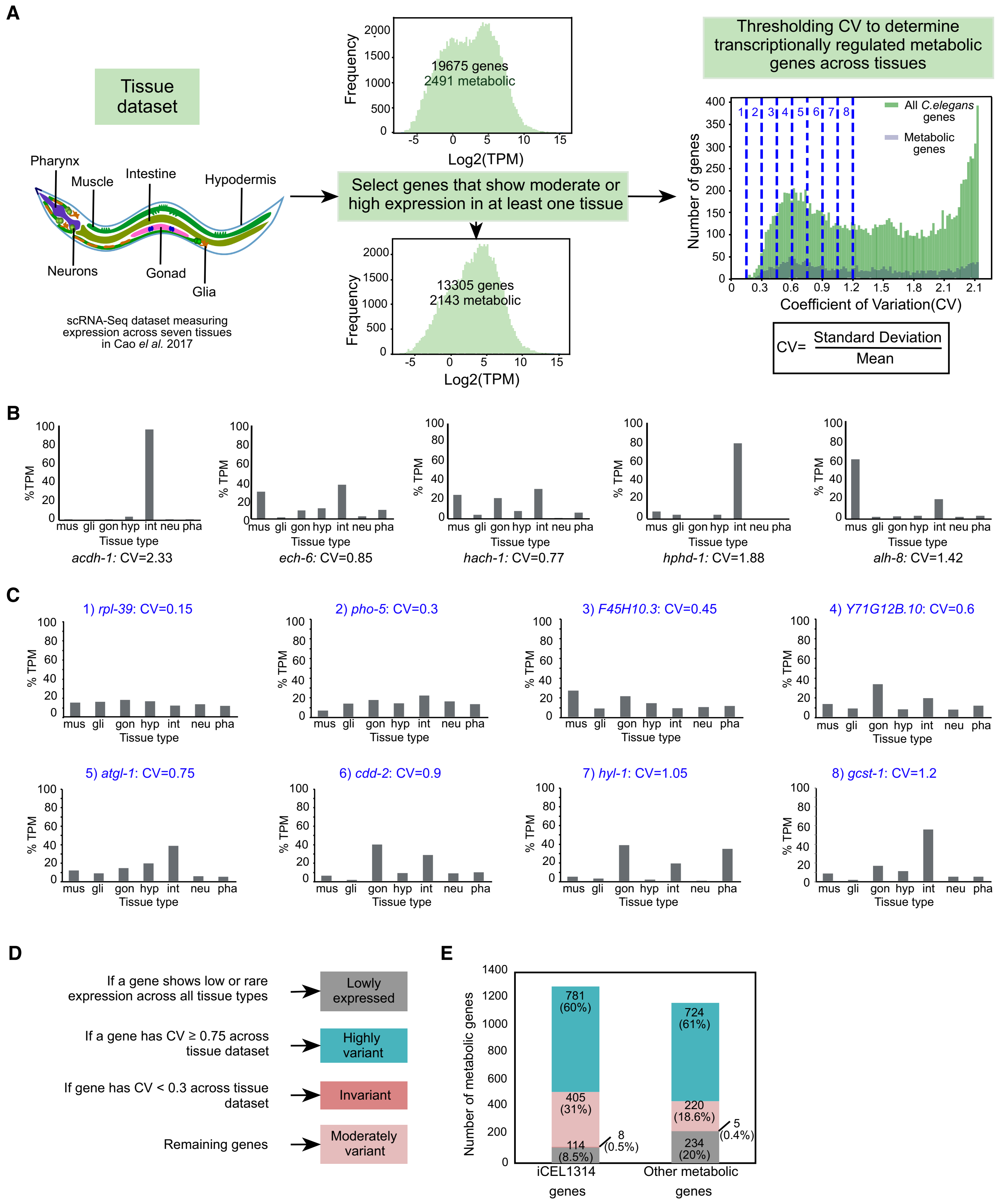

Figure EV2.

 © 2023 The Authors

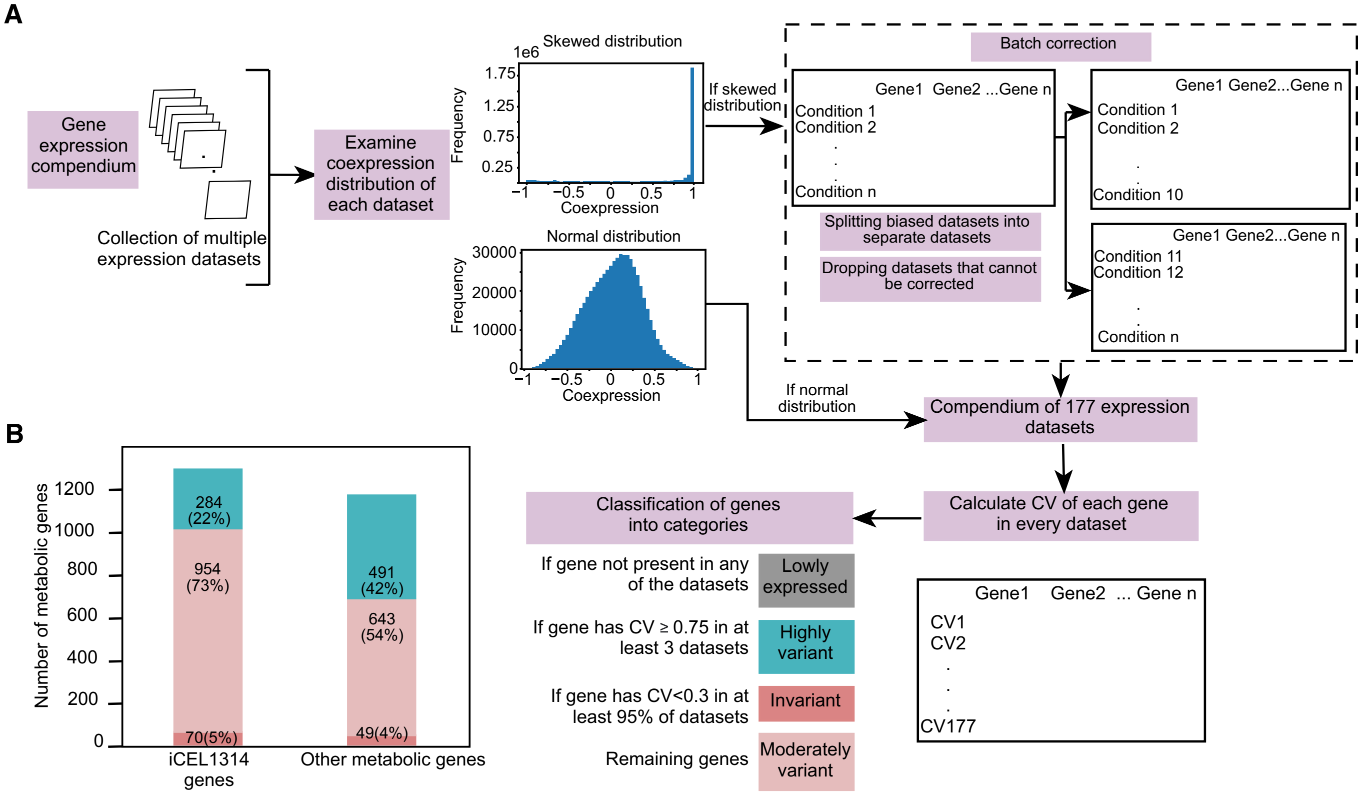

Figure EV3. Transcriptional regulation of metabolic genes across a compendium.

A Computational pipeline to determine transcriptionally regulated metabolic genes across the gene expression compendium. Datasets were evaluated for batch effects using correlation distribution. Some skewed datasets were corrected for batch effect by splitting into separate datasets, while some were removed if the source of skewness was not clear. This resulted in 177 datasets for analyses. CV of each gene was calculated. The criterion of classifying genes is based on the number of datasets with high CV and fraction of datasets with low CV.

B Bar graph showing low expressed, invariant, moderately variant and highly variant genes in the compendium in iCEL1314 and other metabolic genes. Color legend as indicated in (A).

© 2023 The Authors

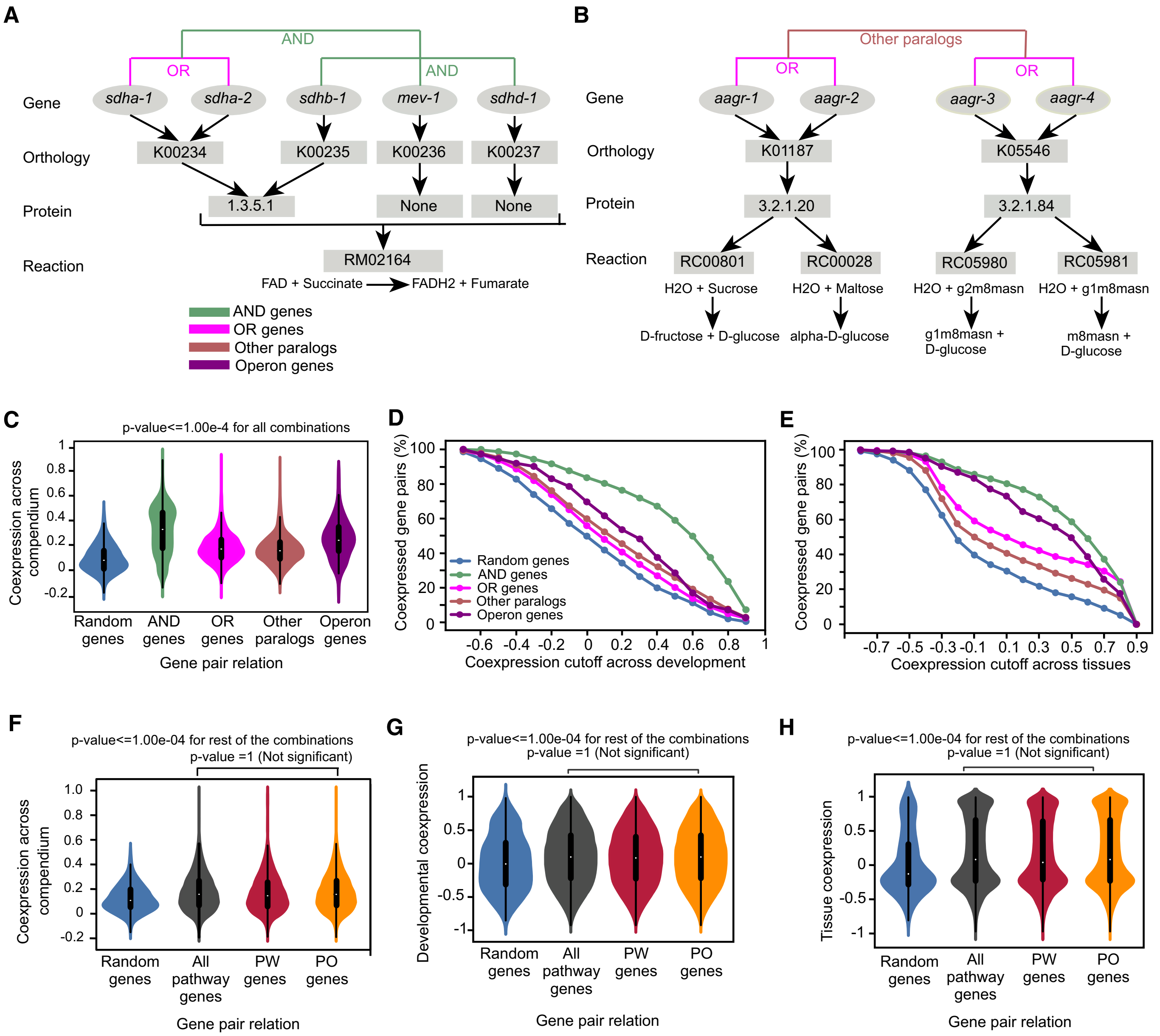

Figure EV4. Reaction-level analysis of metabolic pathways.

A The conversion of succinate to fumarate, which is part of complex II of the ETC and of the TCA cycle, is carried out by succinate dehydrogenase. Diagram showing that succinate dehydrogenase is composed of the OR genes *sdha-1* and *sdha-2* that each function together with the rest of the genes as AND genes. The GPR of this reaction is noted as [(*sdha-1* I *sdha-2*) & *sdhb-1*] & *mev-1* & *sdhd-1*]. Color legend is indicated.

B Example of a gene family (*aagr*) where members occur as paralogous OR gene pairs in separate reactions. Pairs of paralogs associated with different reactions are called other paralogs.

C Violin plot comparing coexpression for different populations of gene pairs including random, AND, OR, operon and other paralogs gene pairs in compendium of expression datasets.

D, E Percentages of AND, OR, other paralogs, operon genes and random metabolic gene pairs categorized as coexpressed using different coexpression values as cutoffs across developmental time (D) and tissues (E). Color legend as indicated in (D).

F–H Violin plots showing coexpression of random gene pairs, all pathway genes, PW genes and PO genes across compendium (F), different developmental stages (G) and tissues (H).

 © 2023 The Authors

Figure EV5. Semisupervised clustering of product matrix using relaxed parameters.

A Distinct cluster of known coregulated metabolic pathway propionate shunt genes together with isoleucine and valine degradation genes obtained using relaxed clustering parameters (deepSplit = 3, minClusterSize = 6) with the dynamic cut tree algorithm.

B Clusters of Met/SAM cycle genes and genes of related pathways obtained using relaxed (deepSplit = 3, minClusterSize = 6) and stringent (deepSplit = 2, minClusterSize = 3) parameters. Stringent clusters are shown near the drawn pathways of clustered genes.

C Pathway boundary (green shade) within selenocompound metabolism defined by genes in a high-quality cluster obtained by relaxed parameters and shown by the clustered heatmap (left). Mountain plots showing comparison of self-enrichment analysis statistics (NES, ES and FDR) of selenocompound metabolism from WormPaths with that of selenocompound cluster obtained by the semisupervised approach (right).

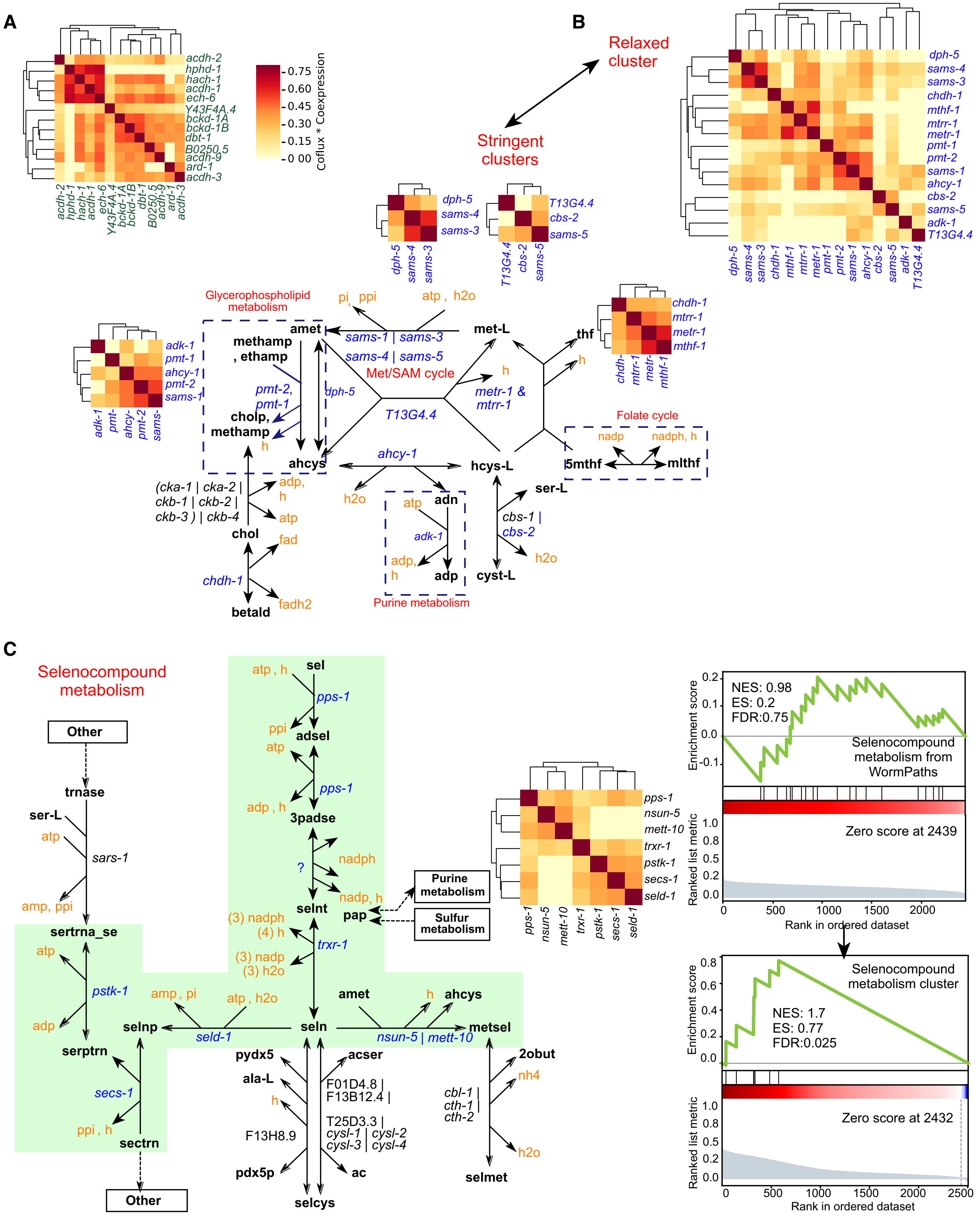

Figure EV5.

 © 2023 The Authors
