# Supplementary material for: Systems‐level transcriptional regulation of Caenorhabditis elegans metabolism
Source: Mol Syst Biol. 2023 Mar 21;19(5):e11443. doi: 10.15252/msb.202211443 (PMC10167481; doi:10.15252/msb.202211443)
Supplement: Supplementary file 14 — PDF+ [file MSB-19-e11443-s002.pdf]

Article

TRANSPARENT PROCESS OPEN ACCESS

molecular systems biology

Systems-level transcriptional regulation of *Caenorhabditis elegans* metabolism

Shivani Nanda[1], Marc-Antoine Jacques[1,†,‡], Wen Wang[2], Chad L Myers[2], L Safak Yilmaz[1] & Albertha JM Walhout[1,*]

Abstract

Metabolism is controlled to ensure organismal development and homeostasis. Several mechanisms regulate metabolism, including allosteric control and transcriptional regulation of metabolic enzymes and transporters. So far, metabolism regulation has mostly been described for individual genes and pathways, and the extent of transcriptional regulation of the entire metabolic network remains largely unknown. Here, we find that three-quarters of all metabolic genes are transcriptionally regulated in the nematode *Caenorhabditis elegans*. We find that many annotated metabolic pathways are coexpressed, and we use gene expression data and the iCEL1314 metabolic network model to define coregulated subpathways in an unbiased manner. Using a large gene expression compendium, we determine the conditions where subpathways exhibit strong coexpression. Finally, we develop "WormClust," a web application that enables a gene-by-gene query of genes to view their association with metabolic (sub)-pathways. Overall, this study sheds light on the ubiquity of transcriptional regulation of metabolism and provides a blueprint for similar studies in other organisms, including humans.

Keywords gene regulation; metabolic network model; metabolism; systems biology; transcription
Subject Categories Chromatin, Transcription & Genomics; Computational Biology; Metabolism
DOI 10.15252/msb.202211443 | Received 7 November 2022 | Revised 3 March 2023 | Accepted 7 March 2023 | Published online 21 March 2023
Mol Syst Biol. (2023) 19: e11443

Introduction

All organisms regulate their metabolism during development and to maintain homeostasis under fluctuating dietary and environmental conditions. In humans, failure to maintain homeostasis can lead to a variety of metabolic disorders such as inborn errors in human metabolism, obesity, hypertension, and diabetes (Sharma *et al*, 2008; DeBerardinis & Thompson, 2012). Metabolism can be regulated through different mechanisms. One well-known mechanism is allostery, a fast-acting mechanism where metabolites directly modulate enzyme activity. For instance, the enzyme phosphofructokinase, which regulates the conversion of fructose 6-phosphate to fructose 1,6-biphosphate, is allosterically regulated during glycolysis. This reaction is coupled to ATP hydrolysis where ATP binding to phosphofructokinase inhibits enzyme activity by decreasing its affinity for fructose 6-phosphate, while conversion to AMP reverses the inhibitory effect and increases the activity of the enzyme (Blangy *et al*, 1968; Schirmer & Evans, 1990). Metabolism can also be regulated transcriptionally by activating or repressing the expression of genes encoding metabolic enzymes or transporters. This mechanism is relatively slow and allows the organism to adapt to changing cellular or environmental conditions. Well-known examples of the transcriptional regulation of metabolism include induction of the lac operon in *Escherichia coli* in response to a switch from glucose to lactose as a carbon source (Jacob & Monod, 1961; Gilbert & Muller-Hill, 1966); the Leloir pathway in *Saccharomyces cerevisiae*, which is transcriptionally activated by galactose (Caputto *et al*, 1949; Hopper *et al*, 1978); and mammalian cholesterol biosynthesis genes, which are activated by the transcription factor (TF) SREBP when cholesterol levels are low (Brown & Goldstein, 1997; DeBose-Boyd & Ye, 2018). Another example of transcriptional rewiring of metabolism involves propionate degradation in the nematode *Caenorhabditis elegans*. Like humans, *C. elegans* utilizes a vitamin B12-dependent pathway to break down this short-chain fatty acid. When dietary vitamin B12 is low, propionate metabolism is transcriptionally rewired to an alternative degradation pathway referred to as the propionate shunt, thereby preventing toxic propionate accumulation (Watson *et al*, 2014, 2016; Bulcha *et al*, 2019).

The contribution of transcriptional regulation of metabolism has mostly been studied at a systems, or network, level, in single-cell organisms such as *E. coli* and *S. cerevisiae* and to a lesser extent in plants (Ihmels *et al*, 2004; Kharchenko *et al*, 2005; Seshasayee *et al*, 2009; Ledezma-Tejeida *et al*, 2017; Tang *et al*, 2021). However, the extent to which overall metabolic activity is under transcriptional control in animals remains unclear.

C. elegans is an excellent multicellular animal model to study the transcriptional regulation of metabolism at a systems level: Its fixed

1 Department of Systems Biology, University of Massachusetts Chan Medical School, Worcester, MA, USA
2 Department of Computer Science and Engineering, University of Minnesota, Minneapolis, MN, USA
*Corresponding author. Tel: +1 978 340 3072; E-mail: marian.walhout@umassmed.edu
†Present address: Cancer Research UK Cambridge Institute, University of Cambridge, Cambridge, UK
‡Present address: EMBL's European Bioinformatics Institute (EBI), Cambridge, UK

© 2023 The Authors. Published under the terms of the CC BY 4.0 license.

lineage of 959 somatic cells was fully described (Sulston & Horvitz, 1977), its metabolism shows extensive conservation with human metabolism (Lai *et al*, 2000; Shaye & Greenwald, 2011), many gene expression datasets are available, and a genome-scale metabolic network model (MNM) has been reconstructed (Yilmaz & Walhout, 2016). The most up-to-date MNM, iCEL1314, contains 907 metabolites, 2,230 reactions and 1,314 genes (Yilmaz *et al*, 2020). By using flux balance analysis (FBA), iCEL1314 can be used to gain insight into the metabolic state of *C. elegans* during different nutritional conditions or in different tissues. An additional set of metabolic genes has been predicted based on homology with known metabolic enzymes in other organisms or based on the presence of domains found in metabolic enzymes (Yilmaz & Walhout, 2016; Bhattacharya *et al*, 2022).

Guilt-by-association is a powerful concept in systems biology that can be used to identify genes with shared functions. One way this can be done is by coexpression analysis where a functional association can be predicted when genes are coexpressed in many transcriptomic datasets (Eisen *et al*, 1998; Hughes *et al*, 2000; Kim *et al*, 2001; Segal *et al*, 2003; Stuart *et al*, 2003). In *C. elegans*, coexpression analysis has been used to study global, temporal, and spatial gene expression (Reinke *et al*, 2000; Kim *et al*, 2001, 2016; Spencer *et al*, 2011; Liu *et al*, 2018).

Here, we investigated the extent of transcriptional regulation of *C. elegans* metabolism. We developed a computational pipeline to identify genes of which the corresponding mRNA varies significantly during development, in different tissues, and across a gene expression compendium consisting of different conditions. Using both a supervised and a semisupervised method, we identified coexpressed metabolic pathways and subpathways. Overall, we found that three-quarters of metabolic genes exhibit variation in expression, which is comparable to the proportion in nonmetabolic genes. Further, we found that most annotated metabolic pathways contain genes that are significantly coexpressed. With a custom-made semisupervised method, we identified clusters of genes that define coexpressed subpathways or combinations of subpathways that likely form functional metabolic units. We extracted conditions where coexpressed clusters of genes are coordinately activated or repressed, revealing how these clusters may contribute to metabolic homeostasis. We developed a web application we named "WormClust" that is available on WormFlux website (Yilmaz & Walhout, 2016). WormClust enables querying of *C. elegans* genes to identify metabolic (sub-)pathways with which these genes are coexpressed. Altogether, our findings show that transcriptional regulation of metabolic genes and pathways is ubiquitous in *C. elegans*, indicating that this principle is broadly conserved from single-cell organisms to metazoa. Finally, our analyses and tools provide a platform for similar studies in other organisms, including humans.

Results

Three-Quarter of metabolic genes are transcriptionally regulated

mRNA levels are determined by a combination of synthesis and degradation. Here, we used variation in mRNA levels as a first approximation for transcriptional regulation. We evaluated the expression of metabolic genes during development, in different tissues, and under different conditions to identify metabolic genes that are highly variant and therefore likely transcriptionally regulated. We used all annotated metabolic genes (Yilmaz & Walhout, 2016; Yilmaz *et al*, 2020) and grouped them into four classes based on current annotation (Dataset EV1): Class A, iCEL1314 genes ($N = 1{,}308$; after removal of six pseudogenes, see Materials and Methods); class B, genes annotated to reactions that cannot yet be connected to the iCEL1314 model ($N = 192$); class C, genes encoding proteins with homology to metabolic enzymes in other organisms ($N = 860$); and class D, genes encoding proteins with a domain found in known metabolic enzymes ($N = 132$). Hereafter, we refer to the 1,308 genes in class A as "iCEL1314 genes" and the remaining 1,184 as "other metabolic genes".

We first identified metabolic genes that vary in expression during larval development by using a high-quality postembryonic time-resolved RNA-seq dataset, hereafter referred to as the "development dataset" (Kim *et al*, 2013; Figs 1A and EV1A, and Dataset EV2). Briefly, this dataset contains expression profiles of stage-synchronized animals that were collected every 2 h after hatching for 48 h at 20°C. In the original paper, genes were grouped into 12 clusters based on similarity in developmental expression profiles. One of these clusters contains 5,045 genes, including 995 metabolic genes, with relatively invariant temporal expressions. We will refer to this cluster as the "flat cluster." However, although the expression levels of most of the flat cluster genes are relatively stable during development, we noticed that some did exhibit considerable variation. Additionally, many invariant genes from other clusters were not included in the flat cluster. Therefore, we used an unbiased statistical method, called variation score (VS) to stringently define variation in developmental gene expression. This included calculating deviation from the flat cluster genes' expression and then empirically establishing a conservative VS threshold (0.169; Fig EV1A and B, see details in Materials and Methods). We excluded 3,552 genes, including 213 metabolic genes, because they were expressed at levels too low for variability analysis. For the remaining metabolic genes, we found that 754 (31.4%, VS $\geq$ 0.169) are highly variant, and 98 were invariant (4%, VS = 0; Fig 1B). The remaining 1,332 metabolic genes (0 < VS <0.169) were annotated as moderately variant (Figs 1B and EV1C). About a quarter of iCEL1314 genes (329, or 26%) are highly variant, which is lower than the proportion of other metabolic genes (37%) and nonmetabolic genes (41%; Fig EV1C and D, and Dataset EV2). The percentage of highly variant metabolic genes is lower than that of nonmetabolic genes across most VS thresholds (Fig 1C).

To identify metabolic genes that exhibit differential expression across tissues, we selected a high-quality single-cell RNA sequencing dataset that measured gene expression during L2 stage of *C. elegans* across seven major tissues: body wall muscle, glia, gonad, hypodermis, intestine, neurons, and pharynx (Cao *et al*, 2017; Figs 1A and EV2A, and Dataset EV3). This dataset is hereafter referred to as the "tissue dataset." Unlike the development dataset, the tissue dataset does not have a defined cluster of invariant genes. Therefore, we used the less sophisticated coefficient of variation (CV) measure to identify variation in gene expression across the seven tissues (Fig EV2A). We previously found that the five genes comprising the propionate shunt are differentially expressed in different tissues (Watson *et al*, 2016; Yilmaz *et al*, 2020), and each of these genes had a CV greater than 0.75 (Fig EV2B). Visual inspection of genes with

 © 2023 The Authors

CV values from 0.15 to 1.2 indicates that CV = 0.75 provides a clear, yet conservative threshold to annotate highly variant genes across tissues (Fig EV2C). We further classified genes with a CV less than 0.75 but greater than or equal to 0.3 as moderately variant and genes with a CV less than 0.3 as invariant (Fig EV2D, see Fig EV2B and C for examples). A total of 6,370 genes, including 348 metabolic genes,

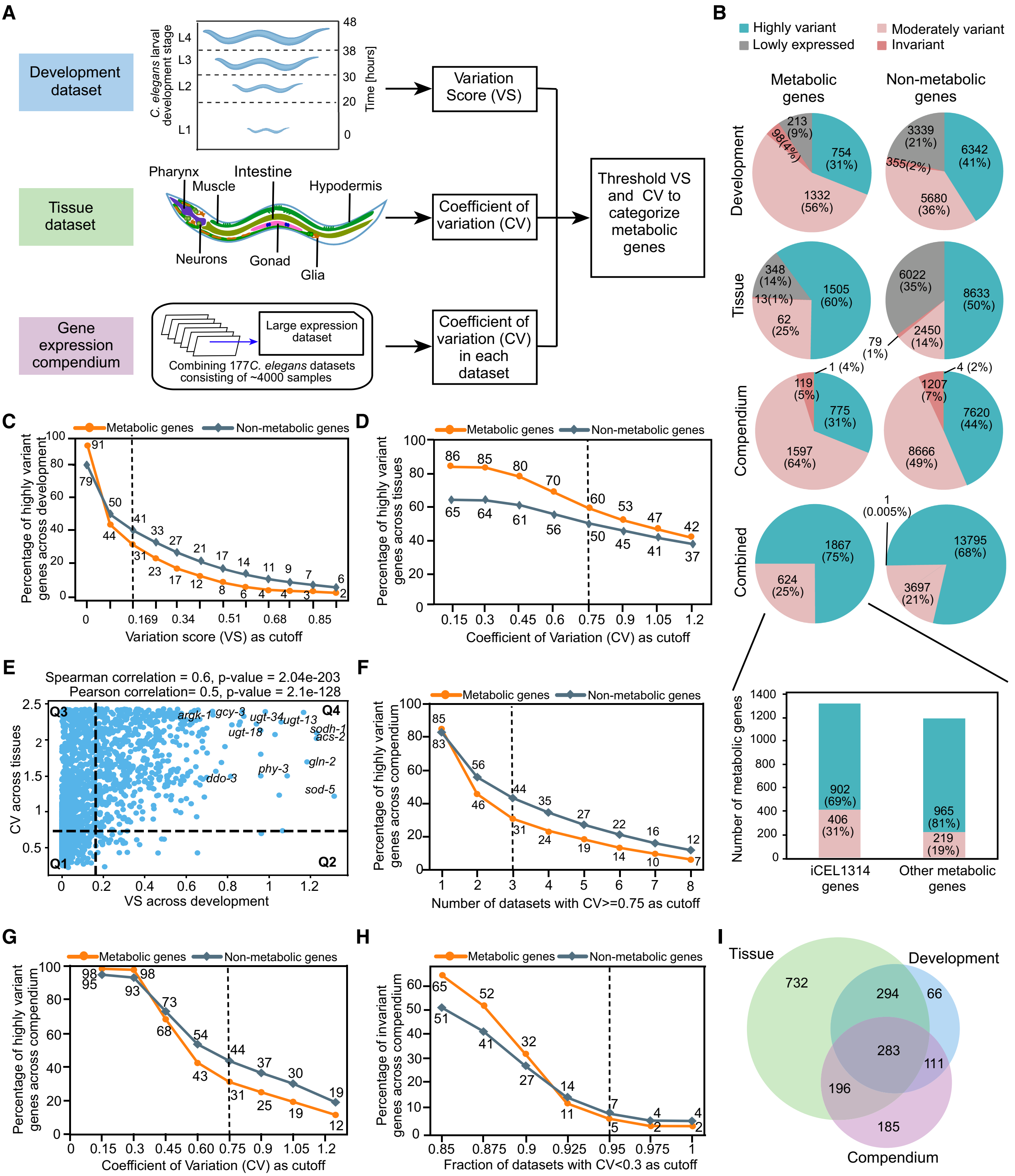

Figure 1.

◀ **Figure 1. Analysis of metabolic gene expression during development, in different tissues and in a gene expression compendium.**

A Computational pipeline to identify *C. elegans* metabolic genes that change in expression during development, across tissues, and compendium of multiple conditions. Statistically significant differences in gene expression were calculated in the developmental dataset using a variation score (VS), in the tissue dataset using coefficient of variation (CV) and by number of datasets with CV ≥ 0.75 in the compendium (collection of 177 datasets).

B Pie charts of metabolic and nonmetabolic gene expression variation in the three different datasets: development, tissue, and compendium separately and combined. Bar graph shows metabolic genes in iCEL1314 and other (predicted) metabolic genes.

C Comparison of percentage of highly variant metabolic versus nonmetabolic genes at different VS thresholds.

D Comparison of percentage of highly variant metabolic versus nonmetabolic genes at different CV thresholds.

E Scatter plot of VS (development) versus CV (tissue) of metabolic genes. The plot is divided into four quadrants: Q1 with moderate/low VS and moderate/low CV; Q2 with high VS and moderate/low CV; Q3 with moderate/ low VS and high CV; and Q4 with high VS and high CV. The Pearson and Spearman correlation coefficients and the corresponding *P*-values are indicated. Examples of Q4 genes that are highly variant both during development and in different tissues include *ugt-13*, *ugt-18*, and *ugt-34* (UGT enzymes); *gcy-3* (guanylate cyclases); and *ddo-3*, *gln-2*, *argk-1*, and *phy-3* (amino acid metabolism).

F Comparison of percentage of highly variant metabolic versus nonmetabolic genes at different cutoffs of number of datasets with high CV (≥ 0.75).

G Comparison of percentage of highly variant metabolic versus nonmetabolic genes at different CV cutoffs in at least three datasets in the compendium.

H Comparison of percentage of invariant metabolic versus nonmetabolic genes at different cutoffs of the fraction of datasets with low CV (< 0.3).

I Venn diagram of highly variant metabolic genes in the different datasets.

were not included in this analysis because they are expressed at low levels (Yilmaz *et al*, 2020; details in Materials and Methods). We identified ~60 and ~25% of metabolic genes as highly and moderately variant, respectively. These include 781 highly variant and 405 moderately variant iCEL1314 genes (Figs 1B and EV2E). A very small number of metabolic genes (13, or 1%) were invariant across tissues. Even though the analysis of the different datasets used a different statistical approach, these results suggest that more metabolic genes are variant and, therefore, likely transcriptionally regulated in different tissues than during development (Fig 1B). In contrast to development, the percentage of metabolic genes that are highly variant across tissues at any CV cutoff is greater than nonmetabolic genes (Fig 1D), indicating that metabolic processes exhibit a relatively high level of tissue specificity.

Overall, metabolic gene expression showed higher variation across tissues at a fixed time point (L2) than larval development. However, because we used two different statistical methods for the development and tissue datasets, we confirmed that it held true when we applied the same CV measure we used in the tissue dataset to the development dataset (Appendix Fig S1A). The two datasets also have different resolution: the development dataset has great temporal but no spatial resolution because it was measured by bulk RNA-seq while the tissue dataset, which was measured by single-cell RNA-seq, has great spatial but no temporal resolution. Therefore, we examined genes that are highly tissue-specific, because they are highly expressed in a single tissue in the tissue dataset, and found that only 57% of these are also highly variant in the development dataset (Appendix Fig S1B). Therefore, we conclude that transcriptional regulation of metabolic genes more frequently establishes spatial than temporal gene expression patterns.

To directly compare metabolic gene expression in tissues and development, we plotted VS values of metabolic genes across development versus CV values across tissues and found that these two parameters are moderately correlated (Fig 1E). We divided the scatter plot into four quadrants, based on the thresholds used in each dataset (Dataset EV4). To determine whether there are any functional enrichments, we performed pathway enrichment analysis (PEA) on the metabolic genes for each quadrant using the tool provided on the WormFlux website (Yilmaz & Walhout, 2016). The first quadrant (Q1) consists of genes with moderate/low developmental variation and moderate/low tissue variation. It has 595 metabolic genes, including 385 iCEL1314 genes that are enriched in several metabolic pathways, such as the electron transport chain (ETC), aminoacyl-tRNA biosynthesis, the tricarboxylic acid (TCA) cycle, the pentose phosphate pathway and glycolysis/gluconeogenesis (Appendix Fig S2A). The second quadrant (Q2), with high developmental variation and moderate/low tissue variation, consists of only 176 genes, including 68 iCEL1314 genes that are enriched in sulfur, cysteine, and methionine metabolism (Appendix Fig S2A). The 891 genes in the third quadrant (Q3) consist of genes with moderate/low developmental variation and high tissue variation. They include 504 iCEL1314 genes that are highly enriched in lipid metabolism. Notably, genes involved in peroxisomal fatty acid (FA) metabolism vary more in expression than mitochondrial FA degradation (Appendix Fig S2A). Finally, the 577 genes in the fourth quadrant (Q4) show high developmental variation and high tissue variation. They include 261 iCEL1314 genes, which are enriched in UDP-glucuronosyltransferases (UGT) enzymes, guanylate cyclases, glyoxylate and dicarboxylate metabolism, and amino acid metabolism, such as arginine and proline metabolism and glutamate/glutamine metabolism (Appendix Fig S2A). Interestingly, there are differences among different types of metabolic genes. For instance, amino acid metabolism genes are variant in both development and in tissues, while lipid metabolism genes are mostly variant in tissues, and growth and energy metabolism are relatively invariant in both development and in tissues.

To evaluate metabolic gene expression more broadly, we combined 177 expression profiling datasets into an expression compendium, an earlier version of which we have used to study TF paralogs (Reece-Hoyes *et al*, 2013; Figs 1A and EV3A, Datasets EV5 and EV6, see Materials and Methods). Using a CV threshold ≥ 0.75 in at least three datasets, we found that 775 of the 2,492 metabolic genes (~31%), including 284 iCEL1314 genes, are highly variant in the compendium, which is lower than nonmetabolic genes (44%, Figs 1B and EV3B). This difference holds true for different cutoffs of the number of datasets showing high variation (Fig 1F) and across different CV thresholds (Fig 1G). However, the percentage of invariant genes is similar between metabolic and nonmetabolic genes using different CV cutoffs (Fig 1H).

When we compared highly variant genes in development, tissue, and compendium, we found that a total of 1,867 metabolic genes (75%) are highly variant in at least one of the three datasets and that 283 metabolic genes are highly variant across all three datasets (Fig 1B and I). Using phenotypes provided in WormBase WS282

 © 2023 The Authors

(Harris *et al*, 2020b), we found that the 75% highly variant metabolic genes are enriched in conditional response variants such as chemical and pathogen response, and depleted in essential phenotypes such as lethality, larval arrest, slow growth, and sterility (Appendix Fig S2B). Finally, the remaining 624 (25%) of metabolic genes that are not highly variant in any dataset are similar in pathway enrichment as the Q1 genes discussed previously and are enriched in the essential phenotypes (Appendix Fig S2C and D).

Altogether, our analyses indicate that at least 75% (1,867 out of 2,492) of metabolic genes vary in mRNA levels and are therefore likely transcriptionally regulated, including 902 iCEL1314 genes (~69%; Fig 1B). This is similar to the proportion of varying nonmetabolic genes (~79%), indicating that metabolic genes are overall at least as much under transcriptional control as other genes (Fig 1B).

A supervised approach shows widespread Coexpression of genes comprising metabolic pathways

We previously found that the five genes comprising the propionate shunt pathway are coordinately activated in response to propionate accumulation (Watson *et al*, 2016; Bulcha *et al*, 2019). In addition, we found strong coexpression of genes functioning in the methionine/S-adenosylmethionine (Met/SAM) cycle, for instance when flux through this pathway is perturbed (Giese *et al*, 2020). To systematically test which *C. elegans* metabolic pathways exhibit coexpression, we developed a custom pathway enrichment analysis pipeline (Fig 2A) based on gene set enrichment analysis (GSEA; see Materials and Methods; Subramanian *et al*, 2005) and applied it to the compendium. We ran this pipeline using metabolic pathways, enzyme complexes, and enzyme families as defined in WormPaths (Walker *et al*, 2021). Henceforth, we use "category" to refer to a group of metabolic genes that best fit in an enzyme complex or related set of enzymes such as aminoacyl-tRNA synthetases. We calculated an enrichment score (ES) that defines the enrichment of relatively high coexpression within that set. A normalized ES (NES) indicates relative strength of this enrichment compared with randomized tests, the significance of which is measured as a false discovery rate (FDR; Fig 2A). With an FDR cutoff of ≤ 0.05, 52 of 84 metabolic pathways or categories (~61%) exhibit coexpression, which is significantly more than expected by chance (Fig 2B and C, Dataset EV7, Appendix Fig S3). As expected, the 52 coexpressed metabolic pathways and categories include the propionate shunt and the Met/SAM cycle (Fig 2D and E). When we examined coexpression in pathways and categories separately, we found that 78% of categories showed significant coexpression compared to 58% of pathways (Fig 2B). Examples of metabolic pathways that exhibit high coexpression include peroxisomal FA degradation and starch and sucrose metabolism (Fig 2F and G). Examples of coexpressed categories include vacuolar ATPases, ETC complex I, and aminoacyl-tRNA synthetases (Appendix Fig S3). There are 32 categories and pathways that do not exhibit self-enrichment, including pantothenate and CoA biosynthesis and mevalonate metabolism (Fig 2H and I, and Appendix Fig S3). Such pathways may either not be regulated at all, may be regulated by allostery, or only one or a few genes in these pathways are transcriptionally regulated and may therefore function as key regulatory genes. Alternatively, these pathways maybe coregulated in conditions that were not yet profiled and therefore are not included in the compendium.

The extent of within-pathway coexpression of metabolic genes can be potentially confounded because metabolic reactions are often associated with multiple genes in gene-protein-reaction (GPR) annotations (Kim *et al*, 2008; Thiele & Palsson, 2010). There are two reasons for this. First, some metabolic reactions are catalyzed by enzyme complexes comprising two or more proteins. In such cases, all genes need to be expressed for the reaction to take place and are therefore annotated here as "AND" genes. Second, some genes are part of larger families (paralogs) that encode isozymes or highly similar proteins. Metabolic network reconstruction efforts use protein sequence homology to associate genes with reactions (Thiele & Palsson, 2010; Yilmaz & Walhout, 2016, 2017). As a result, multiple highly homologous paralogs may be associated with the same metabolic reaction. Such paralogs are annotated here as "OR" genes. Some reactions are associated with a combination of AND and OR genes (Fig EV4A). Finally, for some gene families it may be that one member catalyzes one reaction and another member catalyzes another. Paralogs that are associated with distinct reactions are referred to here as "other paralogs" (Fig EV4B). Pathways can be associated with multiple types of AND and OR genes (Fig 2J).

AND genes encode proteins that function together in complexes, and such genes are often strongly coexpressed (Jansen *et al*, 2002). For example, genes encoding ETC complex members are coexpressed and coregulated (van Waveren & Moraes, 2008). Therefore, we wondered whether this holds true for AND genes in iCEL1314 and, if so, whether this would inflate pathway coexpression enrichment. To test this, we systematically assessed coexpression of different types of gene pairs. As expected, we found that AND genes are significantly more coexpressed than random gene pairs, OR genes and other paralogs (Figs 2K and EV4C–E). Both OR genes and other paralogs are also more coexpressed than random metabolic gene pairs in all three datasets (Figs 2K and EV4C–E). Surprisingly, OR genes are more coexpressed than other paralogs across tissues (Figs 2K and EV4C–E).

In *C. elegans*, ~18% of genes are transcribed from operons (Blumenthal *et al*, 2002). In total, 26% of metabolic genes occur in operons. However, they most frequently occur as a pair with a nonmetabolic gene. In total, 242 metabolic genes (~10% of all metabolic genes) occur in a pair with another metabolic gene in an operon (Dataset EV1).

As expected, these operon gene pairs are more coexpressed than random gene pairs, OR genes and other paralogs, thus serving as a validation for our coexpression analysis. However, these pairs are less coexpressed than AND genes and there is no overlap between the two categories. This shows that enzyme complexes are strongly coregulated and their coregulation mechanism is largely independent of operonic organization (Figs 2K and EV4C–E).

Based on the analysis of AND, OR and operon genes, it is difficult to determine the contribution of the coexpression of such gene pairs to pathway enrichment. Therefore, we examined coexpression of gene pairs that are annotated with distinct reactions in a pathway, which we refer to as pathway (PW) genes (Fig 2J). We found that PW gene pairs are significantly more coexpressed than random gene pairs (Figs 2L and EV4F–H). We also examined coexpression of gene pairs that are not part of an operon, which we refer to as pathway excluding operon (PO) genes. There are only three pathway gene pairs that are part of operon; hence, there is no significant difference between pathway genes and PO genes coexpression (Figs 2L and

© 2023 The Authors

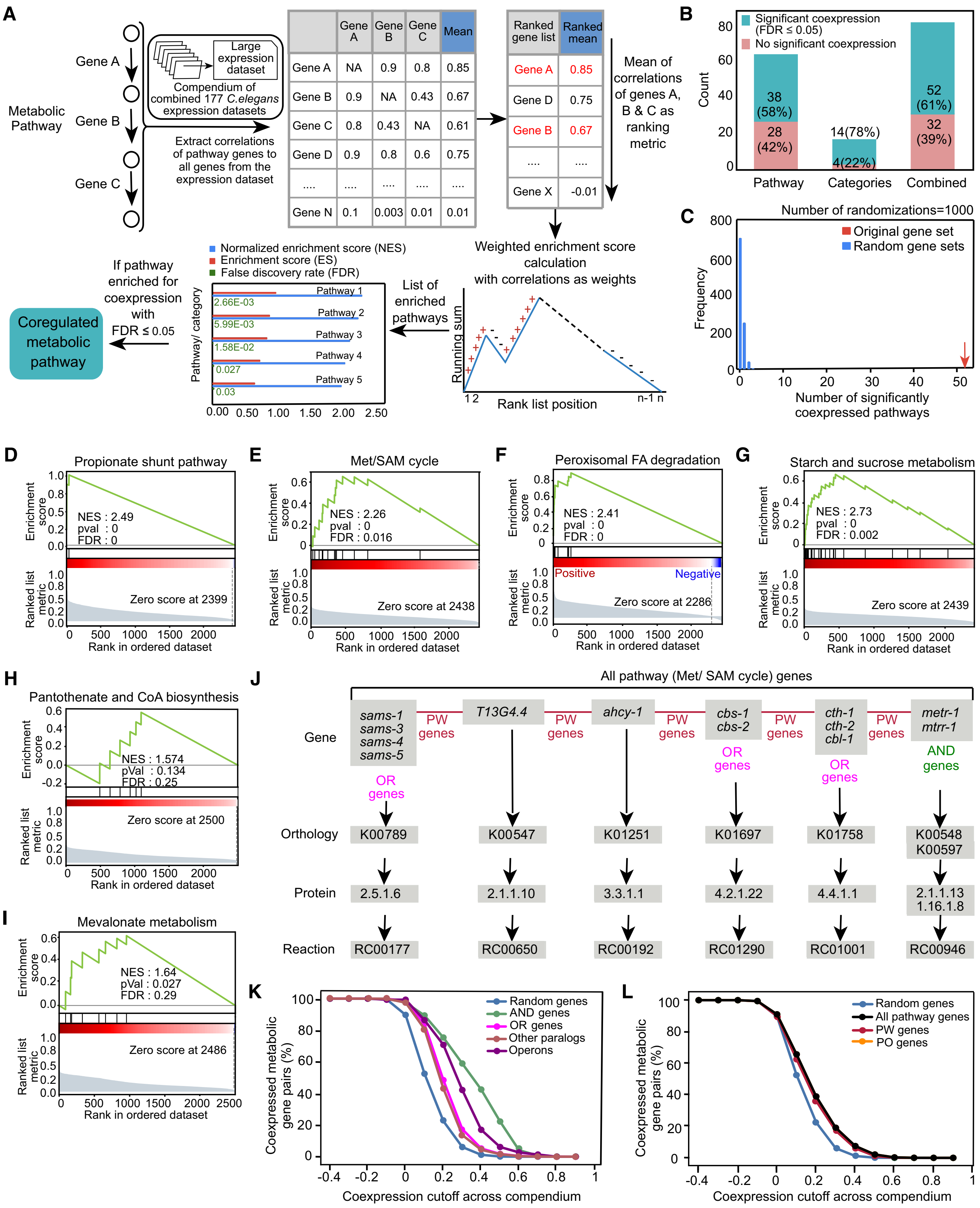

Figure 2.

© 2023 The Authors

Figure 2. Supervised approach to investigate coexpression of metabolic pathways.

A Custom computational pathway enrichment analysis pipeline that determines coexpressed genes functioning in the same metabolic pathway. Pairwise coexpression was based on the gene expression compendium. For every annotated metabolic pathway, the coexpression of pathway genes (columns) to all metabolic genes (rows) was extracted. A ranked list of genes was obtained for each pathway by taking the mean of coexpression values in rows while ignoring self-correlations. Weighted gene set enrichment analysis was then performed to find significantly enriched pathways. If a pathway is self-enriched with FDR $\leq$ 0.05, it is annotated as coexpressed.

B Bar graph indicating the percentage of metabolic pathways and categories that show significant coexpression compared with ones that are not self-enriched for coexpression.

C Histogram denoting the number of significantly coexpressed metabolic pathways obtained by 1,000 randomizations while maintaining the structure of the data.

D–I Mountain plots showing self-enrichment of (D) propionate shunt pathway, (E) Met/SAM cycle, (F) peroxisomal fatty acid degradation pathway, (G) starch and sucrose metabolism, (H) pantothenate and CoA biosynthesis, and (I) mevalonate metabolism.

J Metabolic pathways often consist of reactions catalyzed by single genes, OR genes and AND genes. All genes involved in the same pathway are collectively annotated as all pathway genes. Genes that are associated with distinct reactions are annotated as PW genes. PW gene pairs exclude AND and OR gene pairs. Met/SAM cycle pathway, which consists of 13 metabolic genes, is shown as an example.

K Percentages of pairs of AND genes, OR genes, other paralogs, operon genes, and random metabolic genes categorized as coexpressed using different coexpression values as cutoffs. Coexpression values are based on the gene expression compendium.

L Percentage of random, all pathway, PW, and PO gene pairs categorized as coexpressed using different coexpression values as cutoffs. Coexpression values are based on the gene expression compendium.

EV4F–H). Therefore, pathway coexpression is not just driven by AND, OR and operon genes, indicating that pathway genes' coexpression is a true feature of many metabolic pathways.

A Semisupervised approach extracts coexpressed subpathways

Our finding that metabolic pathways and categories exhibit extensive coexpression was based on previously annotated pathways (Walker *et al*, 2021). However, these pathways connect into the larger metabolic network and the definition of the start and ending of each pathway is somewhat arbitrary. Since there is extensive coexpression of genes that function together in predefined pathways, we reasoned that we may be able to use coexpression to extract metabolic (sub)-pathways in an unbiased manner. To specifically focus on metabolic genes that function in connected reactions in the metabolic network, we developed a "coflux" metric that calculates flux dependency between metabolic genes using the network model (see details in Materials and Methods; Dataset EV8). Reactions in linear pathways have complete flux dependence (i.e., coflux = 1), while in branched pathways flux dependency may be partial (coflux = between 0 and 1), and in uncoupled reactions, there is no dependence (coflux = 0). We then used a custom semisupervised approach that multiplies coflux and coexpression values and clustered the resulting product matrix with a relatively stringent set of parameters (Fig 3A and B, Dataset EV9, Appendix Fig S4A, see Materials and Methods for details).

As expected, the propionate shunt pathway genes formed a tight cluster (Fig 3C and Appendix Fig S4B). Interestingly, while the first four genes, *acdh-1*, *ech-6*, *hach-1*, and *hphd-1*, occurred closely together, the fifth gene, *alh-8*, was not part of the same cluster. This could be explained in two ways. First, *alh-8* encodes an enzyme that functions at a junction in the pathway where its substrate malonate semialdehyde is converted either to acetyl-coa or, potentially, to beta-alanine. Therefore, metabolic flux is divided in two directions and is not linearly coupled with the shunt pathway flux like the first four reactions. Second, *alh-8* is annotated to another reaction where 2-methyl-3-oxopropionate is converted to propionyl-CoA (Fig 3C). This approach also revealed another cluster comprising the canonical, vitamin B12-dependent propionate degradation pathway, indicating that, like the propionate shunt, this pathway may also be transcriptionally activated or repressed under specific conditions (Fig 3C, Dataset EV9).

The semisupervised approach reveals pathway boundaries

The propionate shunt example above shows that the semisupervised approach can extract subpathways (e.g., the propionate shunt) from previously defined pathways (e.g., propionate degradation) based on coexpression and coflux. We therefore used other clusters defined by the semisupervised approach to better define starts and ends of different pathways.

An example of a pre-annotated WormPaths pathway that was fully captured with the semisupervised approach is peroxisomal FA degradation (Fig 4A, Dataset EV9). The first reaction in peroxisomal beta-oxidation is catalyzed by acyl-CoA oxidases (encoded by *acox* genes). Only *acox-1.1* and *acox-3* in the *acox* family genes are coexpressed with the other peroxisomal FA oxidation genes, indicating that they are more likely to function in this pathway than the other *acox* genes, which are coexpressed with each other, and with mitochondrial FA degradation genes (Dataset EV9).

Examples where only a subset of annotated pathway genes clustered together include tyrosine metabolism and histidine degradation. Tyrosine can be metabolized via different reactions, in different pathway branches (Fig 4B). In one pathway branch, tyrosine is degraded in five steps to produce fumarate and acetoacetate. The genes in this branch; *gst-43*, *C31H2.4*, *Y53G8B.1*, *hpd-1*, *hgo-1*, *fah-1*, *and gst-42* form a tight cluster (Fig 4B, Dataset EV9). This cluster consists of OR genes *hpd-1* OR C31H2.4; and Y53G8B.1 OR *gst-42* OR *gst-43*, which suggests that these genes are correctly annotated to this pathway branch. Histidine can also be degraded via two pathway branches: one converting histidine to glutamate through N-formyl-L-glutamate and the other converting histidine to 3-methylimidazoleacetic acid. The four genes associated with the conversion of histidine to N-formyl-L-glutamate; *haly-1*, *Y51H4A.7*, *amdh-1*, *and cpin-1*, form one of the top-ranked clusters (Fig 4C, Dataset EV9). However, the genes in the other branch are not coexpressed.

We also found clusters consisting of genes that traversed different pathways. For instance, *alh-8* and *gta-1*, which are functionally associated but not strongly coexpressed with the propionate shunt

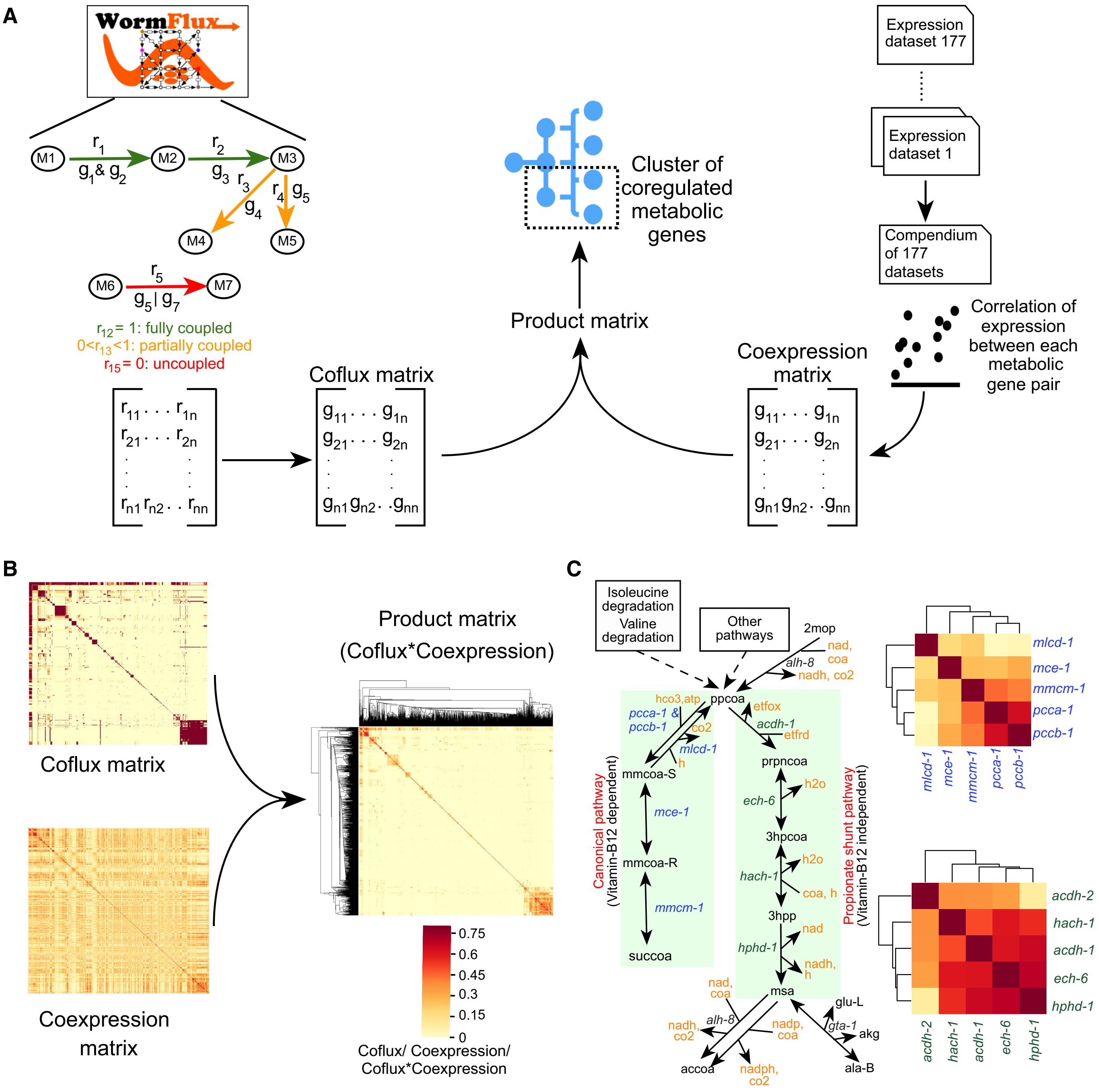

Figure 3. Semisupervised approach to extract coexpressed and flux-dependent metabolic genes.

A Computational pipeline to extract tightly coregulated units in the metabolic network: Functional relationships are provided through theoretical flux associations (coflux) calculated using *C. elegans* metabolic network model iCEL1314, while expression correlations come from the compendium of 177 expression datasets. Hierarchical clustering on the product of coflux and coexpression matrix gives coexpressed metabolic pathways.

B Heatmaps showing coflux and coexpression of iCEL1314 genes and clustered heatmap showing added modularity to coexpression space by product of coexpression and coflux. Color legend is indicated.

C Distinct clusters denoted by clustered heatmap of genes in canonical and shunt pathways of propionate degradation were extracted using dynamic cut tree algorithm with stringent parameters (deepSplit = 2, minClusterSize = 3). Color legend as indicated in (B).

(Fig 3D), cluster with T09B4.8 (alanine metabolism) and four other genes belonging to pyrimidine metabolism: *dpyd-1*, *dhp-1*, *dhp-2* and *upb-1* (Fig 4D, Dataset EV9). This observation functionally connects genes in what were heretofore separately annotated pathways, that is, pyrimidine, alanine, and propionate metabolism. The coexpression of these genes suggests that thymine is degraded,

 © 2023 The Authors

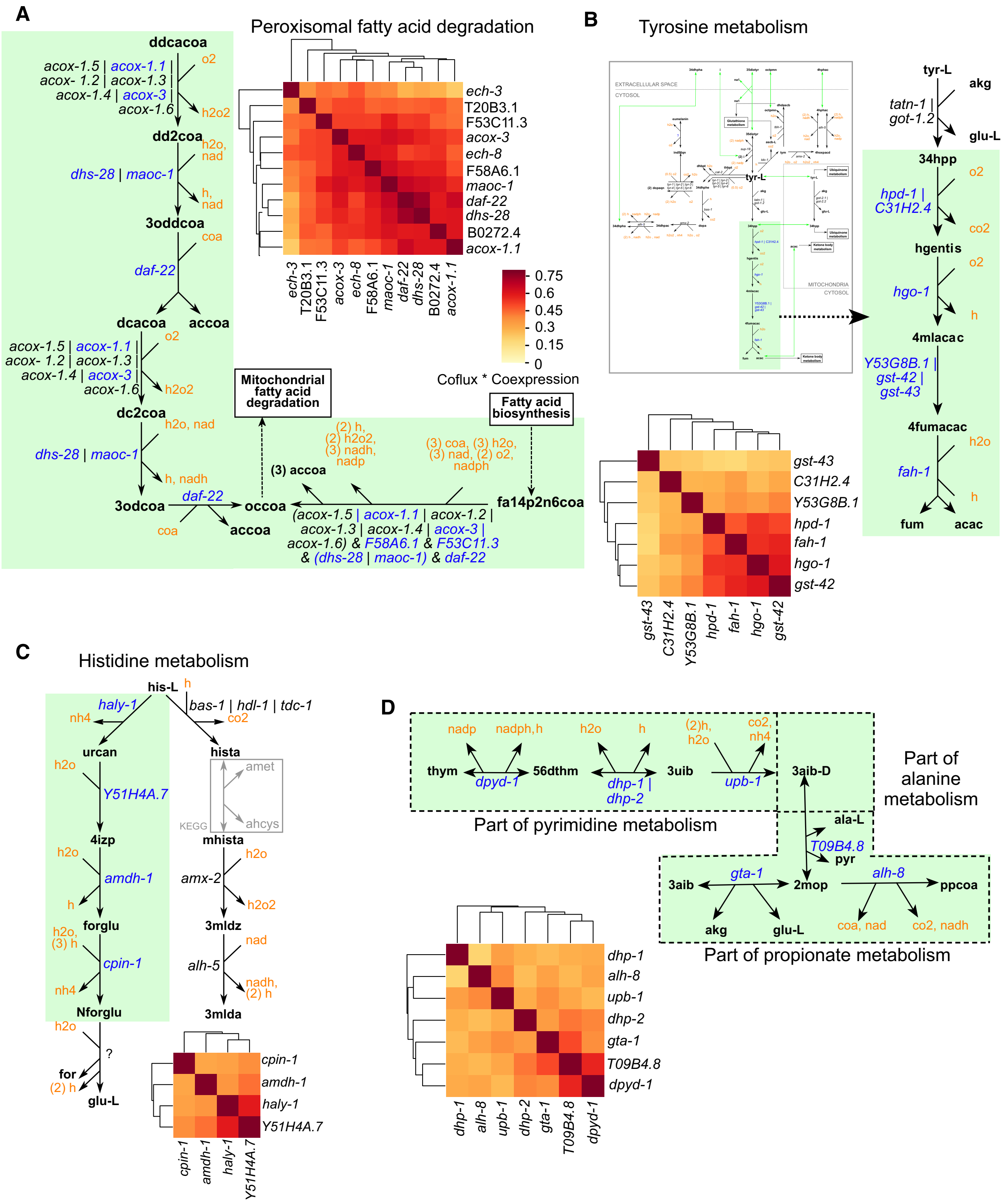

Figure 4. Semisupervised approach defines metabolic pathway boundaries.

A Clustered heatmap denoting a distinct cluster consisting of at least one gene from every reaction in peroxisomal fatty acid degradation. Heatmap genes are shown in bold font. Color legend, indicated here, applies to all panels.
B Clustered heatmap showing a distinct cluster formed by the tyrosine degradation genes separate from the rest of the tyrosine metabolism.
C Clustered heatmap showing a distinct cluster formed by a boundary within the histidine degradation pathway.
D Clustered heatmap showing a distinct cluster formed by genes traversing pathway boundaries that are parts of propionate, alanine, and pyrimidine metabolism.

leading to the formation of propionyl-CoA, L-3-amino-isobutanoate and acetyl-CoA. This observation also suggests that *alh-8* levels have a stronger functional role in the conversion of 2-methyloxopropanoate to propionyl-CoA than in the propionate shunt. Remarkably, this further indicates that *alh-8* may participate in both the generation and degradation of propionyl-CoA.

The stringent cluster derivation parameters we used favor small clusters, and as a result, interconnections between different pathways may be lost in the analysis. To unveil such connections, we relaxed the parameters to allow the derivation of larger clusters of coexpressed genes (Dataset EV10, Appendix Fig S4C). With these settings, the propionate shunt cluster expanded and included *bckd-1A*, *bckd-1B*, *dbt-1*, *Y43F4A.4*, *ard-1*, *acdh-3*, *acdh-9*, and *B0250.5*, which are annotated to branched-chain amino acids (BCAA) isoleucine and valine degradation pathways, but not *alh-8* or *gta-1* (Fig EV5A, Appendix Fig S4D, Dataset EV10). Propionyl-CoA, the starting metabolite of the propionate shunt, is produced by the breakdown of valine and isoleucine. We recently proposed that the propionate shunt not only functions to detoxify excess propionate but also to produce acetyl-CoA for ketone body and energy production (Ponomarova *et al*, 2022). The coexpression of valine and isoleucine breakdown genes with the propionate shunt indicates a functional connection between these pathways to produce energy.

The Met/SAM cycle provides another example of different degrees of clustering that can be unveiled with different parameter settings (Fig EV5B). The smaller clusters with stringent clustering captured different parts of one-carbon metabolism with their connections to Met/SAM cycle, while relaxed clustering combined these genes into one single cluster. This cluster acts as a subsystem that connects the Met/SAM cycle on one side with glycerophospholipid metabolism, specifically phosphatidylcholine biosynthesis, which depends on methylation reactions using SAM (Walker *et al*, 2011), as well as with purine metabolism (Ducker & Rabinowitz, 2017). Second, Met/SAM cycle genes are highly coexpressed with the folate cycle gene which produces the methyl group that is used to convert homocysteine into methionine in the Met/SAM cycle (Ducker & Rabinowitz, 2017; Giese *et al*, 2020). Together, these results confirm that the Met/SAM cycle is overall coexpressed (Giese *et al*, 2020) and show that additional co-functioning genes can be identified.

The semisupervised approach also identified gene clusters that are not part of any coexpressed pathway identified by the supervised method above. An example is selenocompound metabolism, where a set of seven genes form a highly coexpressed cluster (FDR = 0.025, NES = 1.7; Fig EV5C). In comparison, the respective FDR and NES values for self-enrichment of the WormPaths pathway of selenocompound metabolism were 0.75 and 0.98 (Dataset EV7). Altogether, these results illustrate that not all genes in a pathway are coexpressed and further indicate that a subset of a pathway or a combination of subsets from multiple pathways may be under transcriptional control, illustrating the utility of semisupervised approach as an addition to the predefined metabolic pathways in WormPaths.

Metabolic pathway communities reveal coexpression among complexes and pathways

To explore additional coexpression clusters than those that were captured by the relaxed settings described previously, we visually inspected the product matrix and extracted three clusters we refer to as metabolic pathway "communities" (Fig 5A). We analyzed these communities by WormPaths PEA (Walker *et al*, 2021). The first community is enriched in ETC complexes I, III, and IV, indicating broad transcriptional control of energy production (Fig 5B, Dataset EV11). The second community is enriched in mitochondrial and peroxisomal FA degradation, FA biosynthesis, ascaroside biosynthesis, and BCAA degradation (Fig 5C, Dataset EV11). The connection between FA metabolism and BCAA degradation may reflect the fact that some FAs are synthesized from BCAA breakdown products. For instance, branched-chain fatty acids (BCFAs) are synthesized from the branched-chain alpha-keto acids of valine, leucine, and isoleucine such as isovaleryl-CoA and isobutyryl-CoA after their decarboxylation and further chain elongation (Daschner *et al*, 2001; Jia *et al*, 2016; Wallace *et al*, 2018). The third community is enriched in aminoacyl-tRNA biosynthesis, N-glycan biosynthesis, collagen biosynthesis, iron metabolism, and mevalonate metabolism, all of which produce biomass precursors. While aminoacyl-tRNA synthetases play a major role in protein biosynthesis by linking amino acids to their cognate transfer RNAs (tRNAs), mevalonate metabolism provides precursors for glycan, collagen biosynthesis provides collagen for the formation of the cuticle and other extracellular matrices, and iron metabolism is important for many aspects of metabolism, including the production of heme groups of heme proteins. This result points toward the possibility that growth is transcriptionally regulated by a central mechanism controlling pathways that produce biomass precursors and assemble biomass (Fig 5D, Dataset EV11). Taken together, we confirmed the coexpression of metabolic pathways and revealed coexpressed subpathways, as well as coexpression among pathways.

Metabolic subpathways are activated or repressed under different conditions

The gene expression compendium is comprised of 177 expression profiling datasets that measure relative mRNA levels in a variety of experimental conditions and genotypes. Therefore, we next asked whether we could identify specific conditions in which different metabolic gene clusters are activated or repressed. Using a custom computational pipeline (Fig 6A), we first identified those datasets that best represent the coexpression of a particular cluster. We then manually investigated the top datasets for each cluster (see Materials and Methods). To validate this approach, we first examined the expression of propionate shunt cluster genes in top-scoring datasets. We previously showed that propionate shunt genes are repressed in animals fed *Comamonas aquatica* DA1877, a bacterium that (unlike the standard *E. coli* OP50 diet) produces vitamin B12, thus enabling flux through the canonical propionate degradation pathway (MacNeil *et al*, 2013; Watson *et al*, 2013, 2014, 2016). The dataset from that study, labeled as dataset 15 in the compendium, scored as most significant for propionate shunt gene coexpression, where the genes are expressed in animals fed *E. coli* OP50, but not in animals fed *C. aquatica* (Fig 6B, Dataset EV4). Interestingly, propionate shunt genes are also highly coexpressed in a dataset that measured expression in *spr-5* mutants versus wild-type animals across 1(f1), 13(f13) and 26(f26) generations (Fig 6B, dataset 139). Propionate shunt genes are more highly expressed in the N2 reference strain compared with *spr-5* mutant animals (Fig 6B). Since *spr-5* encodes a

 © 2023 The Authors

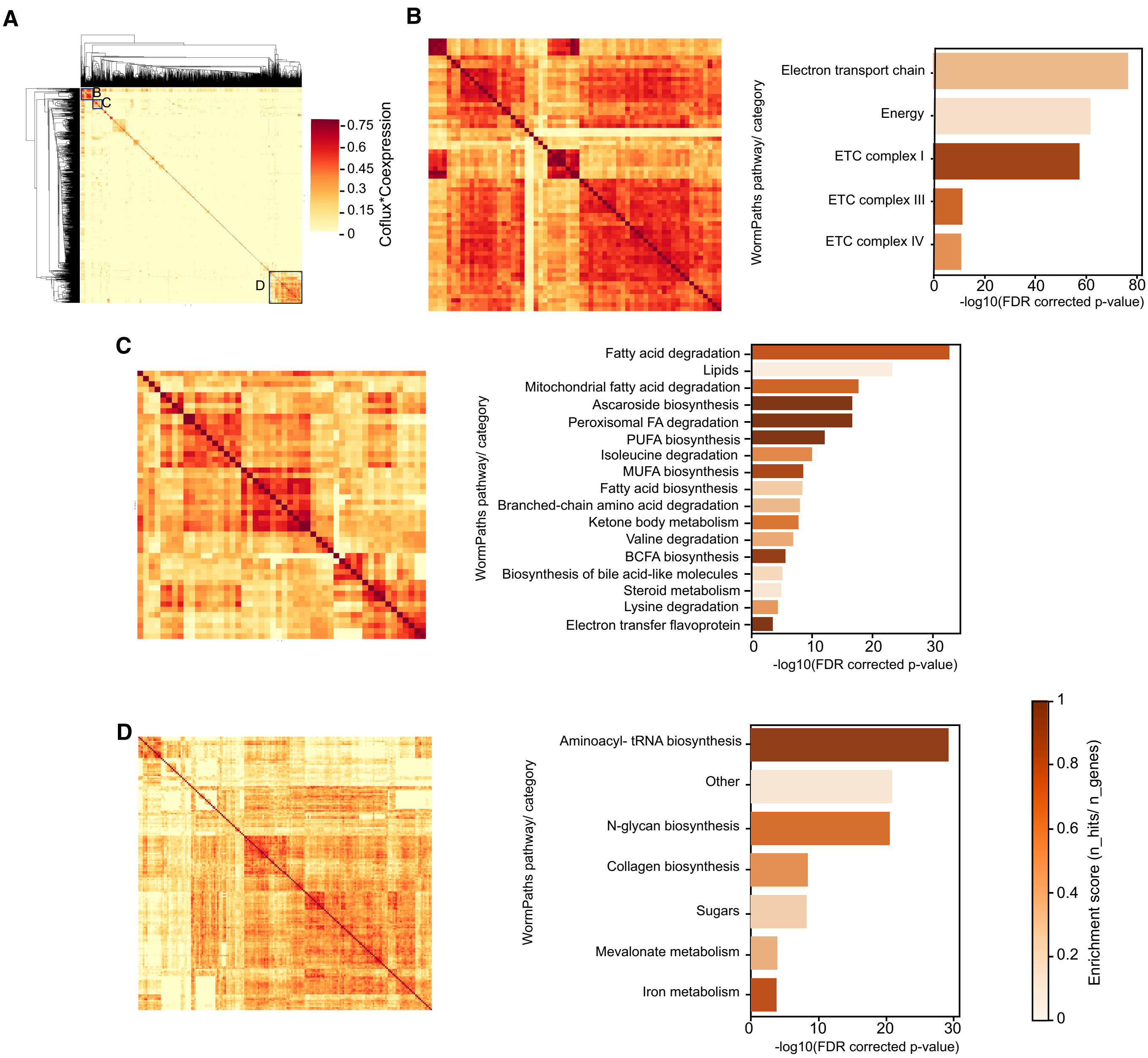

Figure 5. Extraction of metabolic communities.

A Clustered heatmap indicating communities formed by multiplying coflux and coexpression values of iCEL1314 genes. (B, C, and D) define three major communities shown in the respective parts of this fig.

B–D PEA of communities B (B), C (C), and D (D).

histone demethylase, this may indicate that the expression of shunt genes is regulated not only by TFs but also by epigenetic mechanisms.

Peroxisomal FA degradation genes were most significantly coexpressed in dataset 132, which measured gene expression in precisely staged embryos during the first quarter of embryonic development (Fig 6C). The time course included a stage of exclusively maternal transcripts (four-cell), the transition to zygotic transcription (28-cell), and the presumptive commitment to the major cell fates (55-, 95-, and 190-cell stages; Yanai & Hunter, 2009). Peroxisomal FA degradation genes were lowly expressed in four-cell embryos and their expression increased in later embryonic stages, which may reflect a change in carbon source for energy and biomass generation prior to hatching and feeding. Peroxisomal FA degradation genes are also upregulated in animals fed *P. aeruginosa* compared with the standard *E. coli* OP50 diet (Fig 6C, dataset 51). This may reflect the high energy demand during infection (Nhan *et al*, 2019).

© 2023 The Authors

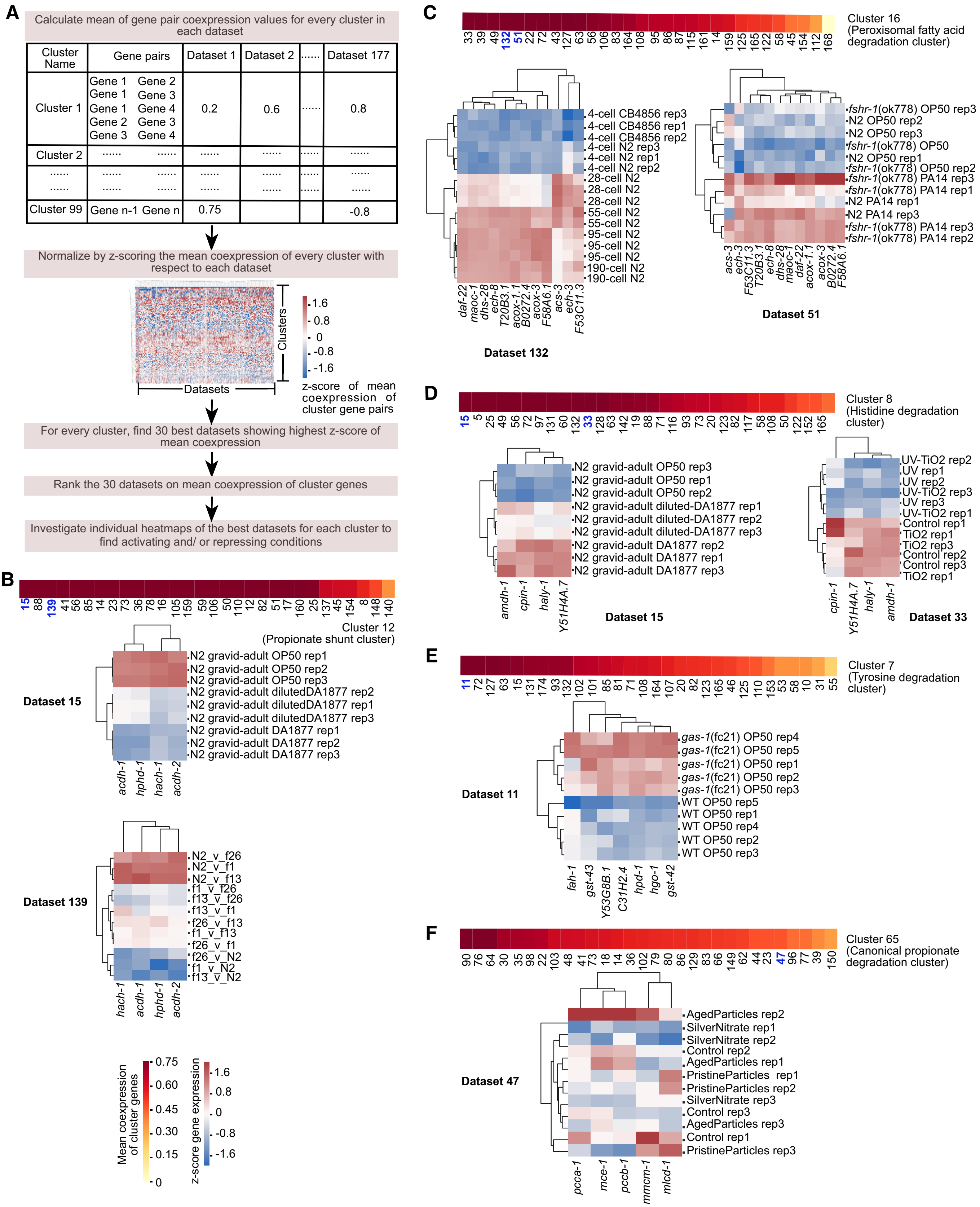

Figure 6.

 © 2023 The Authors

Figure 6. Condition analysis of metabolic gene coexpression.

A Computational pipeline to extract activating or repressing conditions of metabolic clusters. The mean coexpression of all gene pairs in each cluster in each dataset is calculated separately. To rank datasets that showed highest coexpression uniquely for each cluster, these mean coexpression values are normalized using z-scoring across each dataset (as shown by heatmap). Thirty best datasets that potentially represent activation/repression conditions of each cluster are identified by the z-score values of mean coexpression. Then, for each cluster, datasets are manually inspected in the order of decreasing mean coexpression along with its associated published paper and the heatmap to understand activation/repression conditions.

B–F Mean coexpression of the 30 best datasets for clusters of propionate shunt (B), peroxisomal fatty acid degradation (C), histidine degradation (D), tyrosine degradation (E), and canonical propionate degradation (F), followed by heatmap examples from selected datasets as indicated by bold-blue dataset numbers. Color bar and heat map legend as indicated in (B).

Inspection of expression of the histidine degradation cluster discussed above revealed that it is highly expressed in animals fed *C. aquatica* (Fig 6D, dataset 15). However, these genes were not affected in animals fed *E. coli* supplemented with vitamin B12 (Bulcha *et al*, 2019), suggesting that the effect of *C. aquatica* on this cluster may be independent of this cofactor. Animals showed lower expression of this cluster upon exposure to UV treatment (Fig 6D, dataset 33). In humans, UV converts cis-uruconate (the product of first reaction of histidine degradation, Fig 4C) to trans-uruconate, which has been proposed to play a protective role in skin (Brosnan & Brosnan, 2020). Our result indicates that, in *C. elegans*, UV exposure rewires metabolic flux to avoid histidine degradation, for instance to preserve histidine that could be converted to trans-uruconate.

The tyrosine degradation cluster was most significantly coexpressed in dataset 11, wherein expression profiles of *gas-1* mutant animals, which are deficient in mitochondrial respiration, were compared with wild-type animals (Falk *et al*, 2008; Fig 6E). This study revealed that free tyrosine levels are decreased in *gas-1* mutants. In addition, there is a failure of NAD^+-dependent ketoacid oxidation in mitochondrial respiratory chain mutants (Falk *et al*, 2008). To compensate for this respiratory dysfunction, multiple pathways are upregulated, including the TCA cycle and ketone body metabolism (Falk *et al*, 2008). Since the end products of tyrosine degradation pathway cluster are the TCA cycle intermediate fumarate and the ketone body acetoacetate (Fig 4B), the function of the upregulation of tyrosine degradation during mitochondrial dysfunction may be to supply metabolites for compensatory pathways.

Altogether, these results show that the gene expression compendium can be used to gain insight into the conditions that most greatly affect the activation or repression of different metabolic gene clusters. However, one needs to be careful to manually inspect the conditions of interest because sometimes coexpression can be biased by an outlier experiment. An example of this is dataset 47, one of the top datasets in which canonical propionate degradation pathway genes are coexpressed. Even though the conditions in this dataset are not related to canonical propionate breakdown, this dataset falsely appears as one of the top datasets due to coexpression driven by one bad outlier sample. It is however also possible that this outlier sample was unknowingly contaminated to change the nutritional or environmental state, hence driving the variable expression of these genes (Fig 6F).

Overall, our systematic analysis revealed specific conditions of when metabolic gene clusters are activated or repressed, reinforcing our overall finding that transcriptional regulation plays an important role in the control of metabolism.

WormClust web application enables gene-by-gene query to identify coexpression with metabolic (sub)-pathways

A major premise of this study is the assumption that variance in mRNA levels results, at least in part, from transcriptional regulation, which in turn suggests that genes are coexpressed because they are coregulated. In reverse engineering of gene regulatory networks, coexpression of TFs with their target genes has been used to define causal relationships (Segal *et al*, 2004; MacNeil & Walhout, 2011). To make our data available to the community as well as to enable the easy identification of TFs and other *C. elegans* genes that are coexpressed with metabolic (sub)-pathways, we developed a web application named WormClust, which is available on the WormFlux website (Yilmaz & Walhout, 2016). This tool takes any *C. elegans* gene as input and evaluates its coexpression with metabolic (sub)-pathways. If the query gene is an iCEL1314 gene, the output is a clustered heatmap of the coexpressed genes in the model based on product matrix, and according to the selected level of stringency, that is, relaxed, or stringent. If the query gene is not an iCEL1314 gene, then an association of the gene with annotated metabolic pathways is provided. The threshold for this association can be based on FDR and/or NES (Fig 7A).

We, and others, previously found that nuclear hormone receptor (NHRs) TFs frequently associate with metabolic genes in different types of assays and dataset (Van Gilst *et al*, 2005; Arda *et al*, 2010; Mori *et al*, 2017; Bhattacharya *et al*, 2022). To illustrate the utility of WormClust, we tested three NHRs with known metabolic pathway associations for coexpression with annotated metabolic pathways in the compendium of 177 *C. elegans* expression datasets. All of these showed coexpression with their target metabolic pathways (Fig 7B–D): *nhr-68* was highly coexpressed with the propionate shunt (FDR = 0.02 and NES = 2.1; Bulcha *et al*, 2019), *nhr-31* associated with vacuolar ATPases (FDR = 0.036, NES = 2.04; Hahn-Windgassen & Van Gilst, 2009), and *nhr-79* is coexpressed with peroxisomal FA degradation (FDR = 0.019, NES = 2.3; Zeng *et al*, 2021). In addition to pathways, we also performed enrichment of clusters or subpathways from our semisupervised analysis with TFs. We found that *nhr-79* is enriched to cluster 16 (stringent), which contains peroxisomal FA degradation genes; *nhr-31* shows enrichment to cluster 5 (relaxed) that consists of vacuolar ATPases; and *nhr-68* shows enrichment to cluster 12 (stringent) which contains propionate shunt genes, albeit with higher FDR. In addition, *nhr-68* shows enrichment to cluster 40 consisting of *mans-2*, *hex-2*, and *fut-8* (N-glycan biosynthesis), and cluster 51 consisting of *bgal-1*, *gana-1* (galactose metabolism) and *hex-1* (sphingolipid metabolism; Appendix Fig S5A–C, Dataset EV9). This observation suggests

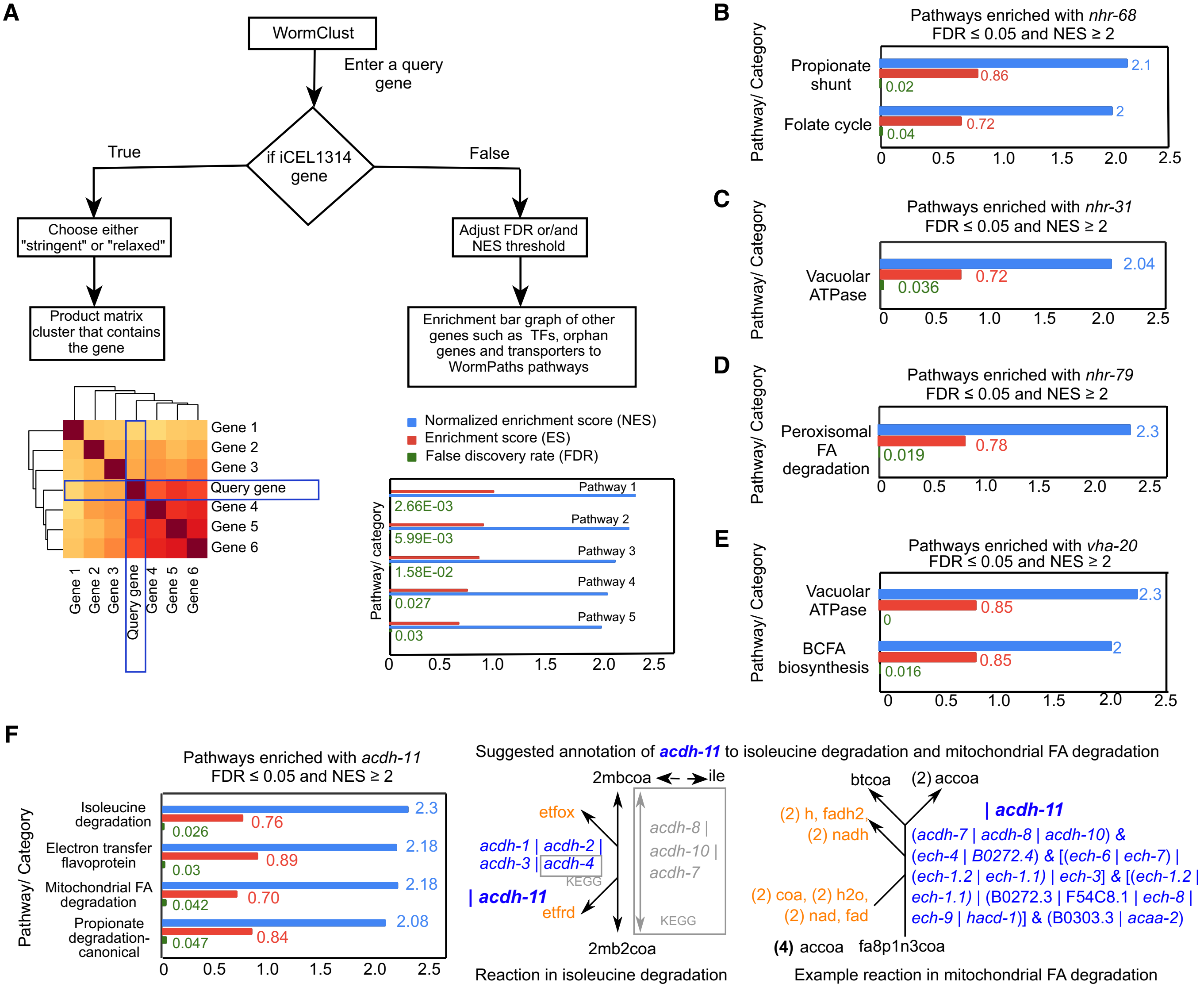

Figure 7. WormClust: a web application that enables querying of genes to identify coexpression with metabolic (sub)-pathways.

A Diagram showing the workflow of WormClust. A *C. elegans* gene is taken as input. If the gene is part of iCEL1314, a clustered heatmap of closely associated genes in product matrix of coflux and coexpression is displayed, based on stringency level of clustering. If the input gene is not an iCEL1314 gene, enrichment bar graphs of the gene to annotated metabolic pathways are displayed, based on selected FDR and NES thresholds.

B Bar graph of pathways that are significantly coexpressed with *nhr-68* with NES ≥ 2 and FDR ≤ 0.05.

C Plot showing significant coexpression of *nhr-31* with vacuolar ATPases (FDR ≤ 0.05 and NES ≥ 2).

D Plot showing significant coexpression of *nhr-79* with peroxisomal fatty acid degradation (FDR ≤ 0.05 and NES ≥ 2).

E Plot showing significant coexpression of *vha-20* with vacuolar ATPases (FDR ≤ 0.05 and NES ≥ 2).

F Bar graph of pathways that are significantly coexpressed with *acdh-11* with NES ≥ 2 and FDR ≤ 0.05 (left). Examples of specific reactions in metabolic network model where *acdh-11* can be annotated as OR gene (right).

that *nhr-68* may play a broader role in the regulation of metabolic gene expression.

WormClust also provides an opportunity to annotate new metabolic genes. For example, *vha-20*, a vacuolar ATPase that is not part of the original iCEL model because it was only recently annotated by WormBase and KEGG, shows significant enrichment to vacuolar ATPases (Fig 7E). This result suggests that WormClust can be used to "deorphan" unannotated metabolic genes. As another example, we found that *acdh-11* is highly coexpressed with mitochondrial fatty acid degradation and isoleucine degradation genes (Fig 7F). Therefore, we propose that *acdh-11* can now be added to the iCEL model as another OR gene to reactions in these pathways. We envision future systematic studies of orphan metabolic genes and TFs to increase the annotation of metabolic genes and elucidate the transcriptional mechanisms that regulate their expression.

 © 2023 The Authors

Discussion

In this study, we performed a systems-level analysis of mRNA level variation as a proxy for the transcriptional regulation in *C. elegans*. Even with our conservative approach, we found that most metabolic genes are under transcriptional control. By a combination of supervised and semisupervised methods, we found that genes in many metabolic pathways are coexpressed and identified gene clusters that represent parts of pathways, or combinations of pathways with strong coexpression. These results build upon earlier work in single-cell organisms such as *E. coli* and *S. cerevisiae* (Ihmels *et al*, 2004; Kharchenko *et al*, 2005; Seshasayee *et al*, 2009; Ledezma-Tejeida *et al*, 2017; Tang *et al*, 2021) implying that coexpression of metabolic genes that function together is a principle that is evolutionarily conserved.

Our data indicate metabolic genes are more subject to transcriptional regulation across tissues than during development. What is the purpose of transcriptional activation or repression of metabolic genes? We propose that the transcriptional regulation of metabolic genes can serve different purposes. First, there is extensive transcriptional regulation of metabolic genes in different tissues. This can be viewed as the setup of metabolic network functionality depending on a tissue's needs. Indeed, tissues have different needs. For instance, the *C. elegans* intestine serves as the entry point of bacterial nutrients and requires the expression of enzymes that aid digestion and metabolite transport to other tissues. Similarly, the animal's muscle needs to produce energy to support movement and is therefore highly catabolic. Second, metabolic genes are transcriptionally regulated during development. This likely reflects the need for different aspects of metabolism as tissues differentiate and grow and as different metabolic functions are necessary. We refer to the expression of different metabolic genes and pathways in different tissues and different developmental stages as "metabolic network wiring." A third function of metabolic gene activation or repression is under different conditions that dictate the need for different metabolic functions. This can be for the breakdown of different nutrients, for example, carbohydrates versus fats, versus protein, or to rewire metabolic pathways when others are perturbed. An example of this is the propionate shunt, which is transcriptionally activated when flux through the preferred, vitamin B12-dependent pathway is perturbed (Watson *et al*, 2016; Bulcha *et al*, 2019). We refer to the transcriptional rerouting of metabolism as "metabolic rewiring." Finally, metabolic genes can be transcriptionally activated when flux through the pathway in which they function is hampered. An example of this is the Met/SAM cycle in *C. elegans*, which is transcriptionally activated when flux through the cycle is low, for instance under low vitamin B12 dietary conditions (Giese *et al*, 2020). For genes encoding enzymes that function in multiple reactions and metabolic pathways (e.g., *alh-8*), we can learn with which pathways they are more strongly coexpressed. Our study provides a facile portal to investigate the tissues, developmental stages, or conditions under which particular metabolic genes and pathways are highly coexpressed, which will help to formulate hypotheses for detailed follow-up studies.

Genes that are coexpressed often function together and are frequently coexpressed with their transcriptional regulators (Eisen *et al*, 1998; Hughes *et al*, 2000; Kim *et al*, 2001; Segal *et al*, 2003; Stuart *et al*, 2003). We used this principle to develop WormClust with which any *C. elegans* gene, including TFs, can be used to search for metabolic pathways with which it is coexpressed. However, it is important to note that the most critical regulators, those that respond to the initial information, are often not coexpressed with their target genes. Indeed, *nhr-10*, which is essential for activation of the propionate shunt in response to high levels of propionate, does not change much in expression under relevant conditions (Bulcha *et al*, 2019). To identify such "first responders," it will be useful to employ promoter-reporter strains with large-scale RNAi screens (MacNeil *et al*, 2015; Bhattacharya *et al*, 2022). Further, at least half of all *C. elegans* metabolic genes are not yet associated with reactions or pathways (Yilmaz *et al*, 2020). We have already shown the utility of WormClust in "deorphaning" genes and connecting them to metabolic network such as in case of *vha-20* and *acdh-11* (Fig 7E and F). We propose that more such genes may be "deorphaned" in the future. Longer term, we envision that association of other types of regulators, such as RNA binding proteins and microRNAs, with metabolic pathways can be used to gain broader insights into the functional connections among different biological processes.

Finally, the approaches used here should be broadly applicable to any organism, including humans, for which large gene expression profile compendia and high-quality metabolic network models are available. By applying these approaches, deeper insights into the transcriptional control of metabolism will be obtained, as well as insights into the conditions under which metabolic genes and pathways are activated or repressed.

Materials and Methods

Reagents and Tools table

Reagent/ Resource	Reference or Source	Identifier or Catalog Number
**Software**		
Python 3.6.6	https://www.python.org	N/A
MATLAB 2019a	https://www.mathworks.com	N/A
GSEApy 0.1.18	https://doi.org/10.1093/bioinformatics/btac757	N/A
dynamicTreeCut 0.1.0	https://CRAN.R-project.org/package=dynamicTreeCut (Langfelder *et al*, 2008)	N/A

© 2023 The Authors

Reagents and Tools table (continued)

Reagent/ Resource	Reference or Source	Identifier or Catalog Number
Numpy 1.18.3	Harris *et al* (2020a)	N/A
Pandas 1.1.0	https://pandas.pydata.org/	N/A
Matplotlib 3.2.1	https://matplotlib.org/	N/A
Scikit-learn 0.20.3	Pedregosa *et al* (2011)	N/A
Scipy 1.4.1	Virtanen *et al* (2020)	N/A
Seaborn 0.9	https://joss.theoj.org/papers/10.21105/joss.03021	N/A
Statannot 0.2.3	https://doi.org/10.5281/zenodo.7213391	N/A
Sleipnir	Huttenhower *et al* (2008)	N/A

Methods and Protocols

Preprocessing of genes

The master list of *C. elegans* genes considered for analysis was downloaded from WormBase public ftp site (release WS282; Harris *et al*, 2020b). The genes were filtered out to obtain only live and protein-coding genes, which amounted to 19,985 genes in total.

Development dataset

Postembryonic expression profiles were based on published RNA-seq data (Kim *et al*, 2013). Briefly, the authors measured the transcriptome of wild-type (N2) animals from hatching to 48-h post-hatching every 2 h. This dataset includes 21,714 protein-coding genes, including 2,405 metabolic genes. Genes were classified into 12 clusters based on their expression profiles (Kim *et al*, 2013). We refer to the cluster showing relatively invariant expression as the "flat cluster" and the genes within this cluster as "flat genes." Selecting only live protein-coding genes (WS282) resulted in a total of 18,113 genes, including 2,397 metabolic genes. Of these, 4,689 are flat genes, including 995 metabolic genes. We generated a histogram of average gene expression across development using the logarithm of reads per kilobase per transcript (RPKM) values at base 2. This resulted in a bimodal expression distribution that was fitted by two superposed Gaussian curves, representing a high-expression subpopulation and a low-expression subpopulation (LES). Genes that showed expression values less than the mean plus the standard deviation of LES at all the time points were filtered out to avoid false fluctuations in gene expression. After this step, a total of 14,561 genes were left, including 2,184 metabolic genes. The number of flat genes was reduced to 4,646, including 986 metabolic genes.

Tissue dataset

Tissue-level expression profiles were based on a single-cell RNA-seq dataset of animals at the second larval stage (L2; Cao *et al*, 2017). This dataset provides gene expression as transcripts per million (TPM) for 20,271 protein-coding genes including 2,506 metabolic genes across seven major tissues: body wall muscle, glia, gonad, hypodermis, intestine, neurons, and pharynx. Selecting only live genes (WS282) reduced the number of genes to 19,675, including 2,491 metabolic genes. The dataset was previously processed to label gene expression in every tissue according to the level of expression into four categories: high, moderate, low, and rare (Yilmaz *et al*, 2020). Genes that showed rare or low expression in all seven tissues were filtered out in this study, resulting in 13,305 genes including 2,143 metabolic genes.

Gene expression compendium

A compendium of gene expression datasets was generated using a combination of public datasets. First, 374 microarray, RNA-Seq, and tiling array datasets related to *C. elegans* were downloaded from WormBase (Harris *et al*, 2020b). Then, only those datasets that consisted of at least 10 conditions were selected, resulting in 169 datasets. These datasets were individually examined for batch effects, since many were obtained from multiple microarray experiments where total RNA was not normalized. Initially, histograms of expression values were analyzed, and 16 microarray datasets that displayed abnormal distributions where the correlation distributions were skewed toward +1, hence suggesting that the data may consist of samples that are highly distinctive from each other or are from separate experiments altogether. Such datasets were selected for further examination (Fig EV3A). Twelve of these datasets were found to be composed of two subsets of data, where all genes in one subset were up or downregulated with respect to the other except for a few. The samples forming each subset were independent of the other and seemed to have different amounts of total RNA or a similar batch effect. Therefore, these datasets were divided into two separate datasets to correct for batch effects. The remaining four datasets were removed since the source of abnormalities in their distributions of expression was not clear. This processing resulted in a total of 177 datasets. For each dataset, the expression of every gene was normalized by converting the expression values to z-scores based on expression across all conditions using the Normalizer function of Sleipnir library (Huttenhower *et al*, 2008). Once all the datasets were z-normalized, they were combined to form a compendium with 4,796 conditions (sum of multiple conditions within 177 datasets) using the Combiner function of the Sleipnir library (Huttenhower *et al*, 2008), which took a union set of all genes across the

 © 2023 The Authors

different datasets and converted missing values to NaN (not a number) for subsequent processes.

Calculation of variation score in the development dataset

We define variation score (VS) as a measure of the deviation of a gene's expression profile from a flat reference over time in the development dataset (Kim *et al*, 2013). Prior to any analysis, expression values of every gene were normalized by total expression in all time points using equation (1),

$$x_i^{norm} = \frac{x_i}{\sum_1^i x_i}, \quad (1)$$

where x_i indicates the expression value of gene x at time i. To define a reference profile of invariant expression, a line was constructed in time by joining the mean normalized expression value of the flat cluster at every time point. An envelope around this line was then defined by adding and subtracting the standard deviation of each point. A deviation from this envelope, referred as variation score (VS), was then computed by taking the average distance between an individual gene profile and the flat reference profile according to equation (2),

$$VS_g = \frac{\sum d_i}{n}$$
$$with \begin{cases} d_i \\ \quad = 0, if\ x_i^{norm} \in \mu_i \pm \sigma_i \\ else\ min\left(\left|x_i^{norm} - (\mu_i + \sigma_i)\right|, \left|x_i^{norm} - (\mu_i - \sigma_i)\right|\right) \end{cases} \quad (2)$$

where n is the number of observations for the gene g, d_i is the distance at time i between the normalized level of expression of the gene x_i^{norm} and the closest border of the reference flat profile, and μ_i and σ_i are mean and standard deviations of normalized expression values of flat genes at time point i, respectively. A graphical example of this calculation is provided in Fig EV1A. With this definition, a VS = 0 means that the profile of a given gene stays within the envelope of the flat cluster and is therefore perfectly flat, or invariant. To define highly variable genes, we empirically established a conservative VS threshold value of 0.169 based on the distribution of VS between flat genes and all other genes, such that 97% of flat genes were not annotated as variant (Fig EV1B, see details in Materials and Methods).

Calculation of coefficient of variation

Coefficient of variation (CV) is a statistical measure that is used to calculate the dispersion of data. For every gene, CV was calculated by dividing standard deviation of expression across different samples (σ) (e.g., different tissues in case of tissue dataset) to the mean of expression across samples (μ). CV was empirically thresholded using the CV of known propionate shunt genes to keep the approach conservative.

$$\mathrm{CV} = \sigma/\mu \quad (3)$$

Categorizing genes based on expression variation in compendium

To be consistent with the approach used with the tissue dataset and to be conservative in our assessment, a CV threshold of 0.75 was selected and required that highly variant genes had a CV greater than or equal to this threshold in at least three datasets. Genes that showed CV < 0.3 in at least 95% of the datasets were labeled as invariant, and genes that fit into neither category were annotated as moderately variant (Fig EV3A). It was further assumed that genes that are not present in a dataset are lowly expressed, and were removed from the analysis.

Calculation of coexpression of gene pairs

The correlation in expression of metabolic gene pairs during development, across tissues, and across the compendium of gene expression studies was calculated based on Pearson correlation coefficient (PCC) using the Distancer function of Sleipnir library (Huttenhower *et al*, 2008). These correlations defined pairwise coexpression. Differences in the distribution of coexpression values between random, AND genes, OR genes, other paralogs, all pathway genes, and PW genes were evaluated using Mann–Whitney *U* test (Fay & Proschan, 2010).

Custom pathway enrichment analysis pipeline

Pathway-to-gene annotations from level 4 of WormPaths (Walker *et al*, 2021) were used as input gene sets. Each metabolic pathway (or category such as an enzyme complex; hereafter referred to as pathway for simplicity) consists of two or more annotated metabolic genes. The coexpression of genes in each metabolic pathway with all other genes in the metabolic network was extracted from the compendium. Subsequently, the mean of the correlations of pathway genes with all other metabolic genes (excluding the self-correlations) was calculated. The mean values were used to define a ranked list of metabolic genes for every metabolic pathway. GSEA was then performed on the preranked list of each pathway using the PreRank module (Subramanian *et al*, 2005). Enrichment score (ES) is the degree to which the genes in a gene set are overrepresented at the top or bottom of the entire ranked list of genes. Since the genes that are functionally related are mostly positively correlated, we only consider the genes at the top of the list, hence the ones positively contributing to ES. Leading edge subset enlists the gene hits before the peak while calculating ES, therefore consisting of genes that contribute the most to the enrichment score.

NES was derived for each pathway by normalizing the ES values to mean ES for all permutations of the gene sets. This accounts for differences in gene set size. FDR is the estimated probability that a gene set with a given NES represents a false-positive finding. The significance cutoff for the GSEA was set at an FDR value of ≤ or =0.05. If a pathway was found to be significantly self-enriched, it was categorized as coexpressed. We validated this result by running the custom pathway enrichment analysis pipeline on 1,000 randomized gene sets. For these randomizations, the structure of the data was maintained such that the correlation matrix, the number of genes in each pathway, and the number of times an individual gene was repeated across pathways all remained the same.

Semisupervised approach that combines Coflux and Coexpression

The first part of the approach involves using flux balance analysis (FBA) to simulate reaction rates (fluxes) in the metabolic network and then using a flux dependency metric, referred to as coflux, to measure pairwise associations of genes in the *C. elegans* metabolic network model (Yilmaz & Walhout, 2016; Yilmaz *et al*, 2020). We

© 2023 The Authors

examined all reaction pairs to see whether constraining the flux of one of the reactions to zero reduces the flux of the other (see below for the algorithm). The coflux value is zero for independent reactions and one for reactions that are fully coupled. Reactions that are connected by a junction to another reaction are usually partially dependent. After generating coflux values for each pair of reactions, we converted the reaction matrix to a pairwise gene coflux matrix using GPR associations. A high coflux value for a gene pair indicates that the genes encode enzymes acting in the same metabolic process. For the second part of the approach, a coexpression matrix was derived from the *C. elegans* gene expression compendium described above. All negative correlations were converted to zero to be consistent with the coflux matrix. The coflux and coexpression matrices were multiplied to obtain a product matrix. Since both coexpression and coflux values are between 0 and 1, a product takes a high value only if both coflux and coexpression values are high. Hierarchical clustering was then performed on the product matrix using dynamic cut tree algorithm using cutreehybrid package (Langfelder *et al*, 2008).

Coflux algorithm

The coflux value for each gene pair was calculated using FBA with iCEL1314 (Yilmaz *et al*, 2020). First, the standard bacterial diet was amended with a minimum set of nutrients (i.e., by allowing uptake through exchange reactions in the model as indicated in Dataset EV8) that warranted nonzero flux in all reactions of the model. Then, the following steps were taken to calculate coflux values:

- For every irreversible reaction i,
  - Calculate $v_{\max,i}$, the maximum flux that can be achieved with the intact network.
  - For every reaction j, calculate $v_{\max,ij}$, which is the maximum flux observed in reaction i when reaction j is constrained to a flux of zero. If i is included in a predefined set of 15 redundant reaction pairs (i.e., reactions with similar reactants and products except for differences such as the use of NADP instead of NAD as electron carrier, Dataset EV8), the flux of the corresponding reaction in the pair was also constrained to zero.
  - For every reaction j, calculate the coflux with i (c_{ij}) using equation (4).

$$c_{ij} = \frac{V_{\max,i} - V_{\max,ij}}{V_{\max,i}} \tag{4}$$

- For every reversible reaction i,
  - For every reaction j, repeat the above steps to calculate the coflux with i in forward direction ($c_{ij,\text{forward}}$).
  - Calculate $v_{\min,i}$, the minimum (i.e., the most negative, as negative flux indicates flux in reverse direction) flux that can be achieved with the intact network.
  - For every reaction j, calculate $v_{\min,ij}$, which is the minimum flux observed in reaction i when reaction j is constrained to a flux of zero. Once again, the reaction redundant with reaction i is also constrained to zero flux, if applicable.
  - For every reaction j, calculate the coflux with the reverse direction of i ($c_{ij,\text{reverse}}$) using equation (5).

$$c_{ij,\text{reverse}} = \frac{V_{\min,i} - V_{\min,ij}}{V_{\min,i}} \tag{5}$$

  - For every reaction j, calculate final coflux with i as the maximum of $c_{ij,\text{forward}}$ and $c_{ij,\text{reverse}}$ (equation 6).

$$c_{ij} = \max\left(c_{ij,\text{forward}}, abs\left(c_{ij,\text{reverse}}\right)\right) \tag{6}$$

- Since c_{ij} and c_{ji} are not necessarily equal, calculate final coflux value for every reaction pair using equation (7).

$$c_{ij,\text{final}} = \max\left(c_{ij}, c_{ji}\right) \tag{7}$$

- Convert the reaction coflux matrix to a gene coflux matrix based on gene–reaction associations. If a gene pair is associated through multiple reaction pairs (i.e., when at least one of the genes is associated with multiple reactions), take the maximum of coflux values between reactions to calculate gene coflux.

Hierarchical clustering

Hierarchical clustering was performed using average method of linkage on the dissimilarity matrix generated by 1 minus the product matrix value (coflux*coexpression). Dynamic cut tree algorithm from cutreehybrid package was used to cut the dendrogram generated by this clustering with stringent parameters deepSplit = 2 and minClusterSize = 3 and relatively relaxed parameters deepSplit = 3 and minClusterSize = 6 (Langfelder *et al*, 2008). The stringent setting was thresholded based on the occurrence of propionate shunt genes together in one cluster while keeping the size of the smallest cluster to be at least 3. The relaxed setting was chosen to capture larger clusters, such as the Met/SAM cycle genes in a single cluster.

Quantifying cluster quality through Silhouette score

Silhouette score determines the quality of clustering by measuring the cohesiveness of genes within the same cluster and separateness from the genes in the neighboring clusters (Rousseeuw, 1987). It was calculated using scikit-learn package (Pedregosa *et al* (2011). We first calculated silhouette score of each metabolic gene based on its placement in each cluster and then calculated mean silhouette score (MSS) for every cluster. Stringent clustering led to 197 clusters, ranging in size from three to 27 genes. We ranked these stringent clusters using MSS, where few of the top-ranked were inspected in more detail (Appendix Fig S3A). For relaxed clustering, we followed the same approach (Appendix Fig S3C).

Finding activation and repression conditions of metabolic clusters

To find activation and repression conditions of each cluster, we first calculated the mean coexpression of all gene pairs in that cluster in each dataset separately. To rank datasets that showed highest coexpression uniquely for each cluster, we normalized these mean coexpression values of all clusters using z-scoring across each dataset. We then identified the 30 best datasets that potentially represent activation/repression conditions of each cluster by the z-score values of mean coexpression. After this, we manually inspected each dataset in the order of decreasing mean coexpression, its

 © 2023 The Authors

associated published paper, and the heatmap to understand activation/repression conditions.

Gene-centric coexpression with metabolic (sub)-pathways by WormClust

We developed a custom computational pipeline that identifies coexpression of *C. elegans* genes with metabolic genes used in this study. The pathway gene sets were generated using WormPaths as a GMT (Gene Matrix Transposed) file, a tab-delimited file of gene sets (Walker *et al*, 2021). The ranked coexpression list of metabolic genes was extracted for each queried gene, from the global coexpression matrix generated using compendium of 177 datasets. The ranked list of each queried gene was used to run Gene Set Enrichment Analysis (GSEA) on the custom metabolic pathway gene sets.

Data availability

Gene clusters from semisupervised approach and pathway enrichment of all protein-coding genes outside the iCEL model, including but not limited to TFs, orphan metabolic genes, and transporters are available using WormClust on the WormFlux website (http://wormflux.umassmed.edu/WormClust/wormclust.php). Other data can be found in Datasets EV1–EV11. We also created a Github repository (https://github.com/WalhoutLab/WormClust) for this project, which includes scripts that generated results presented here.

Expanded View for this article is available online.

Acknowledgements

We thank members of the Walhout laboratory and Caryn Navarro, for discussion and critical reading of the manuscript. This work was supported by National Institute of Health (NIH) grant R35GM122502 to AJMW and R01HG005084 to CLM.

Author contributions

Albertha JM Walhout: Conceptualization; supervision; funding acquisition; writing – original draft; project administration; writing – review and editing. **Shivani Nanda:** Conceptualization; software; formal analysis; investigation; methodology; writing – original draft; writing – review and editing. **Marc-Antoine Jacques:** Conceptualization; investigation; methodology; writing – review and editing. **Wen Wang:** Methodology. **Chad L Myers:** Supervision; methodology; writing – review and editing. **L Safak Yilmaz:** Conceptualization; formal analysis; supervision; investigation; methodology; writing – review and editing.

Disclosure and competing interests statement

The authors declare that they have no conflict of interest. AJMW is an editorial advisory board member. This has no bearing on the editorial consideration of this article for publication.

References

Arda HE, Taubert S, Conine C, Tsuda B, Van Gilst MR, Sequerra R, Doucette-Stam L, Yamamoto KR, Walhout AJM (2010) Functional modularity of nuclear hormone receptors in a *C. elegans* gene regulatory network. *Mol Syst Biol* 6: 367

Bhattacharya S, Horowitz BB, Zhang J, Li X, Zhang H, Giese GE, Holdorf AD, Walhout AJM (2022) A metabolic regulatory network for the *Caenorhabditis elegans* intestine. *iScience* 25: 104688

Blangy D, Buc H, Monod J (1968) Kinetics of the allosteric interactions of phosphofructokinase from *Escherichia coli*. *J Mol Biol* 31: 13–35

Blumenthal T, Evans D, Link CD, Guffanti A, Lawson D, Thierry-Mieg J, Thierry-Mieg D, Chiu WL, Duke K, Kiraly M *et al* (2002) A global analysis of *Caenorhabditis elegans* operons. *Nature* 417: 851–854

Brosnan ME, Brosnan JT (2020) Histidine metabolism and function. *J Nutr* 150: 2570S–2575S

Brown MS, Goldstein JL (1997) The SREBP pathway: regulation of cholesterol metabolism by proteolysis of a membrane-bound transcription factor. *Cell* 89: 331–340

Bulcha JT, Giese GE, Ali MZ, Lee Y-U, Walker M, Holdorf AD, Yilmaz LS, Brewster R, Walhout AJM (2019) A persistence detector for metabolic network rewiring in an animal. *Cell Rep* 26: 460–468

Cao J, Packer JS, Ramani V, Cusanovich DA, Huynh C, Daza R, Qiu X, Lee C, Furlan SN, Steemers FJ *et al* (2017) Comprehensive single-cell transcriptional profiling of a multicellular organism. *Science* 357: 661–667

Caputto R, Leloir LR, Trucco RE, Cardini CE, Paladini AC (1949) The enzymatic transformation of galactose into glucose derivatives. *J Biol Chem* 179: 497–498

Daschner K, Couee I, Binder S (2001) The mitochondrial isovaleryl-coenzyme a dehydrogenase of arabidopsis oxidizes intermediates of leucine and valine catabolism. *Plant Physiol* 126: 601–612

DeBerardinis RJ, Thompson CB (2012) Cellular metabolism and disease: what do metabolic outliers teach us? *Cell* 148: 1132–1144

DeBose-Boyd RA, Ye J (2018) SREBPs in lipid metabolism, insulin signaling, and beyond. *Trends Biochem Sci* 43: 358–368

Ducker GS, Rabinowitz JD (2017) One-carbon metabolism in health and disease. *Cell Metab* 25: 27–42

Eisen MB, Spellman PT, Brown PO, Botstein D (1998) Cluster analysis and display of genome-wide expression patterns. *Proc Natl Acad Sci USA* 95: 14863–14868

Falk MJ, Zhang Z, Rosenjack JR, Nissim I, Daikhin E, Nissim I, Sedensky MM, Yudkoff M, Morgan PG (2008) Metabolic pathway profiling of mitochondrial respiratory chain mutants in *C. elegans*. *Mol Genet Metab* 93: 388–397

Fay MP, Proschan MA (2010) Wilcoxon-Mann-Whitney or t-test? On assumptions for hypothesis tests and multiple interpretations of decision rules. *Stat Surv* 4: 1–39

Giese GE, Walker MD, Ponomarova O, Zhang H, Li X, Minevich G, Walhout AJ (2020) *Caenorhabditis elegans* methionine/S-adenosylmethionine cycle activity is sensed and adjusted by a nuclear hormone receptor. *Elife* 9: e60259

Gilbert W, Muller-Hill B (1966) Isolation of the lac repressor. *Proc Natl Acad Sci USA* 56: 1891–1898

Hahn-Windgassen A, Van Gilst MR (2009) The *Caenorhabditis elegans* HNF4alpha homolog, NHR-31, mediates excretory tube growth and function through coordinate regulation of the vacuolar ATPase. *PLoS Genet* 5: e1000553

Harris CR, Millman KJ, van der Walt SJ, Gommers R, Virtanen P, Cournapeau D, Wieser E, Taylor J, Berg S, Smith NJ *et al* (2020a) Array programming with NumPy. *Nature* 585: 357–362

Harris TW, Arnaboldi V, Cain S, Chan J, Chen WJ, Cho J, Davis P, Gao S, Grove CA, Kishore R *et al* (2020b) WormBase: a modern model organism information resource. *Nucleic Acids Res* 48: D762–D767

© 2023 The Authors

Hopper JE, Broach JR, Rowe LB (1978) Regulation of the galactose pathway in *Saccharomyces cerevisiae*: induction of uridyl transferase mRNA and dependency on GAL4 gene function. *Proc Natl Acad Sci USA* 75: 2878 – 2882

Hughes TR, Marton MJ, Jones AR, Roberts CJ, Stoughton R, Armour CD, Bennett HA, Coffey E, Dai H, He YD *et al* (2000) Functional discovery via a compendium of expression profiles. *Cell* 102: 109 – 126

Huttenhower C, Schroeder M, Chikina MD, Troyanskaya OG (2008) The Sleipnir library for computational functional genomics. *Bioinformatics* 24: 1559 – 1561

Ihmels J, Levy R, Barkai N (2004) Principles of transcriptional control in the metabolic network of *Saccharomyces cerevisiae*. *Nat Biotechnol* 22: 86 – 92

Jacob F, Monod J (1961) Genetic regulatory mechanisms in the synthesis of proteins. *J Mol Biol* 3: 318 – 356

Jansen R, Greenbaum D, Gerstein M (2002) Relating whole-genome expression data with protein-protein interactions. *Genome Res* 12: 37 – 46

Jia F, Cui M, Than MT, Han M (2016) Developmental defects of *Caenorhabditis elegans* lacking branched-chain alpha-Ketoacid dehydrogenase are mainly caused by monomethyl branched-chain fatty acid deficiency. *J Biol Chem* 291: 2967 – 2973

Kharchenko P, Church GM, Vitkup D (2005) Expression dynamics of a cellular metabolic network. *Mol Syst Biol* 1: 2005.0016

Kim SK, Lund J, Kiraly M, Duke K, Jiang M, Stuart JM, Eizinger A, Wylie BN, Davidson GS (2001) A gene expression map for *Caenorhabditis elegans*. *Science* 293: 2087 – 2092

Kim HU, Kim TY, Lee SY (2008) Metabolic flux analysis and metabolic engineering of microorganisms. *Mol Biosyst* 4: 113 – 120

Kim D, Grun D, van Oudenaarden A (2013) Dampening of expression oscillations by synchronous regulation of a microRNA and its target. *Nat Genet* 45: 1337 – 1344

Kim B, Suo B, Emmons SW (2016) Gene function prediction based on developmental transcriptomes of the two sexes in *C. elegans*. *Cell Rep* 17: 917 – 928

Lai CH, Chou CY, Ch'ang LY, Liu CS, Lin W (2000) Identification of novel human genes evolutionarily conserved in *Caenorhabditis elegans* by comparative proteomics. *Genome Res* 10: 703 – 713

Langfelder P, Zhang B, Horvath S (2008) Defining clusters from a hierarchical cluster tree: the dynamic tree cut package for R. *Bioinformatics* 24: 719 – 720

Ledezma-Tejeida D, Ishida C, Collado-Vides J (2017) Genome-wide mapping of transcriptional regulation and metabolism describes information-processing units in *Escherichia coli*. *Front Microbiol* 8: 1466

Liu W, Li L, He Y, Cai S, Zhao W, Zheng H, Zhong Y, Wang S, Zou Y, Xu Z *et al* (2018) Functional annotation of *Caenorhabditis elegans* genes by analysis of gene Co-expression networks. *Biomolecules* 8: 70

MacNeil LT, Walhout AJM (2011) Gene regulatory networks and the role of robustness and stochasticity in the control of gene expression. *Genome Res* 21: 645 – 657

MacNeil LT, Watson E, Arda HE, Zhu LJ, Walhout AJM (2013) Diet-induced developmental acceleration independent of TOR and insulin in *C. elegans*. *Cell* 153: 240 – 252

MacNeil LT, Pons C, Arda HE, Giese GE, Myers CL, Walhout AJM (2015) Transcription factor activity mapping of a tissue-specific gene regulatory network. *Cell Syst* 1: 152 – 162

Mori A, Holdorf AD, Walhout AJM (2017) Many transcription factors contribute to *C. elegans* growth and fat storage. *Genes Cells* 22: 770 – 784

Nhan JD, Turner CD, Anderson SM, Yen CA, Dalton HM, Cheesman HK, Ruter DL, Uma Naresh N, Haynes CM, Soukas AA *et al* (2019) Redirection of SKN-1 abates the negative metabolic outcomes of a perceived pathogen infection. *Proc Natl Acad Sci USA* 116: 22322 – 22330

Pedregosa F, Varoquaux G, Gramfort A, Michel V, Thirion B, Grisel O, Blondel M, Prettenhofer P, Weiss R, Dubourg V *et al* (2011) Scikit-learn: machine learning in Python. *JMLR* 12: 2825 – 2830

Ponomarova O, Zhang H, Li X, Nanda S, Leland TB, Fox BW, Giese GE, Schroeder FC, Yilmaz LS, Walhout AJM (2022) D-2-Hydroxyglutarate dehydrogenase connects the propionate shunt to ketone body metabolism in *Caenorhabditis elegans*. *bioRxiv* https://doi.org/10.1101/2022.05.16.492161 [PREPRINT]

Reece-Hoyes JS, Pons C, Diallo A, Mori A, Shrestha S, Kadreppa S, Nelson J, DiPrima S, Dricot A, Lajoie BR *et al* (2013) Extensive rewiring and complex evolutionary dynamics in a *C. elegans* multiparameter transcription factor network. *Mol Cell* 51: 116 – 127

Reinke V, Smith HE, Nance J, Wang J, Van Doren C, Begley R, Jones SJM, Davis EB, Scherer S, Ward S *et al* (2000) A global profile of germline gene expression in *C. elegans*. *Mol Cell* 6: 605 – 616

Rousseeuw PJ (1987) Silhouettes: a graphical aid to interpretation and validation of cluster analysis. *J Comput Appl Math* 20: 53 – 65

Schirmer T, Evans PR (1990) Structural basis of the allosteric behaviour of phosphofructokinase. *Nature* 343: 140 – 145

Segal E, Shapira M, Regev A, Pe'er D, Botstein D, Koller D, Friedman N (2003) Module networks: identifying regulatory modules and their condition-specific regulators from gene expression data. *Nat Genet* 34: 166 – 176

Segal E, Friedman N, Koller D, Regev A (2004) A module map showing conditional activity of expression modules in cancer. *Nat Genet* 36: 1090 – 1098

Seshasayee AS, Fraser GM, Babu MM, Luscombe NM (2009) Principles of transcriptional regulation and evolution of the metabolic system in *E. coli*. *Genome Res* 19: 79 – 91

Sharma MD, Garber AJ, Farmer JA (2008) Role of insulin signaling in maintaining energy homeostasis. *Endocr Pract* 14: 373 – 380

Shaye DD, Greenwald I (2011) OrthoList: a compendium of *C. elegans* genes with human orthologs. *PLoS ONE* 6: e20085

Spencer WC, Zeller G, Watson JD, Henz SR, Watkins KL, McWhirter RD, Petersen S, Sreedharan VT, Widmer C, Jo J *et al* (2011) A spatial and temporal map of *C. elegans* gene expression. *Genome Res* 21: 325 – 341

Stuart JM, Segal E, Koller D, Kim SK (2003) A gene-coexpression network for global discovery of conserved genetic modules. *Science* 302: 249 – 255

Subramanian A, Tamayo P, Mootha VK, Mukherjee S, Ebert BL, Gillette MA, Paulovich A, Pomeroy SL, Golub TR, Lander ES *et al* (2005) Gene set enrichment analysis: a knowledge-based approach for interpreting genome-wide expression profiles. *Proc Natl Acad Sci USA* 102: 15545 – 15550

Sulston JE, Horvitz HR (1977) Post-embryonic cell lineages of the nematode, *Caenorhabditis elegans*. *Dev Biol* 56: 110 – 156

Tang M, Li B, Zhou X, Bolt T, Li JJ, Cruz N, Gaudinier A, Ngo R, Clark-Wiest C, Kliebenstein DJ *et al* (2021) A genome-scale TF-DNA interaction network of transcriptional regulation of Arabidopsis primary and specialized metabolism. *Mol Syst Biol* 17: e10625

Thiele I, Palsson BO (2010) A protocol for generating a high-quality genome-scale metabolic reconstruction. *Nat Protoc* 5: 93 – 121

Van Gilst MR, Hajivassiliou H, Jolly A, Yamamoto KR (2005) Nuclear hormone receptor NHR-49 controls fat consumption and fatty acid composition in *C. elegans*. *PLoS Biol* 3: e53

van Waveren C, Moraes CT (2008) Transcriptional co-expression and co-regulation of genes coding for components of the oxidative phosphorylation system. *BMC Genomics* 9: 18

Virtanen P, Gommers R, Oliphant TE, Haberland M, Reddy T, Cournapeau D, Burovski E, Peterson P, Weckesser W, Bright J *et al* (2020) SciPy 1.0:

 © 2023 The Authors

fundamental algorithms for scientific computing in python. *Nat Methods* 17: 261–272

Walker AK, Jacobs RL, Watts JL, Rottiers V, Jiang K, Finnegan DM, Shioda T, Hansen M, Yang F, Niebergall LJ *et al* (2011) A conserved SREBP-1/ phosphatidylcholine feedback circuit regulates lipogenesis in metazoans. *Cell* 147: 840–852

Walker MD, Giese GE, Holdorf AD, Bhattacharya S, Diot C, Garcia-Gonzalez AP, Horowitz BB, Lee YU, Leland T, Li X *et al* (2021) WormPaths: *Caenorhabditis elegans* metabolic pathway annotation and visualization. *Genetics* 219: iyab089

Wallace M, Green CR, Roberts LS, Lee YM, McCarville JL, Sanchez-Gurmaches J, Meurs N, Gengatharan JM, Hover JD, Phillips SA *et al* (2018) Enzyme promiscuity drives branched-chain fatty acid synthesis in adipose tissues. *Nat Chem Biol* 14: 1021–1031

Watson E, MacNeil LT, Arda HE, Zhu LJ, Walhout AJM (2013) Integration of metabolic and gene regulatory networks modulates the *C. elegans* dietary response. *Cell* 153: 253–266

Watson E, MacNeil LT, Ritter AD, Yilmaz LS, Rosebrock AP, Caudy AA, Walhout AJM (2014) Interspecies systems biology uncovers metabolites affecting *C. elegans* gene expression and life history traits. *Cell* 156: 759–770

Watson E, Olin-Sandoval V, Hoy MJ, Li C-H, Louisse T, Yao V, Mori A, Holdorf AD, Troyanskaya OG, Ralser M *et al* (2016) Metabolic network rewiring of propionate flux compensates vitamin B12 deficiency in *C. elegans*. *Elife* 5: e17670

Yanai I, Hunter CP (2009) Comparison of diverse developmental transcriptomes reveals that coexpression of gene neighbors is not evolutionarily conserved. *Genome Res* 19: 2214–2220

Yilmaz LS, Walhout AJ (2016) A *Caenorhabditis elegans* genome-scale metabolic network model. *Cell Syst* 2: 297–311

Yilmaz LS, Walhout AJ (2017) Metabolic network modeling with model organisms. *Curr Opin Chem Biol* 36: 32–39

Yilmaz LS, Li X, Nanda S, Fox B, Schroeder F, Walhout AJ (2020) Modeling tissue-relevant *Caenorhabditis elegans* metabolism at network, pathway, reaction, and metabolite levels. *Mol Syst Biol* 16: e9649

Zeng L, Li X, Preusch CB, He GJ, Xu N, Cheung TH, Qu J, Mak HY (2021) Nuclear receptors NHR-49 and NHR-79 promote peroxisome proliferation to compensate for aldehyde dehydrogenase deficiency in *C. elegans*. *PLoS Genet* 17: e1009635

License: This is an open access article under the terms of the Creative Commons Attribution License, which permits use, distribution and reproduction in any medium, provided the original work is properly cited.

Expanded View Figures

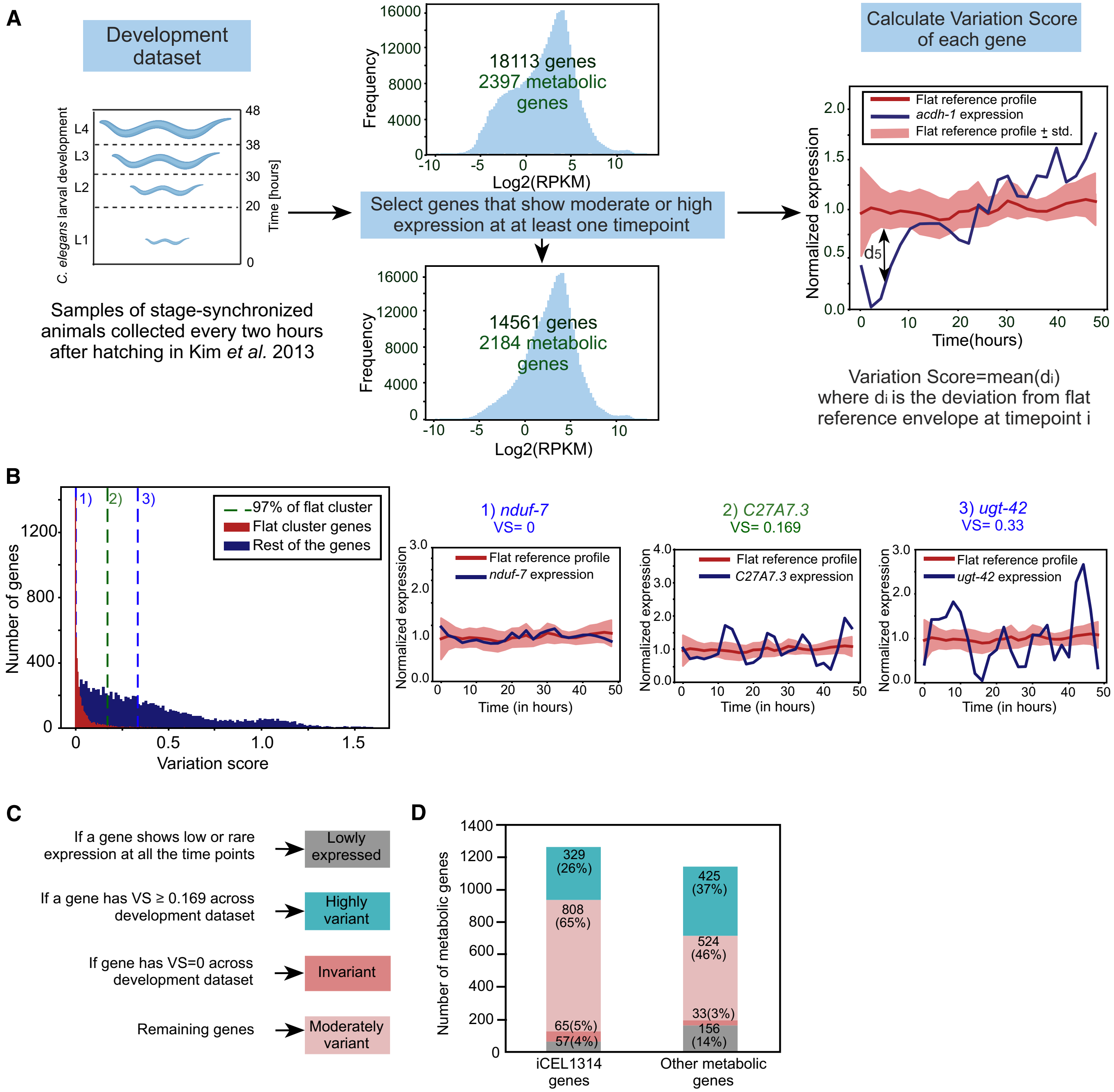

Figure EV1.

22 of 21 *Molecular Systems Biology* **19**: e11443 | 2023 © 2023 The Authors

Figure EV1. Transcriptional regulation of metabolism during development.

A Computational pipeline to identify highly variant metabolic genes during development. Genes that showed either moderate or high expression in at least one time point were selected, reducing the number of genes from 18,113 (2,397 metabolic) to 14,561 (2,184 metabolic). For each gene, VS was calculated using the deviation from a flat reference profile at each time point (see Materials and Methods). The red line indicates the mean value and light red shaded area the standard deviation of the flat reference profile. The profile of *acdh-1* is plotted in blue as an example of a developmentally regulated gene. d_5 denotes the deviation of *acdh-1* expression from the flat reference profile at the 5th data point.

B Distribution of VS of genes belonging to the flat cluster versus those belonging to other clusters. The vertical lines at 1 and 3 represent the iCEL1314 genes *nduf-7* and *ugt-42*, which have the lowest and highest VS of the flat set, respectively. The vertical line at 2 indicates the gene *C27A7.3* with selected threshold of VS at the 97% quantile of the flat cluster (VS = 0.169).

C Diagram showing criterion of categorizing metabolic and nonmetabolic genes into four categories across development: lowly expressed, invariant, moderately variant and highly variant.

D Bar graph shows the distinction of low expressed, invariant, moderately variant and highly variant genes during development in iCEL1314 and other metabolic genes. Color legend as indicated in (C).

Figure EV2. Transcriptional regulation of metabolic genes across different tissues.

A Computational pipeline to determine highly variant metabolic genes across tissues. Genes that show either moderate or high expression in at least one tissue are selected for analysis reducing the number of *C. elegans* genes from 19,675 (2,491 metabolic) to 13,305 (2,143 metabolic). The coefficient of variation (CV) of each gene was calculated by dividing the standard deviation of expression across tissues by the mean expression. Different thresholds of CV were titrated to select a stringent CV to categorize genes as variant and therefore potentially transcriptionally regulated. Examples for each threshold are provided in (B).

B Tissue expression and CV values of propionate shunt genes.

C Bar graphs showing expression profiles of example genes across tissues with CV 0.15, 0.3, 0.45, 0.6, 0.75, 0.9, 1.05 and 1.2. Numbering of examples is according to the corresponding threshold lines in (A).

D Diagram showing criterion of categorizing metabolic and nonmetabolic genes into four categories across tissues: lowly expressed, invariant, moderately variant and highly variant.

E Bar graph shows the distinction of low expressed, invariant, moderately variant and highly variant genes across tissues in iCEL1314 and other metabolic genes. Color legend as indicated in (D).

© 2023 The Authors

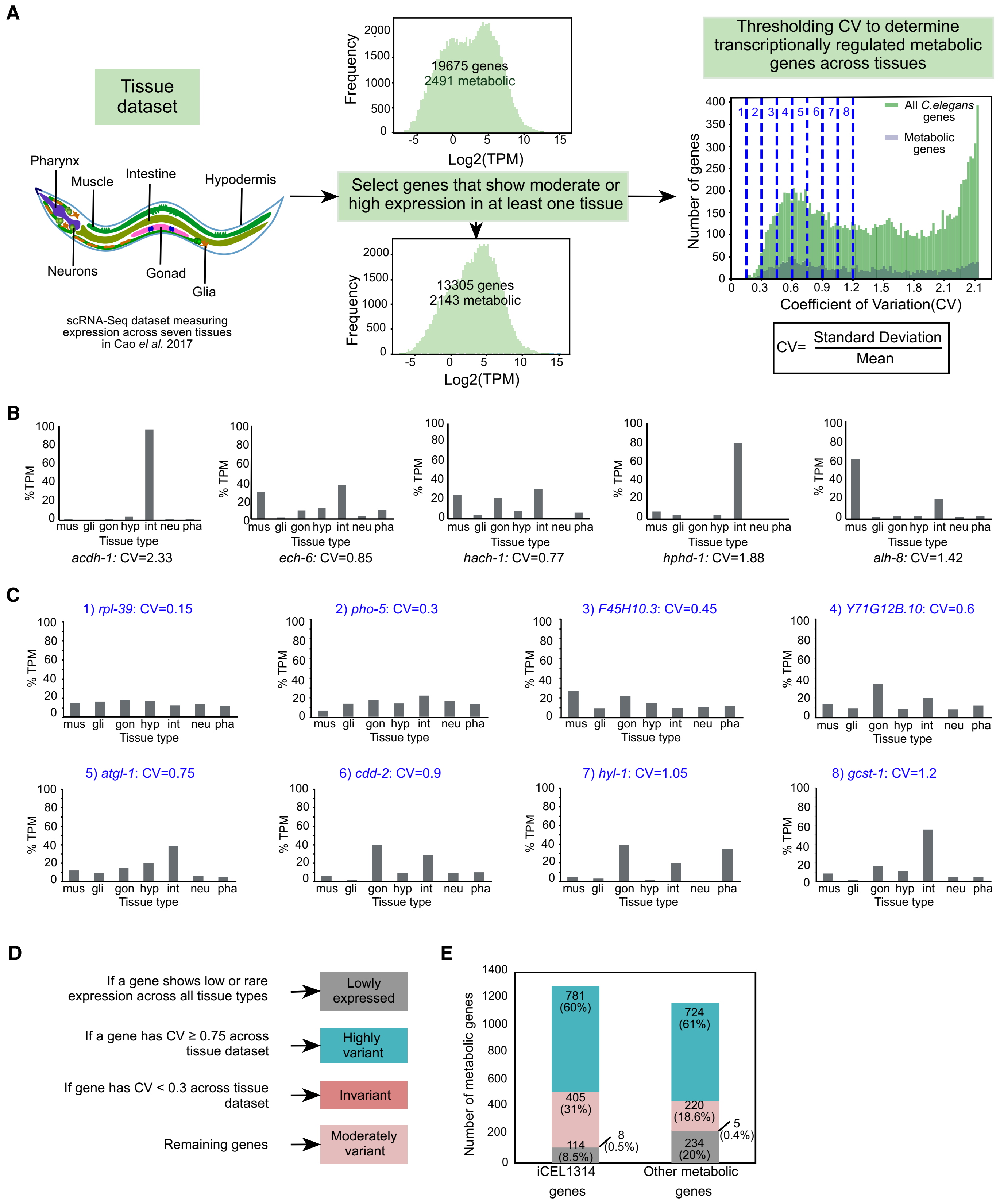

Figure EV2.

 © 2023 The Authors

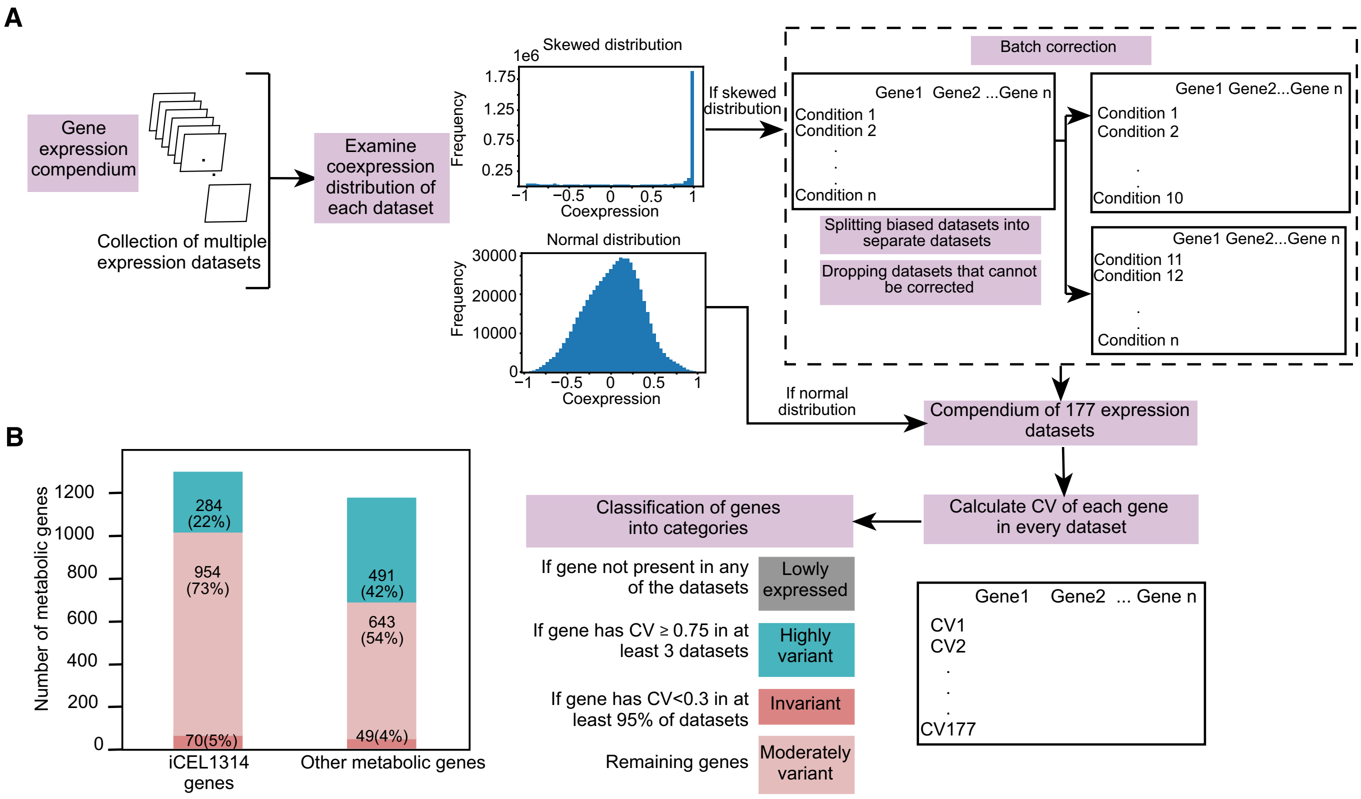

Figure EV3. Transcriptional regulation of metabolic genes across a compendium.

A Computational pipeline to determine transcriptionally regulated metabolic genes across the gene expression compendium. Datasets were evaluated for batch effects using correlation distribution. Some skewed datasets were corrected for batch effect by splitting into separate datasets, while some were removed if the source of skewness was not clear. This resulted in 177 datasets for analyses. CV of each gene was calculated. The criterion of classifying genes is based on the number of datasets with high CV and fraction of datasets with low CV.

B Bar graph showing low expressed, invariant, moderately variant and highly variant genes in the compendium in iCEL1314 and other metabolic genes. Color legend as indicated in (A).

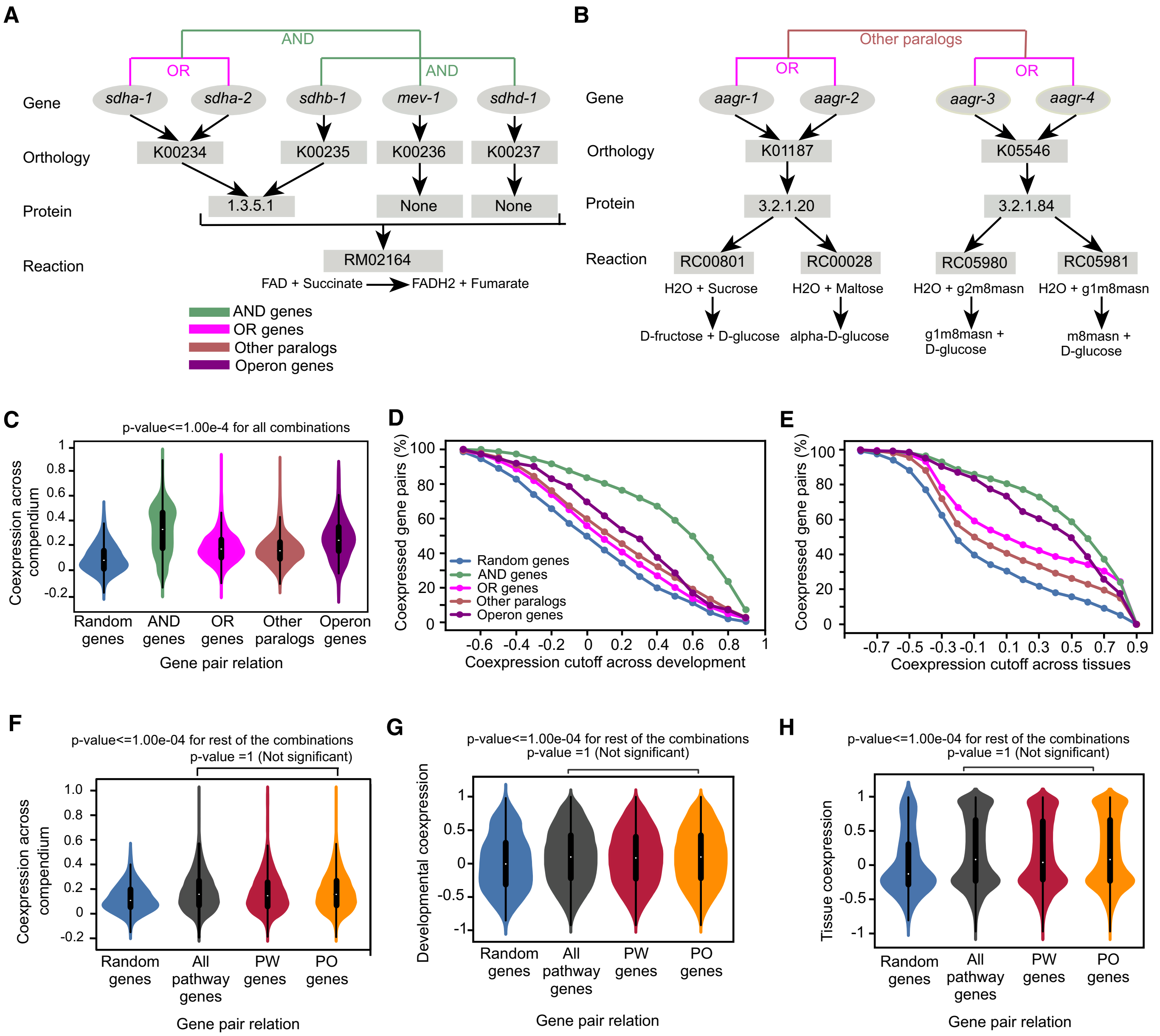

Figure EV4. Reaction-level analysis of metabolic pathways.

A The conversion of succinate to fumarate, which is part of complex II of the ETC and of the TCA cycle, is carried out by succinate dehydrogenase. Diagram showing that succinate dehydrogenase is composed of the OR genes *sdha-1* and *sdha-2* that each function together with the rest of the genes as AND genes. The GPR of this reaction is noted as [(*sdha-1* I *sdha-2*) & *sdhb-1*] & *mev-1* & *sdhd-1*]. Color legend is indicated.

B Example of a gene family (*aagr*) where members occur as paralogous OR gene pairs in separate reactions. Pairs of paralogs associated with different reactions are called other paralogs.

C Violin plot comparing coexpression for different populations of gene pairs including random, AND, OR, operon and other paralogs gene pairs in compendium of expression datasets.

D, E Percentages of AND, OR, other paralogs, operon genes and random metabolic gene pairs categorized as coexpressed using different coexpression values as cutoffs across developmental time (D) and tissues (E). Color legend as indicated in (D).

F–H Violin plots showing coexpression of random gene pairs, all pathway genes, PW genes and PO genes across compendium (F), different developmental stages (G) and tissues (H).

 © 2023 The Authors

Figure EV5. Semisupervised clustering of product matrix using relaxed parameters.

A Distinct cluster of known coregulated metabolic pathway propionate shunt genes together with isoleucine and valine degradation genes obtained using relaxed clustering parameters (deepSplit = 3, minClusterSize = 6) with the dynamic cut tree algorithm.

B Clusters of Met/SAM cycle genes and genes of related pathways obtained using relaxed (deepSplit = 3, minClusterSize = 6) and stringent (deepSplit = 2, minClusterSize = 3) parameters. Stringent clusters are shown near the drawn pathways of clustered genes.

C Pathway boundary (green shade) within selenocompound metabolism defined by genes in a high-quality cluster obtained by relaxed parameters and shown by the clustered heatmap (left). Mountain plots showing comparison of self-enrichment analysis statistics (NES, ES and FDR) of selenocompound metabolism from WormPaths with that of selenocompound cluster obtained by the semisupervised approach (right).

© 2023 The Authors

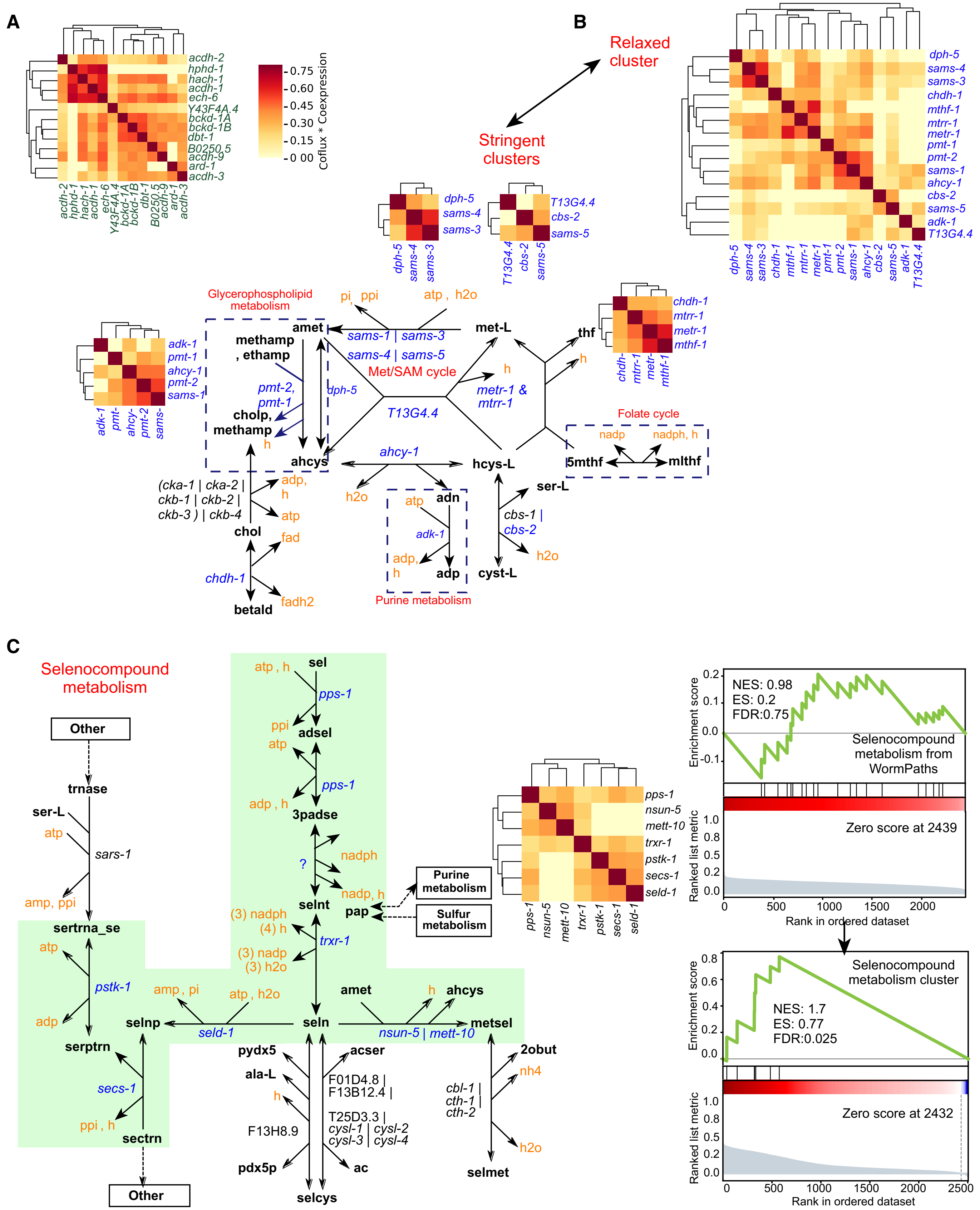

Figure EV5.

 © 2023 The Authors

APPENDIX

Systems-level transcriptional regulation of *Caenorhabditis elegans* metabolism

Shivani Nanda[1], Marc-Antoine Jacques[1,3], Wen Wang[2], Chad L Myers[2], L. Safak Yilmaz[1], Albertha JM Walhout[1*]

1. Department of Systems Biology, University of Massachusetts Chan Medical School, Worcester, MA 01655, United States
2. Department of Computer Science and Engineering, University of Minnesota, Minneapolis, MN 55455, United States
3. Current address: Cancer Research UK Cambridge Institute, University of Cambridge, Cambridge CB2 0RE, United Kingdom; and EMBL's European Bioinformatics Institute (EBI), Cambridgeshire CB10 1SD United Kingdom

CONTENTS

Supplementary Figures

Appendix Figure S1………………………………………..……………………..……..…3
Appendix Figure S2…………………………..…...…………..…………………………………4
Appendix Figure S3………………………..……………………………..………………………6
Appendix Figure S4………………………………..………………....…………………………7
Appendix Figure S5……………………...……………………...……...…………………………9

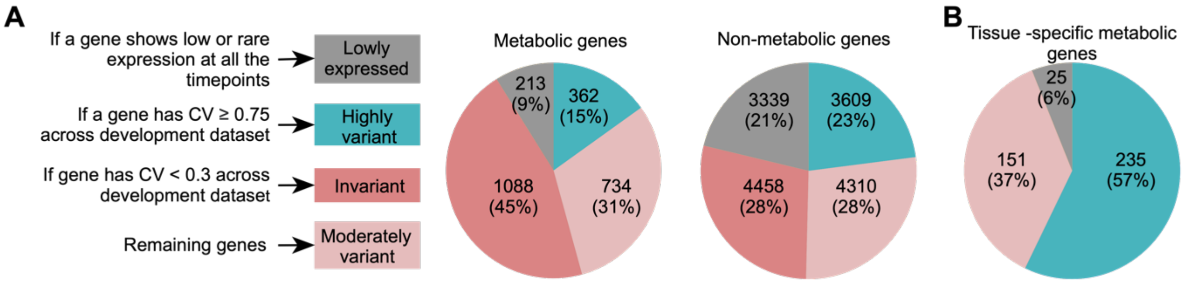

Appendix Figure S1. Transcriptional Regulation of Metabolism Across Development

(A) Diagram showing alternative criterion using CV for categorizing metabolic and non-metabolic genes into four categories across development: lowly expressed, invariant, moderately variant and highly variant (left). This analysis was done to make sure the result that metabolic genes are more highly variant across tissues than development is not driven by the use of different statistical approaches (*i.e.*, CV in tissue data set and VS in development). To eliminate this effect, we reevaluated the development dataset with the CV approach using the same threshold as with tissues, and found that, the percentage of highly variant genes during development was lowered from 31% to 15% with this method. Thus, using the same metric resulted in an even greater difference between metabolic genes exhibiting variation across tissues versus those exhibiting variation during development. Pie charts showing metabolic (center) and non-metabolic (right) gene expression variation in the development dataset using CV as criterion.

(B) Pie chart showing percentage of tissue-specific genes showing high variation in expression across development dataset.

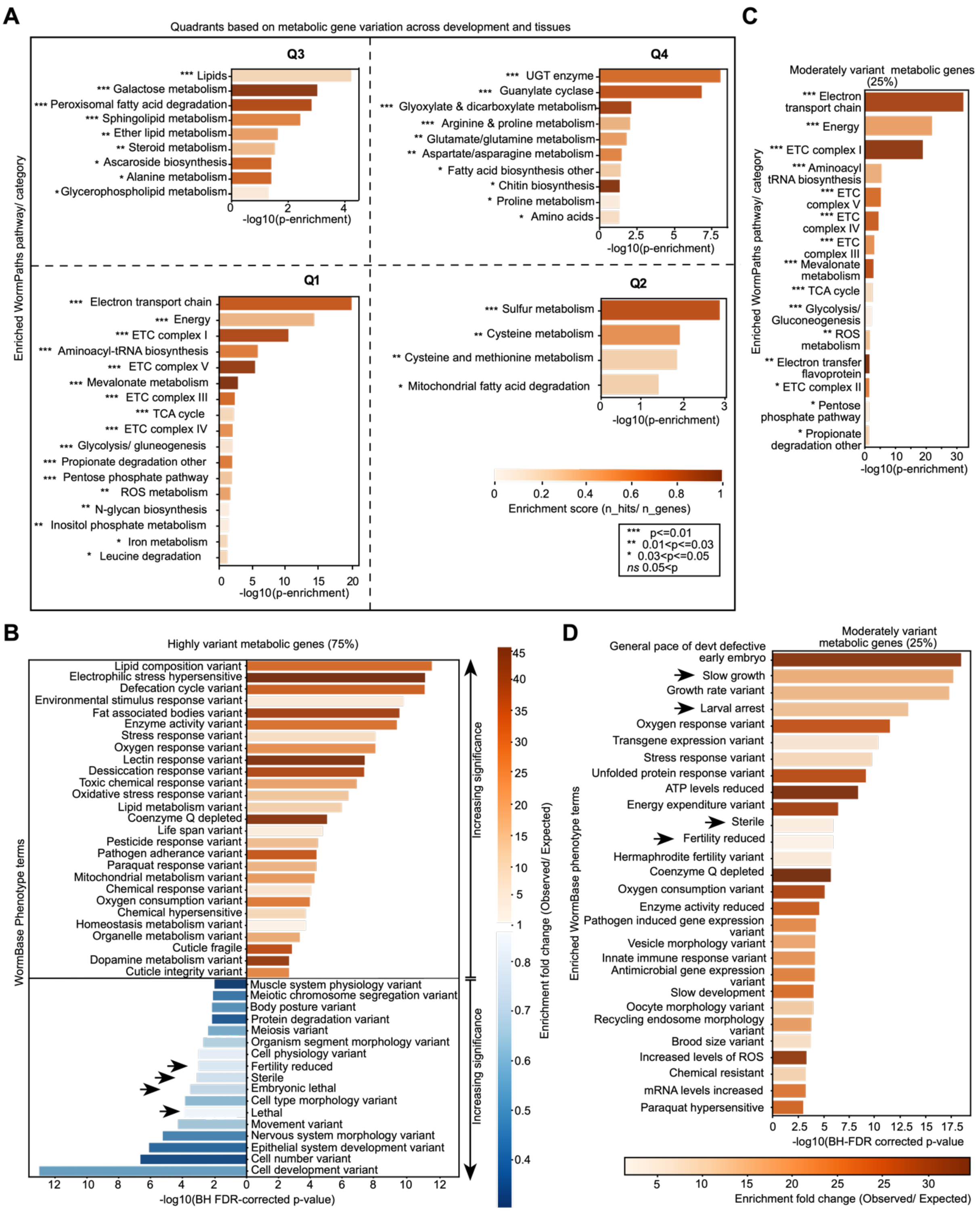

Appendix Figure S2. Pathway and Phenotypic Enrichment Analysis of Metabolic Genes Based on Expression Variation

(A) Bar graph showing enriched WormPaths pathways/categories for iCEL1314 genes in the four quadrants Q1, Q2, Q3 and Q4 in Figure 1H. The significance levels are indicated by asterisks or '*ns*'(not significant). Not significant (*ns)* pathways are not shown.

(B) Bar graph showing phenotypes enriched for highly variant metabolic genes (FDR-corrected p-value≤0.001).

(C) Bar graph showing WormPaths pathways/ categories enriched for moderately variant metabolic. The significance levels legend as indicated in (A).

(D) Bar graph showing phenotypes enriched for moderately variant metabolic genes (FDR-corrected p-value≤0.001)

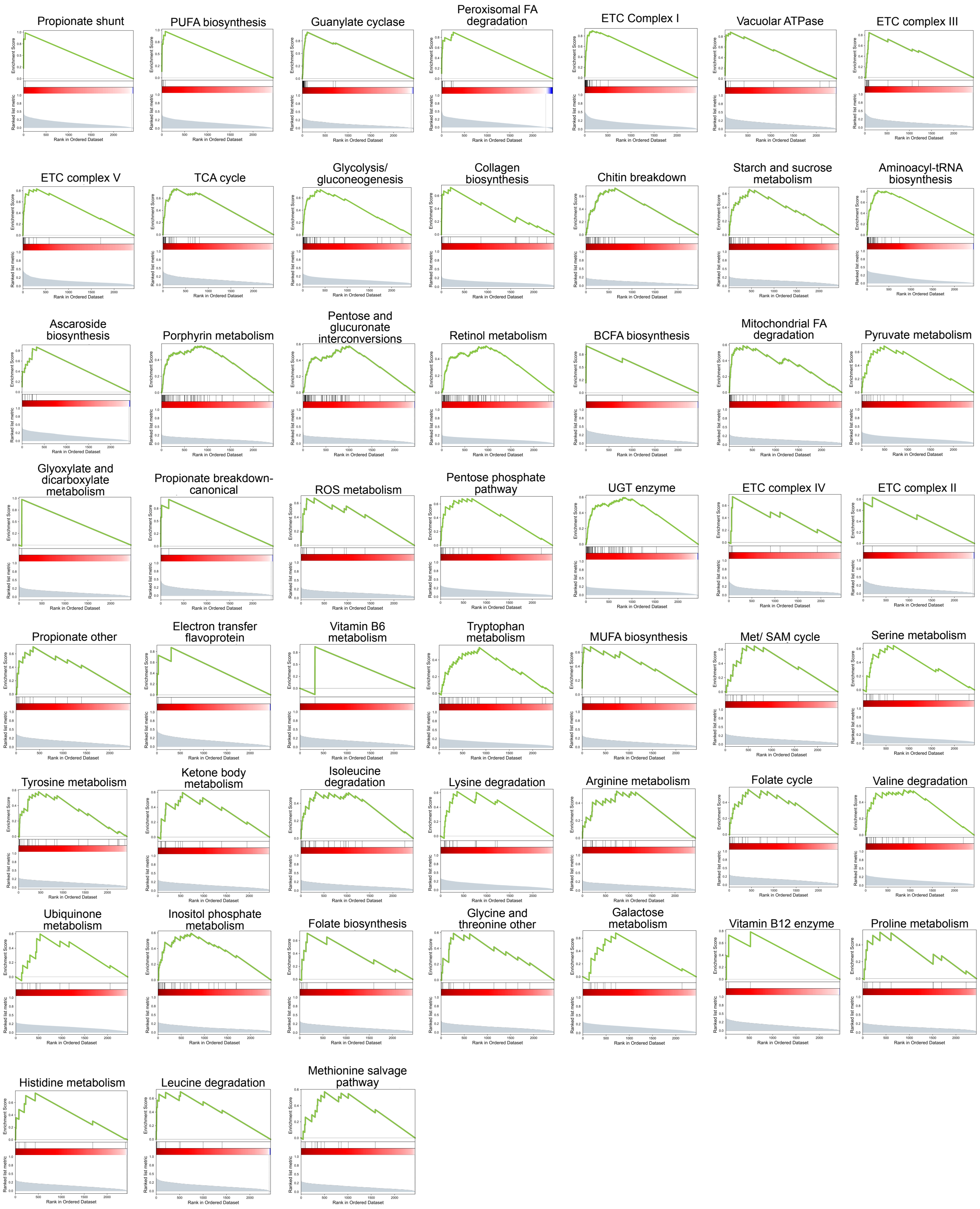

Appendix Figure S3. Mountain Plots Showing WormPaths Pathways Significantly Enriched For Coexpression in Order of Decreasing Significance

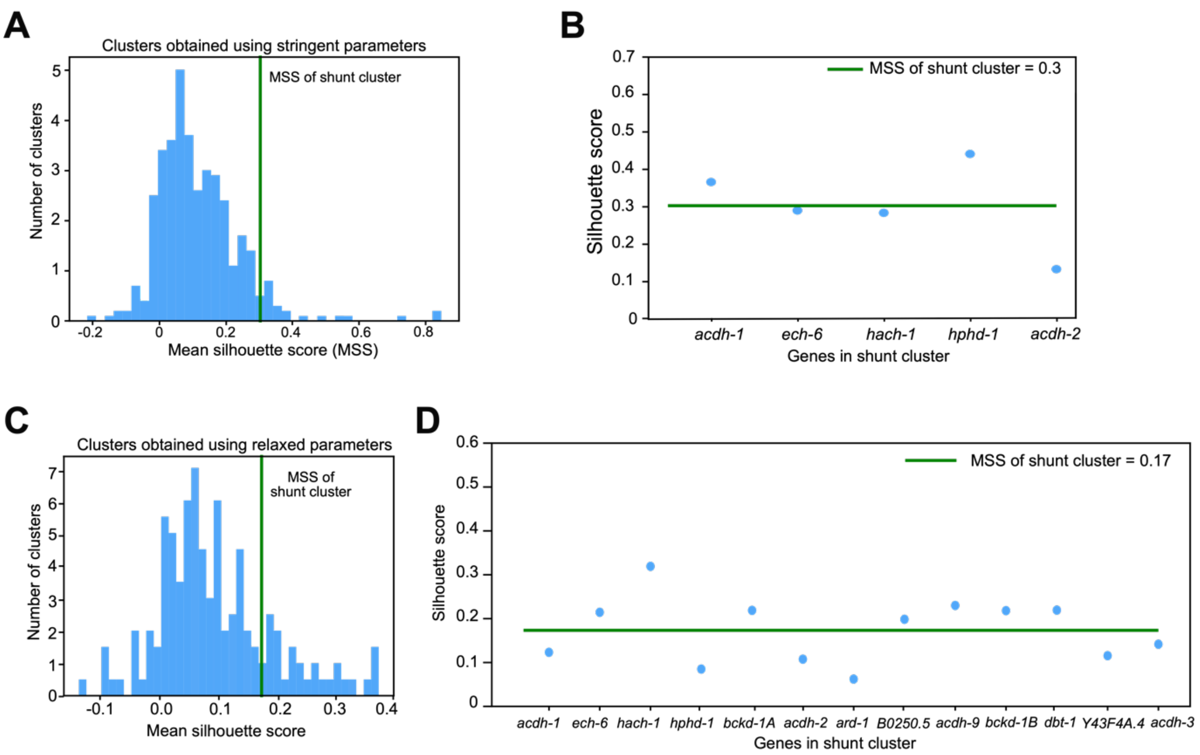

Appendix Figure S4. Mean Silhouette Score (MSS) to Evaluate Cluster Quality of Stringent and Relaxed Clusters

(A) Plot showing the distribution of the MSS of all clusters obtained using dynamic cut tree algorithm with stringent parameters (deepSplit=2, minClusterSize=3). The green vertical line shows the MSS of shunt cluster.

(B) Scatter plot with individual silhouette scores of genes in the propionate shunt cluster, with stringent parameters. MSS of this pathway is shown.

(C) Plot showing the distribution of the MSS of all clusters obtained using dynamic cut tree algorithm with relaxed parameters (deepSplit=3, minClusterSize=6). The green vertical line shows the MSS of shunt cluster.

(D) Scatter plot with individual silhouette scores of the cluster containing propionate shunt genes, with relaxed parameters. MSS of this pathway is shown along with the overall MSS threshold.

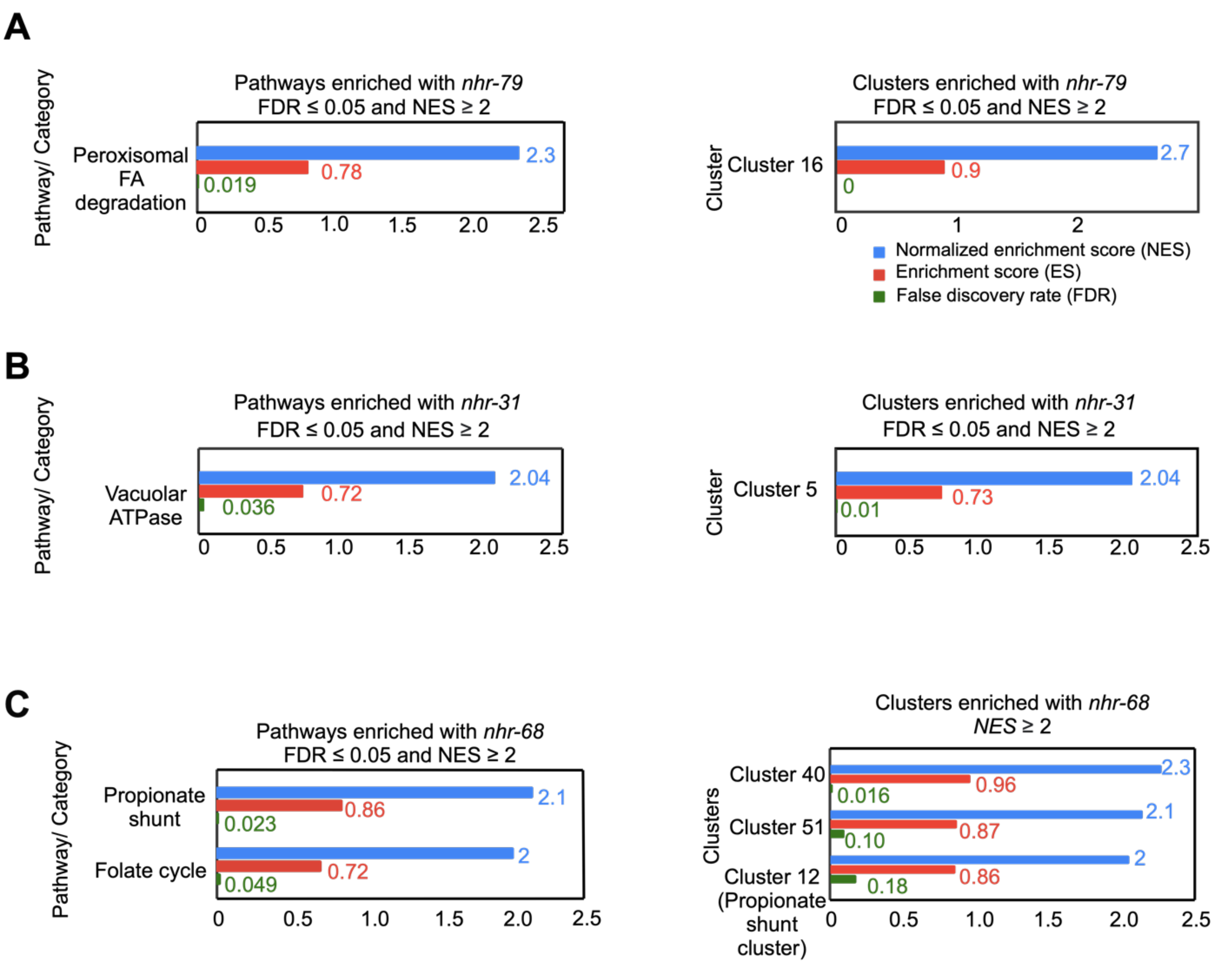

Appendix Figure S5. Enrichment of TFs to (Sub-)Pathways

(A) Plot showing enrichment to coexpression of *nhr-79* with peroxisomal fatty acid degradation and cluster 16 (NES≥2, FDR≤0.05).

(B) Plot showing enrichment to coexpression of *nhr-31* with vacuolar ATPases and cluster 5(NES≥2, FDR≤0.05).

(C) Plot showing enrichment to coexpression of *nhr-68* with propionate shunt (NES≥2, FDR≤0.05) and cluster 12(NES≥2).
