# Supplementary material for: Systems‐level transcriptional regulation of Caenorhabditis elegans metabolism
Source: Mol Syst Biol. 2023 Mar 21;19(5):e11443. doi: 10.15252/msb.202211443 (PMC10167481; doi:10.15252/msb.202211443)
Supplement: Supplementary file 15 [file MSB-19-e11443-s010.pdf]

molecular systems biology

Systems-level transcriptional regulation of Caenorhabditis elegans metabolism

Albertha Walhout, Shivani Nanda, Marc-Antoine Jacques, Wen Wang, Chad Myers, and Safak Yilmaz
DOI: 10.15252/msb.202211443

Corresponding author(s): Albertha Walhout (marian.walhout@umassmed.edu)

Review Timeline:

Submission Date:	7th Nov 22
Editorial Decision:	28th Nov 22
Revision Received:	22nd Feb 23
Editorial Decision:	2nd Mar 23
Revision Received:	3rd Mar 23
Accepted:	7th Mar 23

Editor: Maria Polychronidou

Transaction Report:

(Note: With the exception of the correction of typographical or spelling errors that could be a source of ambiguity, letters and reports are not edited. Depending on transfer agreements, referee reports obtained elsewhere may or may not be included in this compilation. Referee reports are anonymous unless the Referee chooses to sign their reports.)

1st Editorial Decision 28th Nov 2022

Thank you again for submitting your work to Molecular Systems Biology. We have now heard back from the three reviewers who agreed to evaluate your study. As you will see below, the reviewers think that the study presents a relevant resource. However, they raise a series of concerns, which we would ask you to address in a revision.

The recommendations of the reviewers are quite clear and I therefore see no need to repeat any of them here. Of note, a concern raised by all the reviewers is that the manuscript should be carefully rewritten and streamlined (e.g. simplifying some figures and moving methodological details to the Materials and Methods) so that it is easily accessible to the readers. The novel insights should be clearly described and contextualized with respect to the existing literature. All issues raised by the referees would need to be satisfactorily addressed. Please let me know in case you would like to discuss in further detail any of the issues raised, I would be happy to schedule a call.

Reviewer #1:

Metabolic pathways in a given organism are not static but can be regulated developmentally or in response to the environment. This manuscript from the Walhout lab uses global approaches to explore the transcriptional regulation of metabolic genes in the major model system C. elegans and relate this to their functions in metabolic pathways. This builds on their admirable previous work, which cataloged C. elegans metabolic genes and defined functional influences of metabolism on numerous phenotypes. They identify variably expressed metabolic genes in three distinct gene expression datasets using heuristic thresholding approaches, and identify numerous interesting patterns. Perhaps of most general interest, they show that in some cases combining co-expression with pathway location can identify functionally meaningful sub-pathways. An easy-to-use web tool was generated to allow the community to find pathways coexpressed with their genes of interest. Overall, I am supportive as I think this study should be of broad interest both as a C. elegans resource, and to help develop a paradigm for thinking about metabolic gene coregulation in other species.

My main suggestion is to try to make the results a bit less methods-heavy. While this is discretional, I think if some of the methodological details could be moved to the methods section and replaced by more conceptual explanations in the results, this might help increase impact/comprehension for a wider audience. The sections describing figures 3 and 4 are especially dense.

In Figure 1, the cutoffs used for categorization seem a bit empirical/arbitrary. I don't have a big problem with this, since it does seem a reasonable way to identify genes with the highest vs lowest variability. But I caution the authors to avoid overinterpreting these discretized categories. For example on page 8 when they argue "These results show that at least twice as many metabolic genes are variant and, therefore, likely transcriptionally regulated in different tissues than during development," I would argue this could instead be an artifact of the use of different datasets and different metrics/thresholds to identify variable genes in these two datasets. Arguments based on distributions of the actual data (such as in the scatter plot in Figure 1L) would be more compelling.

Minor suggestions

Page 10 the discussion of gene categories enriched in "Q1, "Q2" etc would be easier to follow with a reminder (or renaming of Q1/Q2 etc) of what the quadrants represent."

P13 "Such pathways may either not be regulated at all, may be regulated by allostery, or only one or a few genes in these pathways are transcriptionally regulated and may therefore function as key regulatory genes" - it is also possible they are coregulated in other conditions beyond those prevalent in the compendium.

Figure 2J - I suggest putting the pathway name on the figure at the top e.g. "All MET/SAM cycle pathway genes"

Figure EV4F/G while the color scale is presumably the same as in EV4C/DE, I suggest putting a legend on these panels as well.

Color scales in Figure 3B, 3D should be explicitly labeled (I think this is the coflux/expression score?)

Similarly, in Figure 3D, Figure 4 - color scale for gene names vs cluster ID should be explained with a legend on the plot

Does the compendium dataset include the stage and tissue datasets? Given the analysis in figures 1 and 6, a combined dataset might be most informative?

Figure 6F's "one outlier sample" suggests that variables beyond those intentionally included in the compendium experiments might influence co-expression. For example, it is possible that sample was contaminated or otherwise in a different nutritional state. To me this is a feature, not a bug and might be mentioned as such.

Related to that, while I hesitate to suggest more analyses given that the paper is already quite substantial, I did wonder for the analysis in Figure 6 whether comparing expression between datasets (As opposed to just within datasets) might identify additional patterns.

Reviewer #2:

The manuscript by Nanda et al. reports a large-scale analysis of gene expression across development and tissues in model organism C. elgans. The authors used previously published datasets to identify the degree of variability in the expression of genes coding for metabolic enzymes, assess co-expression and concordance with the known metabolic network structure (pathways and flux-coupling). The main conclusion is that a large proportion of metabolic genes are variable in time/space and that variation across tissues is higher than that during development. The authors postulate that similar approach would be applicable to other organisms including humans.

The study is (to my knowledge) the first to analyse metabolic gene expression variation in both space and time in the context of whole organism. Overall, I find the study of broad interest and the conclusions underline the importance of metabolic variation in development and function at whole organism level. Specific examples such as that of propionic acid metabolism will be also useful for the C. elegans community.

While I do not have any technical concerns, there are several points where the manuscript needs improvement towards improving the accuracy of the conclusions.

1. Higher variation across space (tissues) than time (development): This is quite puzzling since development leads to spatial variation (differentiation into different tissues). Thus, this conclusion is likely an artefact of the lack of resolution (cell level and timing) in the developmental dataset. The manuscript needs to clearly discuss this limitation and explain the results in the biological context of development.

2. "the extent of transcriptional regulation of the entire metabolic network remains largely unknown": this and other similar statements need qualifying since regulation at network level has been extensively studies in microbes like yeast and E. coli etc.

3. "metabolism is precisely controlled"   I don't think there is any evidence supporting that the control is "precise".

4. Page 6, 2nd paraph: the reference to the original study should come earlier.

5. Page 10: please define Q1-Q4 also in the text to. make it easier to read.

6. Analysis based on flux coupling (starting Page 15): How can flux coupling-based analysis be unsupervised?? Definition of flux couplings requires a model that is reconstructed using prior data and manual curation.

7. The number of figures feels too many, especially that many of these are very descriptive. Also, the description of the web application could be much shortened. The users can explore it themselves.

Reviewer #3:

The manuscript by Nanda et al. investigates gene co-expression in the model nematode C. elegans with a focus on metabolism. In this computational project, they used published transcriptomics data and an established metabolic model to identify subsets of co-regulated genes involved in metabolism. They further make the data easily accessible through a web app. The web app and making the data available is potentially quite valuable but we are left wondering what the scientific insights of the presented manuscript are? Overall, the rather long manuscript describes analysis steps in great detail and lists many descriptive examples of individual observations without extracting principles, systems-level insights, or at least connecting previously not connected reactions/genes to the network. Although we started to read into the present lengthy version of the manuscript with great interest, it was hard to continue after a few pages because of insight-poor lists of seemingly arbitrary examples (a particular tedious section are pages 15-22 that could be dealt with on a single page).
This is not to say that this work should not be published, but to render it a relevant contribution - beyond the web app - the authors should decide on what is really new and important and focus their writing on that. A third of the presently used text would be fully sufficient for that.

Major points:
1. Throughout, the main reported observations are individual examples of gene and pathway coregulation without providing any larger picture or at least making clear why the reported examples are relevant or informative. In fact, several examples appear to have been selected primarily to boost self-citation such as the propionate pathway (p 11/11 and elsewhere). Consistently the manuscript stops at the examples and shies away from looking into the metabolic genes (25% of all), gene categories or pathways that are not regulated at all. It is hard to find novelty or gain insights by only looking into things that can easily be explained.

The only general conclusion we could find was the indication that about 3/4 of the genes are transcriptionally regulated, which does not seem to be particularly novel. Akin to most other observations, it is not made clear how this compares to other organisms or in general our present view of transcriptional regulation.
2. To compute their final metric to assess co-expression, the authors give the same weight to (i) their newly calculated co-

expression values across the set of reanalyzed published transcriptomics data and (ii) coflux values obtained from a published metabolic model. Although there is probably some overlap to both aspects, the metric reflects both at the same time and it is unclear whether what results of it can be considered co-expression. Particularly, figure 3b raises the question of what is the contribution of each side to it in the clusters' delineation? Visually, it seems like similar clusters to the ones later selected for comments (ex fig. 5) could also have been identified in the coflux matrix alone, but not in the co-expression one, suggesting they mostly arise from the coflux data. It would be beneficial to look at what is the result of the clustering analysis on each individual matrix and how the called clusters differ between the three. Depending on the results, it might be worth considering using other terms for the obtained data, especially if what drives the delineation of clusters used throughout the paper comes mostly from the coflux data and not the co-expression data. Different claims would have to be accordingly adjusted throughout the paper.
3. Generally, the manuscript does not deal well with the existing literature, which makes it even harder to appreciate any novelty. For example, previous studies of co-expression analyses in C. elegans and delineation of co-expressed clusters of genes across different datasets are not discussed. Similarly, we don't think it is a fair starting point to argue that transcriptional regulation of metabolism has only been studied for individual genes and enzymes (end of 1st intro paragraph). There are quite a few of those, for example from the Julio Collado-Vides and Palsson labs to just mention two microbial examples. We are not saying everything is done but the relevance of the here reported contributions could be much better appreciated if the novelty is made explicit relative to other systems-level work. Moreover, to validate their approach and strengthen some of the claims, the authors should consider comparing the clusters they identified as co-expressed with the known regulatory network of C. elegans and the sets of genes regulated by each characterized TF.
4. A particular aspect of C. elegans as an animal is that it still has genes organized in operons, which provides an ideal benchmark for co-expression analyses. The authors should use it to (i) validate their approach and (ii) assess what proportion of the results are explained by it, which could also provide additional mechanistic insights.

Minor points:
1. The authors use different measures of variation between their three datasets. They then further use these different metrics to compare variation across datasets (CV across tissues vs. VS across development) and delineate groups of genes based on the comparison. As such, it is unclear if the definition of the 4 different groups is solely affected by the underlying biology or if the difference in variation metric also plays a role. While one measure can only be obtained on one of the dataset, the other could be applied to all three and alleviate that concern.
2. P13 line 4: an observation cannot be validated .....
3. P5 top: we agree that the inclusion of not yet connected reactions would be nice for genome-scale models, but this manuscript does not contribute anything to this problem.

- We thank reviewers for their thorough review and helpful comments and suggestions. We respond to their comments in blue below. We edited the paper and added further analyses as necessary.
- We have streamlined the paper and restructured the main text while moving the details of methods to the 'Materials and Methods' section, based on the editor's and reviewers' comments.
- As requested by the Editor, we added a Structured Methods section.
- Tables EV1-EV11 have been provided as Datasets EV1-EV11
- We have extended the Introduction to include more related studies in other organisms as well as the gaps that motivated us to do our study.
- We have modified the Discussion section to address reviewers' comments and highlight scientific insights.

Reviewer #1:
Metabolic pathways in a given organism are not static but can be regulated developmentally or in response to the environment. This manuscript from the Walhout lab uses global approaches to explore the transcriptional regulation of metabolic genes in the major model system C. elegans and relate this to their functions in metabolic pathways. This builds on their admirable previous work, which cataloged C. elegans metabolic genes and defined functional influences of metabolism on numerous phenotypes. They identify variably expressed metabolic genes in three distinct gene expression datasets using heuristic thresholding approaches and identify numerous interesting patterns. Perhaps of most general interest, they show that in some cases combining co-expression with pathway location can identify functionally meaningful sub-pathways. An easy-to-use web tool was generated to allow the community to find pathways coexpressed with their genes of interest. Overall, I am supportive as I think this study should be of broad interest both as a C. elegans resource, and to help develop a paradigm for thinking about metabolic gene coregulation in other species.'

My main suggestion is to try to make the results a bit less methods-heavy. While this is discretional, I think if some of the methodological details could be moved to the methods section and replaced by more conceptual explanations in the results, this might help increase impact/comprehension for a wider audience. The sections describing figures 3 and 4 are especially dense.

We thank the reviewer for the constructive and supportive comments. We have streamlined the paper and restructured the main text and have moved some details of methods to the 'materials and methods' section. The sections describing **Figures 3** and **4** have been made more concise to enable better readability for a broad audience.

In Figure 1, the cutoffs used for categorization seem a bit empirical/arbitrary. I don't have a big problem with this, since it does seem a reasonable way to identify genes with the highest vs lowest variability. But I caution the authors to avoid

overinterpreting these discretized categories. For example, on page 8 when they argue "These results show that at least twice as many metabolic genes are variant and, therefore, likely transcriptionally regulated in different tissues than during development," I would argue this could instead be an artifact of the use of different datasets and different metrics/thresholds to identify variable genes in these two datasets. Arguments based on distributions of the actual data (such as in the scatter plot in Figure 1L) would be more compelling.

We thank the reviewer for this valuable comment. We compared the two datasets using the same metric (CV) and threshold (CV≥0.75) and found that percentage of highly variant genes across development lowered from 31% to 15%, supporting our conclusion. We have added the following to the Results of the revised manuscript (page 9 lines 189-200):

*"Overall, metabolic gene expression showed higher variation across space (tissues) than time (development). However, because we used two different statistical methods for the development and tissue datasets, we confirmed that it held true when we applied the same CV measure we used in the tissue dataset to the development dataset (**Appendix Fig S1A**). The two datasets also have different resolution: the development dataset has great temporal but no spatial resolution because it was measured by bulk RNA-seq while the tissue dataset, which was measured by single cell RNA-seq, has great spatial but no temporal resolution. Therefore, we examined genes that are highly tissue-specific, because they are highly expressed in a single tissue in the tissue dataset, and found that only 57% of these are also highly variant in the development dataset (**Appendix Fig S1B**). Therefore, we conclude that transcriptional regulation of metabolic genes more frequently establishes spatial than temporal gene expression patterns".*

Minor suggestions
Page 10 the discussion of gene categories enriched in "Q1, "Q2" etc would be easier to follow with a reminder (or renaming of Q1/Q2 etc) of what the quadrants represent."

We thank the reviewer for this helpful comment. We have revised the text as follows (pages 9-10 lines 201-227):

"*To directly compare metabolic gene expression in tissues and development, we plotted VS values of metabolic genes across development versus CV values across tissues and found that these two parameters are moderately correlated (**Fig 1E**). We divided the scatter plot into four quadrants, based on the thresholds used in each dataset (**Dataset EV4**). To determine if there are any functional enrichments, we performed pathway enrichment analysis (PEA) on the metabolic genes for each quadrant using the tool provided on the WormFlux website {Yilmaz, 2016 #3236}. The first quadrant (Q1) consists of genes with moderate/low developmental variation and moderate/low tissue variation. It has 595 metabolic genes, including 385 iCEL1314 genes that are enriched in several metabolic pathways, such as the*

*electron transport chain (ETC), aminoacyl-tRNA biosynthesis, the tricarboxylic acid (TCA) cycle, the pentose phosphate pathway and glycolysis/ gluconeogenesis (**Appendix Fig S2A**). The second quadrant (Q2), with high developmental variation and moderate/low tissue variation, consists of only 176 genes, including 68 iCEL1314 genes that are enriched in sulfur, cysteine, and methionine metabolism (**Appendix Fig S2A**). The 891 genes in the third quadrant (Q3) consist of genes with moderate/low developmental variation and high tissue variation. They include 504 iCEL1314 genes that are highly enriched in lipid metabolism. Notably, genes involved in peroxisomal fatty acid (FA) metabolism vary more in expression than mitochondrial FA degradation (**Appendix Fig S2A**). Finally, the 577 genes in the fourth quadrant (Q4) show high developmental variation and high tissue variation. They include 261 iCEL1314 genes which are enriched in UDP-glucuronosyltransferases (UGT) enzymes, guanylate cyclases, glyoxylate and dicarboxylate metabolism, and amino acid metabolism, such as arginine and proline metabolism and glutamate/ glutamine metabolism (**Appendix Fig S2A**)*".

P13 "Such pathways may either not be regulated at all, may be regulated by allostery, or only one or a few genes in these pathways are transcriptionally regulated and may therefore function as key regulatory genes" - it is also possible they are coregulated in other conditions beyond those prevalent in the compendium.

We agree and have revised the manuscript to include this statement as follows (page 13 lines 285-286):

"*Alternatively, these pathways maybe coregulated in conditions that were not yet profiled and therefore are not included in the compendium.".*

Figure 2J - I suggest putting the pathway name on the figure at the top e.g. "All MET/SAM cycle pathway genes"

We have made the change.

Figure EV4F/G while the color scale is presumably the same as in EV4C/DE, I suggest putting a legend on these panels as well.

We have changed the numbering and order of figures to accommodate other analysis. We have put the color panels on figures EV4A and EV4D and indicated this in the figure legend.

Color scales in Figure 3B, 3D should be explicitly labeled (I think this is the coflux/expression score?)

The color scales for coflux, coexpression, and the product of coflux coexpression are the same, and hence the color bar has been labeled accordingly. **Fig 3D** has changed to **Fig 3C** in the revised manuscript. The figure legend for **Fig 3C** has been appended with the statement "*Color legend as indicated in (B)*".

Similarly, in Figure 3D, Figure 4 - color scale for gene names vs cluster ID should be explained with a legend on the plot

We moved the color bar to **4A** and indicated in the legend that this color bar applies to all panels in **Figure 4**.

Does the compendium dataset include the stage and tissue datasets? Given the analysis in figures 1 and 6, a combined dataset might be most informative?

The compendium dataset does not include the development and tissue datasets. We designed our study to keep the variation due to environmental conditions separate from space and time.

Figure 6F's "one outlier sample" suggests that variables beyond those intentionally included in the compendium experiments might influence co-expression. For example, it is possible that sample was contaminated or otherwise in a different nutritional state. To me this is a feature, not a bug and might be mentioned as such.

We thank the reviewer for this insightful comment. We have revised the manuscript as follows (page 24 lines 530-532):

"*It is however also possible that this outlier sample was unknowingly contaminated to change the nutritional or environmental state, hence driving the variable expression of these genes (**Fig 6F**)*".

Related to that, while I hesitate to suggest more analyses given that the paper is already quite substantial, I did wonder for the analysis in Figure 6 whether comparing expression between datasets (As opposed to just within datasets) might identify additional patterns.

We thank the reviewer for this comment. When we looked at the global heatmap of mean coexpression of clusters in all the datasets, there was no interesting or apparent inter-dataset pattern (as can be seen in **Fig. 6A**). There can be multiple combinations of datasets that can give interesting insights, which are difficult to predict.

Reviewer #2:
The manuscript by Nanda et al. reports a large-scale analysis of gene expression across development and tissues in model organism *C. elegans*. The authors used previously published datasets to identify the degree of variability in the expression of genes coding for metabolic enzymes, assess co-expression and concordance with the known metabolic network structure (pathways and flux-coupling). The main conclusion is that a large proportion of metabolic genes are variable in time/space and that variation across tissues is higher than that during development. The authors postulate that similar approach would be applicable to other organisms including

humans.

The study is (to my knowledge) the first to analyse metabolic gene expression variation in both space and time in the context of whole organism. Overall, I find the study of broad interest and the conclusions underline the importance of metabolic variation in development and function at whole organism level. Specific examples such as that of propionic acid metabolism will be also useful for the C. elegans community.

While I do not have any technical concerns, there are several points where the manuscript needs improvement towards improving the accuracy of the conclusions.

1. Higher variation across space (tissues) than time (development): This is quite puzzling since development leads to spatial variation (differentiation into different tissues). Thus, this conclusion is likely an artefact of the lack of resolution (cell level and timing) in the developmental dataset. The manuscript needs to clearly discuss this limitation and explain the results in the biological context of development.

We thank the reviewer for this valuable comment, which is similar to the point raised by other reviewers. We think this is not a major concern because we used a postembryonic development dataset and that the body plan with all major tissues has already been established at that stage. In addition, we found that only 57% of the tissue-specific metabolic genes are highly variant in development, further showing that only around half of the highly variable genes in tissues are transcriptionally regulated across development. This analysis has also been added to the Results section of the manuscript as (page 9 lines 193-200):

*"The two datasets also have different resolution: the development dataset has great temporal but no spatial resolution because it was measured by bulk RNA-seq while the tissue dataset, which was measured by single cell RNA-seq, has great spatial but no temporal resolution. Therefore, we examined genes that are highly tissue-specific, because they are highly expressed in a single tissue in the tissue dataset, and found that only 57% of these are also highly variant in the development dataset (**Appendix Fig S1B**). Therefore, we conclude that transcriptional regulation of metabolic genes more frequently establishes spatial than temporal gene expression patterns."*

2. "the extent of transcriptional regulation of the entire metabolic network remains largely unknown": this and other similar statements need qualifying since regulation at network level has been extensively studied in microbes like yeast and E. coli etc.

We agree with the reviewer and apologize for the oversight. We have made revisions throughout the manuscript to reference studies in single cell organisms.

3. "metabolism is precisely controlled". I don't think there is any evidence supporting that the control is "precise".

We have changed "*Metabolism is precisely controlled*" has been changed to "*Metabolism is controlled*" in the Abstract of the revised manuscript.

4. Page 6, 2nd paraph: the reference to the original study should come earlier.

We moved the positioning of this reference up as per the reviewer's suggestion.

5. Page 10: please define Q1-Q4 also in the text to make it easier to read.

A similar comment has been raised by reviewer 1. See above for our response.

6. Analysis based on flux coupling (starting Page 15): How can flux coupling-based analysis be unsupervised?? Definition of flux couplings requires a model that is reconstructed using prior data and manual curation.

The approaches have been defined as 'supervised' and 'unsupervised' based on the statistical methods used. We call the first approach 'supervised' since we use a gene set enrichment analysis (GSEA)-based approach on the already annotated WormPaths pathways. Whereas, in the second approach, we do not define the clusters prior to the analysis and only perform hierarchical clustering(unsupervised) based on the coflux(flux-coupling) and coexpression values of different genes. However, we do understand the reviewer's point of view regarding the use of the reconstructed metabolic network to establish these clusters or pathway boundaries. Therefore, we have changed the definition of the approach to 'semi-supervised'.

7. The number of figures feels too many, especially that many of these are very descriptive. Also, the description of the web application could be much shortened. The users can explore it themselves.

We do believe that we need all the main figures for the readers to understand and appreciate our paper. We have provided expanded view figures and supplementary figures firstly for the completeness of analyses and secondly for readers who would be interested in additional data and details. However, we have streamlined the Methods and Results sections. **Figure 7**, which discusses the tool WormClust also shows validated results, illuminating its utility.

Reviewer #3:
The manuscript by Nanda et al. investigates gene co-expression in the model nematode *C. elegans* with a focus on metabolism. In this computational project, they used published transcriptomics data and an established metabolic model to identify subsets of co-regulated genes involved in metabolism. They further make the data easily accessible through a web app. The web app and making the data available is potentially quite valuable but we are left wondering what the scientific insights of the presented manuscript are? Overall, the rather long manuscript describes analysis steps in great detail and lists many descriptive examples of individual observations

without extracting principles, systems-level insights, or at least connecting previously not connected reactions/genes to the network. Although we started to read into the present lengthy version of the manuscript with great interest, it was hard to continue after a few pages because of insight-poor lists of seemingly arbitrary examples (a particular tedious section are pages 15-22 that could be dealt with on a single page). This is not to say that this work should not be published, but to render it a relevant contribution - beyond the web app - the authors should decide on what is really new and important and focus their writing on that. A third of the presently used text would be fully sufficient for that.

We respectfully disagree with the reviewer's comments that text on pages 15-22 can be simplified on one page. However, we have streamlined the Methods and Results sections in the revised manuscript.

Major points:
1. Throughout, the main reported observations are individual examples of gene and pathway coregulation without providing any larger picture or at least making clear why the reported examples are relevant or informative. In fact, several examples appear to have been selected primarily to boost self-citation such as the propionate pathway (p 11/11 and elsewhere).

We use the propionate shunt pathway simply because we know most about it and it provided a good benchmark for our analyses (Watson *et al.,* 2016, Bulcha *et al.*, 2019). Some other examples like Met/SAM cycle also serve this purpose.

Consistently the manuscript stops at the examples and shies away from looking into the metabolic genes (25% of all), gene categories or pathways that are not regulated at all. It is hard to find novelty or gain insights by only looking into things that can easily be explained.

We thank the reviewer for bringing up this interesting point. We already looked at the pathways enriched in genes that are not highly variant across both development as well as tissues, with a quadrant analysis in the original manuscript. In addition, we now found that 25% of all metabolic genes, that are not highly variant, are enriched in the similar pathways as Q1. We have also included a new phenotypic enrichment analysis to learn more about these 25% of genes. With this analysis, we found that these moderately variant genes are mainly enriched in essential phenotypes such as larval arrest, slow growth and sterility. To further support this conclusion, we also found that in contrast to the 25% moderately variant genes, highly variant genes are depleted in these essential phenotypes and enriched in conditional response variants. The new analysis is included in the revised manuscript (**Appendix Figs S1C and S1D**). This analysis is described in the revised manuscript as follows: (page 11 lines 241-247*)*

"Using phenotypes provided in WormBase WS282 {Harris, 2020 #3718}, we found that the 75% highly variant metabolic genes are enriched in conditional response

*variants such as chemical and pathogen response, and depleted in essential phenotypes such as lethality, larval arrest, slow growth, and sterility (**Appendix Fig S2B**). Finally, the remaining 624 (25%) of metabolic genes that are not highly variant in any dataset are similar in pathway enrichment as the Q1 genes discussed above and are enriched in the essential phenotypes (**Appendix Fig S2C-D**)."*

The only general conclusion we could find was the indication that about 3/4 of the genes are transcriptionally regulated, which does not seem to be particularly novel. Akin to most other observations, it is not made clear how this compares to other organisms or in general our present view of transcriptional regulation.

We revised the manuscript to include studies from single cell organisms such as *E. coli* and yeast and included the conclusion that the large degree of transcriptional regulation of metabolic pathways is evolutionarily conserved (pages 26-27 lines 588-595). However, none of the published studies quantified the overall extent of transcriptional regulation of metabolism across space, time and conditions.

In addition to this general finding, we identify which pathways exhibit coexpression, define coexpressed sub-pathways/clusters, identify pathway boundaries, and defines the datasets (conditions) in which pathways/clusters are most greatly regulated.

2. To compute their final metric to assess co-expression, the authors give the same weight to (i) their newly calculated co-expression values across the set of reanalyzed published transcriptomics data and (ii) coflux values obtained from a published metabolic model. Although there is probably some overlap to both aspects, the metric reflects both at the same time and it is unclear whether what results of it can be considered co-expression. Particularly, figure 3b raises the question of what is the contribution of each side to it in the clusters' delineation? Visually, it seems like similar clusters to the ones later selected for comments (ex fig. 5) could also have been identified in the coflux matrix alone, but not in the co-expression one, suggesting they mostly arise from the coflux data. It would be beneficial to look at what is the result of the clustering analysis on each individual matrix and how the called clusters differ between the three. Depending on the results, it might be worth considering using other terms for the obtained data, especially if what drives the delineation of clusters used throughout the paper comes mostly from the coflux data and not the co-expression data. Different claims would have to be accordingly adjusted throughout the paper.

We thank the reviewer for this important comment. Briefly, we used coflux in addition to coexpression to obtain sets of coexpressed genes that function in connected reactions in the model. This approach provides a way to analyze coregulated metabolic genes that likely function together without the bias of prior pathway annotations. We are therefore not concerned with the individual contribution of each matrix but are most interested in clusters obtained by multiplying these matrices and are hence both interconnected and coexpressed, as the product cannot be large if

either of these does not contribute. We have modified and streamlined our description of coflux and product matrix in the Results section of the revised manuscript as follows (pages 15-16 lines 338-352):

"*Our finding that metabolic pathways and categories exhibit extensive coexpression was based on previously annotated pathways {Walker, 2021 #4026}. However, these pathways connect into the larger metabolic network and the definition of the start and ending of each pathway is somewhat arbitrary. Since there is extensive coexpression of genes that function together in pre-defined pathways, we reasoned that we may be able to use coexpression to extract metabolic (sub)-pathways in an unbiased manner. To specifically focus on metabolic genes that function in connected reactions in the metabolic network, we developed a 'coflux' metric that calculates flux dependency between metabolic genes using the network model (see details in* ***Methods****) (****Dataset EV8****). Reactions in linear pathways have complete flux dependence, (i.e., coflux = 1), while in branched pathways flux dependency may be partial (coflux = between 0 and 1), and in uncoupled reactions there is no dependence (coflux=0). We then used a custom semi-supervised approach that multiplies coflux and coexpression values, and clustered the resulting product matrix with a relatively stringent set of parameters (****Figs 3A and 3B, Dataset EV9****,* ***Appendix Fig S4A****, see* ***Methods*** *for details).*"

To answer the reviewer's questions more specifically, even though many genes that are part of connected reactions or pathways are coexpressed, these results could not have been obtained by using the coflux matrix alone. A prominent example of this is one of the top clusters that captures the entire peroxisomal fatty acid degradation genes. Only *acox-1.1* and *acox-3* out of seven *acox* family genes are coexpressed with the other peroxisomal FA oxidation genes, indicating that these two genes are more likely to function in this pathway than the other *acox* genes. The other acox genes are coexpressed with each other, and with mitochondrial FA degradation genes. If this clustering were only influenced by coflux, all the other acox genes would have clustered with the peroxisomal fatty acid degradation cluster.

However, when it comes to visually extracting communities, we agree with the reviewer's criticism and have removed the third community (5D), that did not show very high coexpression. The new communities 5B, 5C and 5D, all show high coexpression.

3. Generally, the manuscript does not deal well with the existing literature, which makes it even harder to appreciate any novelty. For example, previous studies of co-expression analyses in C. elegans and delineation of co-expressed clusters of genes across different datasets are not discussed. Similarly, we don't think it is a fair starting point to argue that transcriptional regulation of metabolism has only been studied for individual genes and enzymes (end of 1st intro paragraph). There are quite a few of those, for example from the Julio Collado-Vides and Palsson labs to just mention two microbial examples. We are not saying everything is done but the

relevance of the here reported contributions could be much better appreciated if the novelty is made explicit relative to other systems-level work.

We apologize and agree that systems-level studies have been done in unicellular organisms and, to a lesser degree, in plants before. However, our study is a first-of-its-kind in animals. As stated above, we have revised our manuscript to better reflect this as follows (page 4 lines 76-81):

"*The contribution of transcriptional regulation of metabolism has mostly been studied at a systems, or network, level, in single cell organisms such as E. coli and S. cerevisiae, and to a lesser extent in plants {Seshasayee, 2009 #4199}{Ledezma-Tejeida, 2017 #4200}{Ihmels, 2004 #4196}{Kharchenko, 2005 #4197}{Tang, 2021 #4198}. However, the extent to which overall metabolic activity is under transcriptional control in animals remains unclear.* "

We also discuss previous studies of coexpression analysis in *C. elegans* and delineation of co-expressed clusters of genes in the revised manuscript as follows (page 5 lines 94-100):

"Guilt-by-association is a powerful concept in systems biology that can be used to identify genes with shared functions. One way this can be done is by coexpression analysis where a functional association can be predicted when genes are coexpressed in many transcriptomic datasets {Stuart, 2003 #1006;Eisen, 1998 #223;Hughes, 2000 #436;Kim, 2001 #441;Segal, 2003 #1145}. In C. elegans, coexpression analysis has been used to study global, temporal, and spatial gene expression {Kim, 2001 #441}{Reinke, 2000 #366}{Spencer, 2011 #2421}{Kim, 2016 #4201}{Liu, 2018 #4202}. ".

Moreover, to validate their approach and strengthen some of the claims, the authors should consider comparing the clusters they identified as co-expressed with the known regulatory network of C. elegans and the sets of genes regulated by each characterized TF.

In our original submission, we already validated the coexpression of the TFs, *nhr-68*, *nhr-31,* and *nhr-79* to the regulation of the propionate shunt, vacuolar ATPases, and peroxisomal fatty acid degradation, respectively (**Figs 7B-7D**). In the revised manuscript, we now examined coexpression of these TFs with clusters or (sub)pathways, the results of which have been added to the revised manuscript as follows (pages 25-26 line 564-573):

"*In addition to pathways, we also performed enrichment of clusters or sub-pathways from our semi-supervised analysis with TFs. We found that nhr-79 is enriched to cluster 16 (stringent), which contains of peroxisomal FA degradation genes; nhr-31 shows enrichment to cluster 5 (relaxed) that consists of vacuolar ATPases; and nhr-68 shows enrichment to cluster 12 (stringent) which contains propionate shunt genes, albeit with higher FDR. In addition, nhr-68 shows enrichment to cluster 40*

*consisting of mans-2, hex-2 and fut-8 (N-glycan biosynthesis), and cluster 51 consisting of bgal-1, gana-1 (galactose metabolism) and hex-1 (sphingolipid metabolism)(**Appendix Fig S5A-C, Dataset EV9**). This observation suggests that nhr-68 may play a broader role in the regulation of metabolic gene expression."*

4. A particular aspect of C. elegans as an animal is that it still has genes organized in operons, which provides an ideal benchmark for co-expression analyses. The authors should use it to (i) validate their approach and (ii) assess what proportion of the results are explained by it, which could also provide additional mechanistic insights.

We thank the reviewer for this fantastic suggestion – we did not think of this! In the revised manuscript, we compared the coexpression of metabolic gene pairs existing in the same operon with random metabolic gene pairs. We found that genes in operons are indeed more coexpressed than random genes. We compared the coexpression of all metabolic operon genes, pathway genes as well as PO genes (pathway genes excluding operon genes). We found that the contribution of operon gene coexpression is insignificant in pathway gene expression, mainly because there are very few known pairs of metabolic genes in operons. This analysis has been included in the revised manuscript (pages 14-15 lines 314-335):

*"In C. elegans, ~18% of genes are transcribed from operons {Blumenthal, 2002 #566}. In total, 26% of metabolic genes occur in operons. However, they most frequently occur as a pair with a non-metabolic gene. In total, 242 metabolic genes (~10% of all metabolic genes) occur in a pair with another metabolic gene in an operon (**Dataset S1**). As expected, these operon gene pairs are more coexpressed than random gene pairs, OR genes and other paralogs, thus, serving as a validation for our coexpression analysis. However, these pairs are less coexpressed than AND genes and there is no overlap between the two categories. This shows that enzyme complexes are strongly coregulated and their coregulation mechanism is largely independent of operonic organization (**Figs 2K, EV4C, EV4D, and EV4E**).*

*Based on the analysis of AND, OR and operon genes, it is difficult to determine the contribution of the coexpression of such gene pairs to pathway enrichment. Therefore, we examined coexpression of gene pairs that are annotated with distinct reactions in a pathway, which we refer to as pathway (PW) genes (**Fig 2J**). We found that PW gene pairs are significantly more coexpressed than random gene pairs (**Figs 2L, EV4F, EV4G, and EV4H**). We also examined coexpression of gene pairs that are not part of an operon, which we refer to as pathway excluding operon (PO) genes. There are only three pathway gene pairs that are part of operon, hence there is no significant difference between pathway genes and PO genes coexpression (**Figs 2L, EV4F, EV4G, and EV4H**). Therefore, pathway coexpression is not just driven by AND, OR and operon genes, indicating that pathway genes' coexpression is a true feature of many metabolic pathways."*

Minor points:

1. The authors use different measures of variation between their three datasets. They then further use these different metrics to compare variation across datasets (CV across tissues vs. VS across development) and delineate groups of genes based on the comparison. As such, it is unclear if the definition of the 4 different groups is solely affected by the underlying biology or if the difference in variation metric also plays a role. While one measure can only be obtained on one of the datasets, the other could be applied to all three and alleviate that concern.

A similar comment has been raised by other reviewers and has been addressed above.

2. P13 line 4: an observation cannot be validated .....

We agree with the reviewer's comment, and we have rephrased this line to "*With an FDR cutoff of ≤ 0.05, 52 of 84 metabolic pathways or categories (~61%) exhibit coexpression, which is significantly more than expected by chance (**Fig 2B, 2C, Dataset EV7, Appendix Fig S3**).*" in the revised manuscript (page 12 lines 270-273).

3. P5 top: we agree that the inclusion of not yet connected reactions would be nice for genome-scale models, but this manuscript does not contribute anything to this problem.

We have removed the relevant statement from the Introduction of the revised manuscript.

1st Revision - Editorial Decision 2nd Mar 2023

Thank you for sending us your revised manuscript. We have now heard back from the two reviewers who were asked to evaluate your revised study. As you will see below, both reviewers are satisfied with the performed revisions and support publication. Reviewer #1 is still somewhat concerned about the explanation regarding the transcriptional regulation of metabolic genes in relation to spatial and temporal gene expression patterns. Perhaps some further text modifications might be helpful, to avoid similar misunderstandings by readers.

Before we formally accept the manuscript for publication, we would ask you to address some remaining editorial issues listed below.

Reviewer #2:

I am not convinced by the response on development vs space distinction since it does not fit with the biological concept of development. Development is what leads to the tissue-level differences - how else authors explain the tissue-level differences, which by definition will be development unless authors are claiming some sort of post-developmental "drift" but that would need a whole new study to prove.

Regarding number of figures, I do not think descriptive figures add much but it is a matter of style.

Reviewer #3:

In my opinion the authors have done a decent job in addressing the raised issues and took care of the major points. All remaining disagreements are about style and are therefore ultimately author decisions. Hence, I support publication in MSB.

Reviewer #2:
I am not convinced by the response on development vs space distinction since it does not fit with the biological concept of development. Development is what leads to the tissue-level differences - how else authors explain the tissue-level differences, which by definition will be development unless authors are claiming some sort of post-developmental "drift" but that would need a whole new study to prove.

We believe reviewer's comment is with respect to the argument on rebuttal that states*: "We think this is not a major concern because we used a postembryonic development dataset and that the body plan with all major tissues has already been established at that stage."* While we did not include this argument in manuscript, the relevant comment as well as other similar ones were previously addressed by toning down the conclusion about spatial vs temporal variation and by additional analyses. We further clarify the conclusion by stating in main text that we are comparing larval development to tissue variation at a fixed time point (L2). This has been mentioned in the main text as follows (page 9, lines 189-201; modified sentence in main text is in red):

*"Overall, metabolic gene expression showed higher variation across tissues at a fixed time point (L2) than larval development. However, because we used two different statistical methods for the development and tissue datasets, we confirmed that it held true when we applied the same CV measure we used in the tissue dataset to the development dataset (**Appendix Fig S1A**). The two datasets also have different resolution: the development dataset has great temporal but no spatial resolution because it was measured by bulk RNA-seq while the tissue dataset, which was measured by single cell RNA-seq, has great spatial but no temporal resolution. Therefore, we examined genes that are highly tissue-specific, because they are highly expressed in a single tissue in the tissue dataset, and found that only 57% of these are also highly variant in the development dataset (**Appendix Fig S1B**). Therefore, we conclude that transcriptional regulation of metabolic genes more frequently establishes spatial than temporal gene expression patterns."*

2nd Revision - Editorial Decision **7th Mar 2023**

Thank you again for sending us your revised manuscript. We are now satisfied with the modifications made and I am pleased to inform you that your paper has been accepted for publication.

EMBO Press Author Checklist

Corresponding Author Name: Albertha JM Walhout	
Journal Submitted to: Molecular Systems Biology	
Manuscript Number: MSB-2022-11443	

USEFUL LINKS FOR COMPLETING THIS FORM
The EMBO Journal - Author Guidelines
EMBO Reports - Author Guidelines
Molecular Systems Biology - Author Guidelines
EMBO Molecular Medicine - Author Guidelines

Reporting Checklist for Life Science Articles (updated January
This checklist is adapted from Materials Design Analysis Reporting (MDAR) Checklist for Authors. MDAR establishes a minimum set of requirements in transparent reporting in the life sciences (see Statement of Task: 10.31222/osf.io/9sm4x). Please follow the journal's guidelines in preparing your
Please note that a copy of this checklist will be published alongside your article.

Abridged guidelines for figures
1. Data
The data shown in figures should satisfy the following conditions:
- the data were obtained and processed according to the field's best practice and are presented to reflect the results of the experiments in an accurate and unbiased manner.
- ideally, figure panels should include only measurements that are directly comparable to each other and obtained with the same assay.
- plots include clearly labeled error bars for independent experiments and sample sizes. Unless justified, error bars should not be shown for technical
- if n<5, the individual data points from each experiment should be plotted. Any statistical test employed should be justified.
- Source Data should be included to report the data underlying figures according to the guidelines set out in the authorship guidelines on Data

2. Captions
Each figure caption should contain the following information, for each panel where they are relevant:
- a specification of the experimental system investigated (eg cell line, species name).
- the assay(s) and method(s) used to carry out the reported observations and measurements.
- an explicit mention of the biological and chemical entity(ies) that are being measured.
- an explicit mention of the biological and chemical entity(ies) that are altered/varied/perturbed in a controlled manner.
- the exact sample size (n) for each experimental group/condition, given as a number, not a range;
- a description of the sample collection allowing the reader to understand whether the samples represent technical or biological replicates (including how many animals, litters, cultures, etc.).
- a statement of how many times the experiment shown was independently replicated in the laboratory.
- definitions of statistical methods and measures:
  - common tests, such as t-test (please specify whether paired vs. unpaired), simple $\chi 2$ tests, Wilcoxon and Mann-Whitney tests, can be unambiguously identified by name only, but more complex techniques should be described in the methods section;
  - are tests one-sided or two-sided?
  - are there adjustments for multiple comparisons?
  - exact statistical test results, e.g., P values = x but not P values < x;
  - definition of 'center values' as median or average;
  - definition of error bars as s.d. or s.e.m.

Please complete ALL of the questions below.
Select "Not Applicable" only when the requested information is not relevant for your study.

Materials

Newly Created Materials	Information included in the manuscript?	In which section is the information available? (Reagents and Tools Table, Materials and Methods, Figures, Data Availability Section)
New materials and reagents need to be available; do any restrictions apply?	Not Applicable	

Antibodies	Information included in the manuscript?	In which section is the information available? (Reagents and Tools Table, Materials and Methods, Figures, Data Availability Section)
For **antibodies** provide the following information:<br>- Commercial antibodies: RRID (if possible) or supplier name, catalogue number and or/clone number<br>- Non-commercial: RRID or citation	Not Applicable	

DNA and RNA sequences	Information included in the manuscript?	In which section is the information available? (Reagents and Tools Table, Materials and Methods, Figures, Data Availability Section)
**Short novel DNA or RNA including primers, probes**: provide the sequences.	Not Applicable	

Cell materials	Information included in the manuscript?	In which section is the information available? (Reagents and Tools Table, Materials and Methods, Figures, Data Availability Section)
**Cell lines:** Provide species information, strain. Provide accession number in repository **OR** supplier name, catalog number, clone number, and/**OR** RRID.	Not Applicable	
**Primary cultures:** Provide species, strain, sex of origin, genetic modification status.	Not Applicable	
Report if the cell lines were recently **authenticated** (e.g., by STR profiling) and tested for mycoplasma contamination.	Not Applicable	

Experimental animals	Information included in the manuscript?	In which section is the information available? (Reagents and Tools Table, Materials and Methods, Figures, Data Availability Section)
**Laboratory animals or Model organisms:** Provide species, strain, sex, age, genetic modification status. Provide accession number in repository **OR** supplier name, catalog number, clone number, **OR** RRID.	Not Applicable	
**Animal observed in or captured from the field:** Provide species, sex, and age where possible.	Not Applicable	
Please detail **housing and husbandry conditions**.	Not Applicable	

Plants and microbes	Information included in the manuscript?	In which section is the information available? (Reagents and Tools Table, Materials and Methods, Figures, Data Availability Section)
**Plants:** provide species and strain, ecotype and cultivar where relevant, unique accession number if available, and source (including location for collected wild specimens).	Not Applicable	
**Microbes:** provide species and strain, unique accession number if available, and source.	Not Applicable	

Human research participants	Information included in the manuscript?	In which section is the information available? (Reagents and Tools Table, Materials and Methods, Figures, Data Availability Section)
If collected and within the bounds of privacy constraints report on age, sex and gender or ethnicity for all study participants.	Not Applicable	

Core facilities	Information included in the manuscript?	In which section is the information available? (Reagents and Tools Table, Materials and Methods, Figures, Data Availability Section)
If your work benefited from core facilities, was their service mentioned in the acknowledgments section?	Not Applicable	

Design

Study protocol	Information included in the manuscript?	In which section is the information available? (Reagents and Tools Table, Materials and Methods, Figures, Data Availability Section)
If study protocol has been **pre-registered, provide DOI in the manuscript**. For clinical trials, provide the trial registration number **OR** cite DOI.	Not Applicable	
Report the **clinical trial registration number** (at ClinicalTrials.gov or equivalent), where applicable.	Not Applicable	

Laboratory protocol	Information included in the manuscript?	In which section is the information available? (Reagents and Tools Table, Materials and Methods, Figures, Data Availability Section)
Provide DOI OR other citation details if **external detailed step-by-step protocols** are available.	Not Applicable	

Experimental study design and statistics	Information included in the manuscript?	In which section is the information available? (Reagents and Tools Table, Materials and Methods, Figures, Data Availability Section)
Include a statement about **sample size** estimate even if no statistical methods were used.	Not Applicable	
Were any steps taken to minimize the effects of subjective bias when allocating animals/samples to treatment (e.g. **randomization procedure**)? If yes, have they been described?	Not Applicable	
Include a statement about **blinding** even if no blinding was done.	Not Applicable	
Describe **inclusion/exclusion criteria** if samples or animals were excluded from the analysis. Were the criteria pre-established?<br><br>If sample or data points were omitted from analysis, report if this was due to attrition or intentional exclusion and provide justification.	Not Applicable	
For every figure, are **statistical tests** justified as appropriate? Do the data meet the assumptions of the tests (e.g., normal distribution)? Describe any methods used to assess it. Is there an estimate of variation within each group of data? Is the variance similar between the groups that are being statistically compared?	Yes	Figures, Materials and Methods

Sample definition and in-laboratory replication	Information included in the manuscript?	In which section is the information available? (Reagents and Tools Table, Materials and Methods, Figures, Data Availability Section)
In the figure legends: state number of times the experiment was **replicated** in laboratory.	Not Applicable	
In the figure legends: define whether data describe **technical or biological replicates**.	Not Applicable	

Ethics

Ethics	Information included in the manuscript?	In which section is the information available? (Reagents and Tools Table, Materials and Methods, Figures, Data Availability Section)
Studies involving **human participants**: State details of **authority granting ethics approval** (IRB or equivalent committee(s), provide reference number for approval.	Not Applicable	
Studies involving **human participants**: Include a statement confirming that **informed consent** was obtained from all subjects and that the experiments conformed to the principles set out in the WMA Declaration of Helsinki and the Department of Health and Human Services Belmont Report.	Not Applicable	
Studies involving **human participants:** For publication of **patient photos**, include a statement confirming that consent to publish was obtained.	Not Applicable	
Studies involving experimental **animals**: State details of **authority granting ethics approval** (IRB or equivalent committee(s), provide reference number for approval. Include a statement of compliance with ethical regulations.	Not Applicable	
Studies involving **specimen and field samples:** State if relevant **permits** obtained, provide details of authority approving study; if none were required, explain why.	Not Applicable	

Dual Use Research of Concern (DURC)	Information included in the manuscript?	In which section is the information available? (Reagents and Tools Table, Materials and Methods, Figures, Data Availability Section)
Could your study fall under dual use research restrictions? Please check biosecurity documents and list of **select agents and toxins** (CDC): https://www.selectagents.gov/sat/list.htm	Not Applicable	
If you used a select agent, is the security level of the lab appropriate and reported in the manuscript?	Not Applicable	
If a study is subject to dual use research of concern regulations, is the name of the **authority granting approval and reference number** for the regulatory approval provided in the manuscript?	Not Applicable	

Reporting

The MDAR framework recommends adoption of discipline-specific guidelines, established and endorsed through community initiatives. Journals have their own policy about requiring specific guidelines and recommendations to complement MDAR.

Adherence to community standards	Information included in the manuscript?	In which section is the information available? (Reagents and Tools Table, Materials and Methods, Figures, Data Availability Section)
State if relevant guidelines or checklists (e.g., **ICMJE, MIBBI, ARRIVE, PRISMA**) have been followed or provided.	Not Applicable	
For **tumor marker prognostic studies,** we recommend that you follow the **REMARK** reporting guidelines (see link list at top right). See author guidelines, under 'Reporting Guidelines'. Please confirm you have followed these guidelines.	Not Applicable	
For **phase II and III randomized controlled trials**, please refer to the **CONSORT** flow diagram (see link list at top right) and submit the CONSORT checklist (see link list at top right) with your submission. See author guidelines, under 'Reporting Guidelines'. Please confirm you have submitted this list.	Not Applicable	

Data Availability

Data availability	Information included in the manuscript?	In which section is the information available? (Reagents and Tools Table, Materials and Methods, Figures, Data Availability Section)
Have **primary datasets** been deposited according to the journal's guidelines (see 'Data Deposition' section) and the respective accession numbers provided in the Data Availability Section?	Yes	
Were **human clinical and genomic datasets** deposited in a public access-controlled repository in accordance to ethical obligations to the patients and to the applicable consent agreement?	Not Applicable	
Are **computational models** that are central and integral to a study available without restrictions in a machine-readable form? Were the relevant accession numbers or links provided?	Yes	
If publicly available data were reused, provide the respective **data citations in the reference list**.	Yes	Dataset EV5
